# Genome-wide plasma DNA methylation features of metastatic prostate cancer

**DOI:** 10.1172/JCI130887

**Published:** 2020-03-09

**Authors:** Anjui Wu, Paolo Cremaschi, Daniel Wetterskog, Vincenza Conteduca, Gian Marco Franceschini, Dimitrios Kleftogiannis, Anuradha Jayaram, Shahneen Sandhu, Stephen Q. Wong, Matteo Benelli, Samanta Salvi, Giorgia Gurioli, Andrew Feber, Mariana Buongermino Pereira, Anna Maria Wingate, Enrique Gonzalez-Billalebeitia, Ugo De Giorgi, Francesca Demichelis, Stefano Lise, Gerhardt Attard

**Affiliations:** 1 University College London Cancer Institute, London, United Kingdom.; 2 Centre for Evolution and Cancer, The Institute of Cancer Research, London, United Kingdom.; 3 Istituto Scientifico Romagnolo per lo Studio e la Cura dei Tumori (IRST) IRCCS, Meldola, Italy.; 4 Centre for Integrative Biology, University of Trento, Trento, Italy.; 5 Peter MacCallum Cancer Centre and the University of Melbourne, Melbourne, Victoria, Australia.; 6 Servicio de Hematología y Oncología Médica, Hospital Universitario Morales Meseguer, IMIB-Universidad de Murcia, Murcia, Spain.; 7 Institute of Computational Biomedicine, Weill Cornell Medicine, New York, New York, USA.

**Keywords:** Genetics, Oncology, Cancer, Epigenetics, Prostate cancer

## Abstract

Tumor DNA circulates in the plasma of cancer patients admixed with DNA from noncancerous cells. The genomic landscape of plasma DNA has been characterized in metastatic castration-resistant prostate cancer (mCRPC) but the plasma methylome has not been extensively explored. Here, we performed next-generation sequencing (NGS) on plasma DNA with and without bisulfite treatment from mCRPC patients receiving either abiraterone or enzalutamide in the pre- or post-chemotherapy setting. Principal component analysis on the mCRPC plasma methylome indicated that the main contributor to methylation variance (principal component one, or PC1) was strongly correlated with genomically determined tumor fraction (*r* = –0.96; *P* < 10^–8^) and characterized by hypermethylation of targets of the polycomb repressor complex 2 components. Further deconvolution of the PC1 top-correlated segments revealed that these segments are comprised of methylation patterns specific to either prostate cancer or prostate normal epithelium. To extract information specific to an individual’s cancer, we then focused on an orthogonal methylation signature, which revealed enrichment for androgen receptor binding sequences and hypomethylation of these segments associated with *AR* copy number gain. Individuals harboring this methylation pattern had a more aggressive clinical course. Plasma methylome analysis can accurately quantitate tumor fraction and identify distinct biologically relevant mCRPC phenotypes.

## Graphical abstract

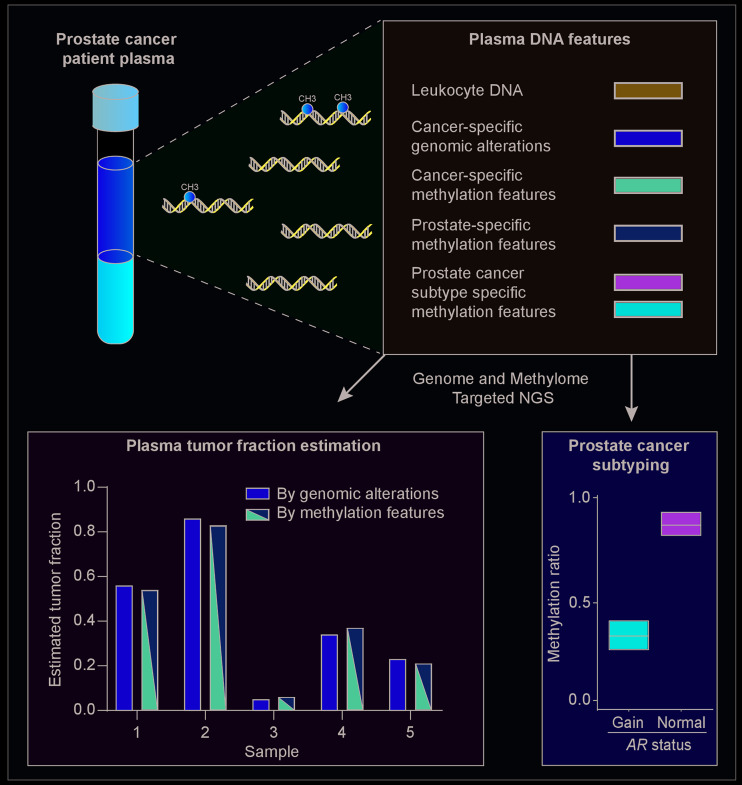

## Introduction

Analysis of plasma DNA somatic point mutations or copy number alterations through liquid biopsy has potential utility for informing treatment decisions in cancer patients with a range of tumor types (1, 2). Several studies have shown that plasma DNA is representative of clinically relevant metastases (3). In addition to genomic information, plasma DNA also contains methylation information that could be concurrently extracted. Methylation status is tissue-specific and can be used to interrogate cellular components and quantitate tumor composition in tissues (4, 5). Several studies to date have used methylation information from plasma DNA for early detection of cancer and to identify cancer tissue of origin (6–14), but the plasma DNA methylome has not been as extensively characterized in metastatic cancer patients. Methylation markers identified from cell lines or tissues can be used for tracking prostate cancer in plasma but may miss differences that occur as a result of plasma DNA composition, which could be important due to the complexity of tissue-specific methylation patterns (15).

Metastatic castration-resistant prostate cancer (mCRPC) exhibits a variable clinical course and biomarkers to stratify patients are urgently required to optimize management. mCRPC patients with a range of genomic aberrations, including androgen receptor (*AR*) gene copy number gain or *TP53* mutations, detected in plasma prior to androgen receptor (AR) targeting with abiraterone or enzalutamide have a shorter duration of treatment benefit and overall survival (16–20). Recent integration of genomics, methylation, and expression from tumor biopsies has identified methylation changes as a key component in the transition of mCRPC to a more aggressive, androgen-insensitive phenotype (21). However, tumor biopsies from metastatic sites can be difficult to obtain and repeated sampling of multiple metastases is usually not feasible, limitations that could be addressed by minimally invasive liquid biopsy. To concurrently study the plasma genome and methylome and overcome the inherent challenges of methylation analysis resulting from the high variance in methylation data, we selected plasma samples from a focused cohort of mCRPC patients with genomic information. We hypothesized that integration of methylation and genomic data extracted concurrently from metastatic cancer patient plasma DNA using next-generation sequencing (NGS) could improve patient stratification by identifying clinically relevant subtypes. We aimed to profile mCRPC methylation features and interrogate clinical utility in plasma from patients treated with standard-of-care abiraterone or enzalutamide.

## Results

### Interrogating the plasma DNA methylome in metastatic prostate cancer.

**Figure 1:**
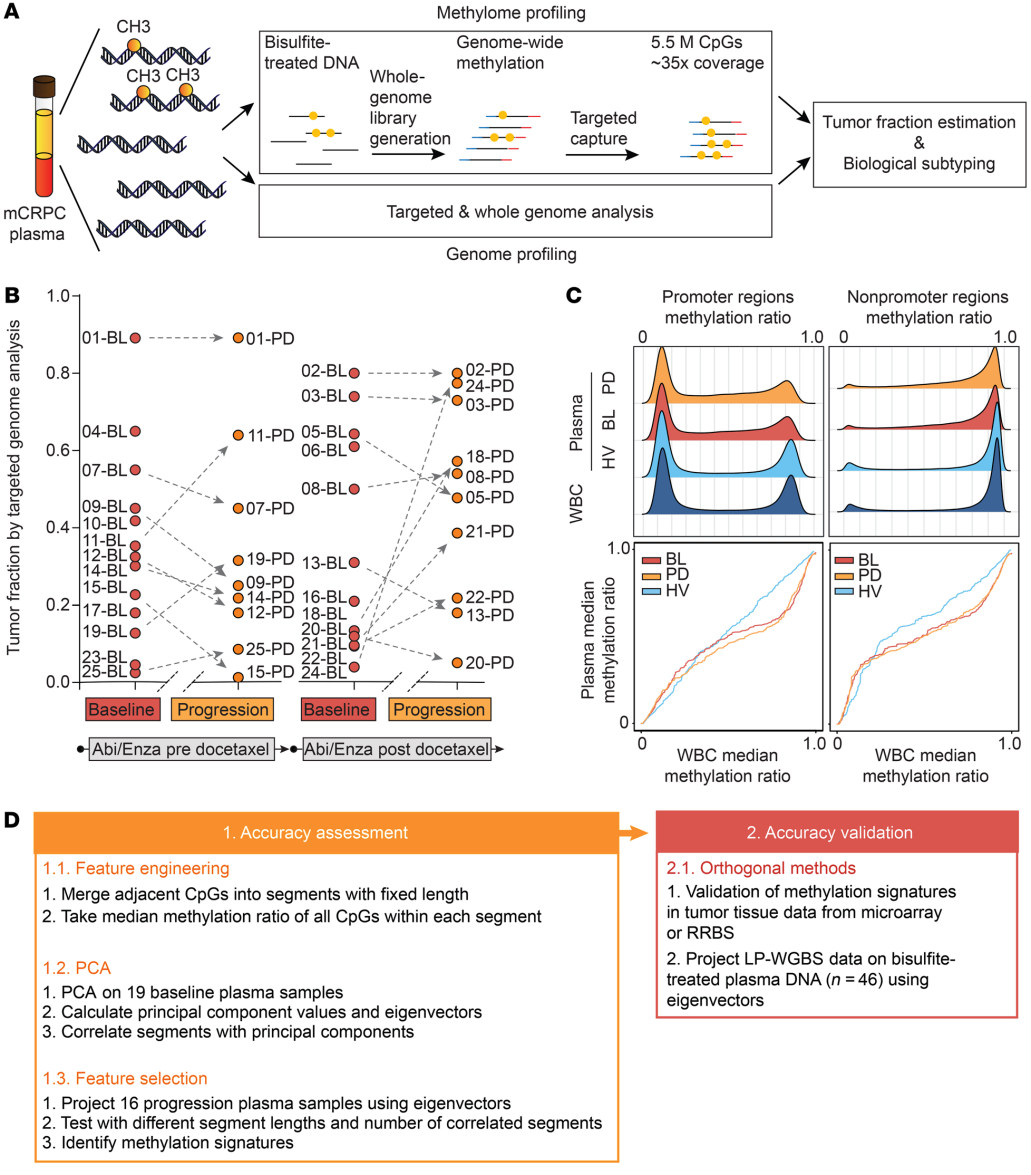
The mCRPC plasma methylome. (**A**) Schematic overview of the workflow for integrating NGS of the plasma methylome and genome. (**B**) Genomically determined tumor fraction in baseline and progression samples from pre- and post-chemotherapy patients receiving abiraterone or enzalutamide. (**C**) Methylation ratio density (upper panel) and quantile-quantile plot (Q-Q plot, bottom panel) analysis based on the genomic annotation of methylation segments in promoter or other regions. Data from white blood cells (WBC) or plasma collected at baseline (BL) or progression (PD) from mCRPC patients or from healthy volunteers (HV) are presented separately. (**D**) Schematic workflow of methylation data analysis.

We concurrently characterized the mCRPC plasma methylome and genome (Figure 1A). Plasma DNA was subjected to either high-coverage targeted or whole-genome NGS in order to determine tumor fractions and copy number status. Tumor fractions were derived using genomic information at heterozygous single-nucleotide polymorphisms (SNPs) to computationally determine the abundance of deletions involving 8p21 or 21q22, designated as prostate cancer anchor lesions that we had used previously as a proxy for tumor fraction (22, 23). We collected plasma within 30 days of abiraterone or enzalutamide (baseline) administration from 25 mCRPC patients (median age: 76 years; range: 42–90 years) representing a wide range of genomically determined tumor fractions and from across the disease spectrum (docetaxel-naive or docetaxel-treated) who were participating in prospective biomarker protocols. Of the 25 patients, 19 also had plasma collected at radiographic progression (Figure 1B and Supplemental Table 1; supplemental material available online with this article; https://doi.org/10.1172/JCI130887DS1). The median and range of genomically determined tumor fractions in our mCRPC cohort were 0.41 (0.04–0.89) and 0.42 (0.09–0.89) for baseline and progression plasma, respectively.

We subjected a separate aliquot of DNA to bisulfite treatment and performed target enrichment NGS for 5.5 million pan-genome CpG sites (target coverage: at least ×30; key sequencing parameters in Supplemental Table 2). These CpGs were selected based on their known involvement in or proximity to regions that had been associated with cancer (see Supplemental Methods). In total, we performed targeted capture on 39 plasma samples (19 baseline, 16 progression, 4 plasma samples from 2 healthy male individuals, ages 30 and 60 years, Supplemental Figure 1 and Supplemental Table 2). We also performed low-pass whole-genome bisulfite sequencing (LP-WGBS) on 46 plasma samples (Supplemental Figure 1 and Supplemental Table 3). Additionally, we conducted targeted bisulfite NGS on 15 white blood cell samples, including white blood cells collected prior to and 108 days after treatment with abiraterone from one patient (Supplemental Table 1).

Adjacent CpG methylation patterns are usually highly correlated (8, 13). We therefore applied a 100-base-pair sliding window and divided our data into 1.47 million methylation segments (Supplemental Methods). In keeping with prior studies on tissues, the methylation ratio distribution across all methylation segments in plasma and white blood cell samples showed a density peak for hypermethylation and hypomethylation (Supplemental Figure 2). We selected regions with a minimum of ×10 coverage. When separated by annotation category (such as promoter, exon, intron), the distribution was consistent with the targeted regions (Supplemental Figure 3) (24). We observed that methylation segments in promoter regions were primarily hypomethylated whereas other categories were primarily hypermethylated (Figure 1C). We then compared the methylation ratio distribution in baseline, progression plasma, and healthy volunteer plasma with white blood cell DNA, and we observed significant differences among plasma and white blood cell samples (*P* < 10^–15^, Kruskal-Wallis test). The difference was more pronounced in plasma samples from cancer patients compared with healthy volunteers (*Z* scores for promoter regions were –20.3, –20.7, and –13.1 and for nonpromoter regions were –154.3, 167.9, and –6.0; all *P* < 10^–9^, Dunn’s test; Figure 1C). In keeping with previous studies that the cancer genome is characterized by more hypomethylation events (25–27), the mCRPC plasma methylome that includes a mixture of cancer and normal DNA is globally more hypomethylated than healthy volunteer plasma.

### An unbiased approach identifies tumor fraction as the major determinant of global plasma DNA methylation variance.

**Figure 2:**
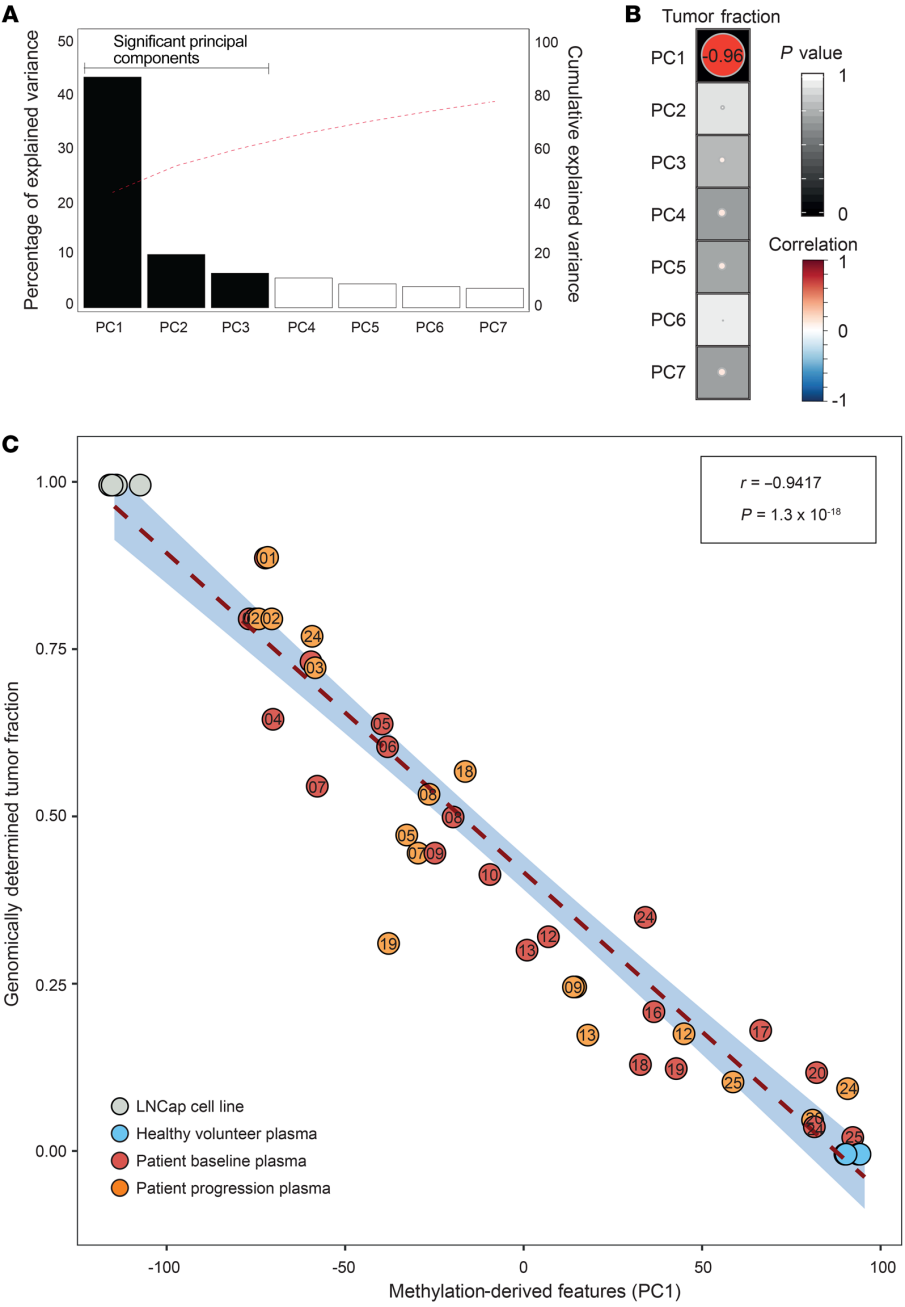
Tumor fraction is the major determinant of the plasma methylome. (**A**) Bar chart shows the variance associated to each principal component (PC) on 19 baseline samples; the red dotted line indicates cumulative explained variance. (**B**) Correlation between PCs and tumor fraction. Size and the color of each circle show Pearson correlation and background shading denotes *P* value). (**C**) Correlation of genomically determined tumor fraction (*y* axis) and PC1 values (*x* axis) from high-coverage targeted methylation sequencing on 19 baseline samples, 16 progression plasma samples, and control samples (*n* = 4 healthy volunteer plasma samples, LNCaP prostate cancer cell line).

We used an unbiased analytical framework to explore the complexity of pan-genome plasma methylation changes (Figure 1D). We performed principal component analysis (PCA) on the 19 baseline samples. The first principal component (PC1) contributed 42% of the variance (Figure 2A) and showed a high correlation with genomically determined tumor fraction (*r* = –0.96, *P* = 1.3 × 10^–10^, Pearson correlation; Figure 2B). To investigate whether treatment with AR targeting agents affected the association of PC1 with tumor fraction, we used PCA eigenvectors to project the progression samples, healthy volunteer controls (0 tumor fraction), and the LNCaP prostate cancer cell line (100% tumor, 3 replicates; Figure 2C). After including the projected samples, the correlation of PC1 and genomically determined tumor fraction remained high (*r* = –0.94, *P* = 1.3 × 10^–18^; Figure 2C).

To evaluate the clinical applicability of our findings, we then extracted scaled PC1 values from LP-WGBS. Applying Bland-Altman analysis, we found a good agreement between LP-WGBS–derived tumor fraction estimation and estimates from high-coverage targeted NGS (95% limits of agreement: –0.25–0.15, bias: –0.05), introducing the opportunity for scalable and cost-efficient circulating tumor DNA detection and quantitation using LP-WGBS (Supplemental Figure 4).

### Methylation ratio can serve as a proxy for tumor fraction.

**Figure 3:**
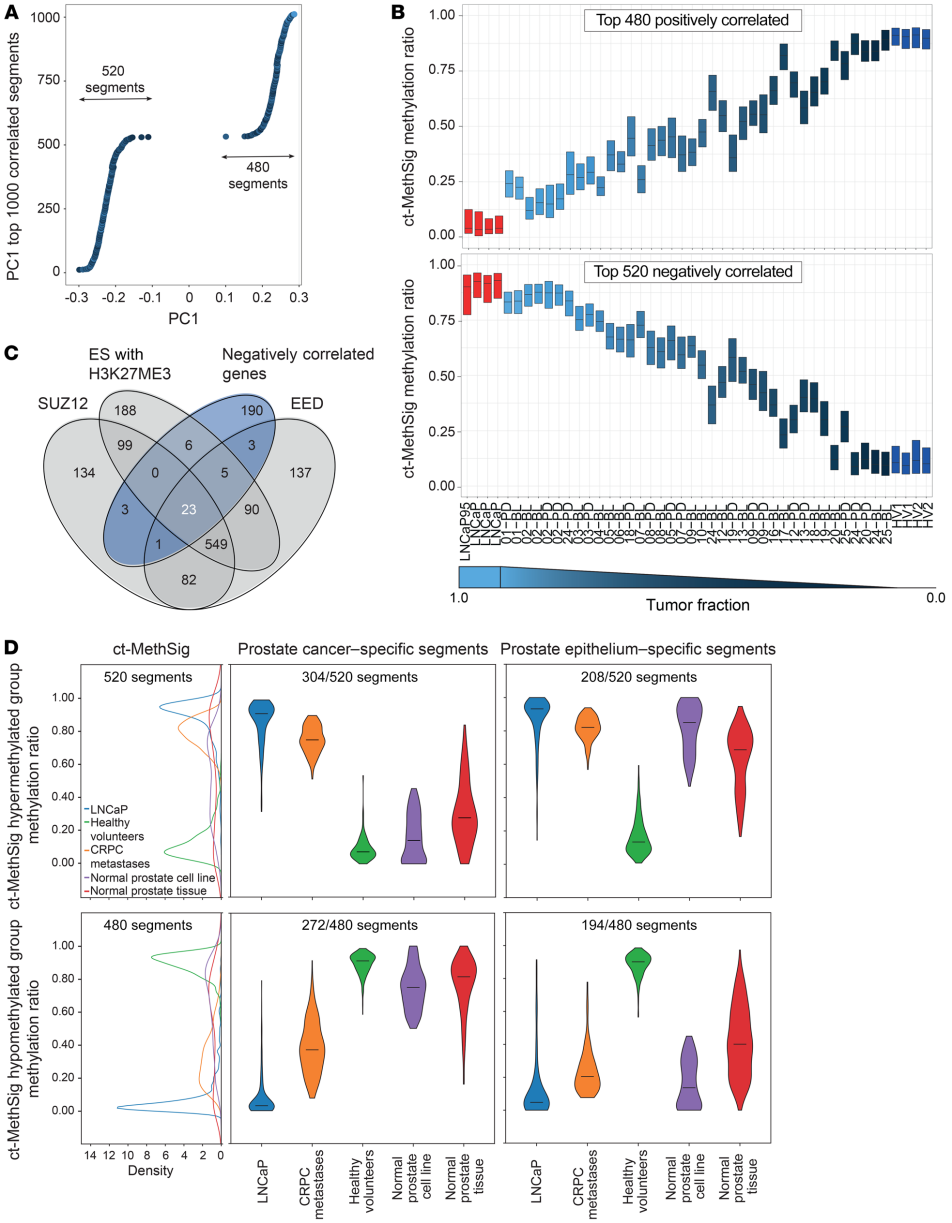
Methylation ratio across ct-MethSig can be a proxy for tumor fraction. (**A**) Top 1000 segments (ct-MethSig) with the highest correlation coefficient between PC1 and methylation ratio. (**B**) ct-MethSig methylation ratio distribution by patient plasma sample split by negatively correlated and positively correlated segments. (**C**) Venn diagram showing the overlap of negatively correlated genes (dark blue) in ct-MethSig segments with targets of EED, SUZ12, and embryonic stem cells (ES) with H3K27ME3 marks. The number in white denotes the number of genes in the ct-MethSig negatively correlated group. (**D**) Circulating tumor fraction methylation signature comprises segments specific to either normal or malignant prostate epithelium. Left: Methylation ratios of ct-MethSig hypermethylated (*n* = 520) and hypomethylated (*n* = 480) groups from LNCaP (*n* = 4), healthy volunteers (*n* = 4), and normal prostate epithelium samples (PrEC). Right: The ct-MethSig hypermethylated and hypomethylated groups can be split into prostate cancer–specific segments and prostate epithelium–specific segments.

To test features identified by NGS in data sets with fewer data points, such as methylation arrays, we hypothesized that the median of methylation ratios of segments that most strongly correlated to the component features could serve as a proxy of tumor fraction. We consistently observed a high correlation (*r* = 0.93, Pearson correlation) of median methylation ratio with genomically determined tumor fraction in both negatively and positively correlated group when including 10 to 10,000 segments. Also, the intrasample variance of methylation ratios in the top-correlated segments gradually increased when we included more segments (Supplemental Figures 5 and 6). We therefore selected the 1000 segments that showed the highest correlation with PC1 (hereafter referred to as circulating tumor methylation signature or ct-MethSig; Figure 3A). We confirmed that the median of ct-MethSig methylation ratios showed a high correlation with tumor fraction (520 segments in negatively correlated regions or ct-MethSig, hypermethylated group: *r* = 0.95, *P* = 8.4 × 10^–19^; 480 segments in positively correlated regions or ct-MethSig, hypomethylated group: *r* = –0.93, *P* = 3 × 10^–16^, Pearson correlation; Figure 3B). Additionally, ct-MethSig did not include genes whose methylation status has been previously reported as diagnostic of prostate cancer in tissue (28), as the segments overlapping with these genes were not as strongly correlated with PC1 value (Supplemental Figure 7).

Additionally, we tested this finding in published tissue data sets and confirmed a high correlation with tumor purity both in mCRPC (21) (hypermethylated group: *r* = 0.92, *P* < 1.5 × 10^–6^; hypomethylated group: *r* = –0.74, *P* < 1.4 × 10^–3^, Pearson correlation; Supplemental Figure 8), and hormone-sensitive prostate cancer (HSPC) (29) (hypermethylated group: *r* = 0.91, *P* < 10^–60^; hypomethylated group: *r* = –0.61, *P* < 10^–17^, Pearson correlation; Supplemental Figure 9).

### Functional enrichment identifies hypermethylation of polycomb repressor complex 2 targets in circulating prostate cancer DNA.

To study the biological processes underlying PC1, we performed gene set enrichment analysis (GSEA) on genes overlapping with ct-MethSig segments. We observed significant enrichment (adjusted *P* < 10^–4^) for targets of the polycomb repressor complex 2 (30) (PRC2-related category in the Molecular Signature Database or MSigDB, Table 1) that was of particular interest, as a previous mRNA profiling study showed that prostate cancer was distinguished from noncancer prostate epithelium by downregulation of genes that are repressed by PRC2 (31). We noted that these PRC2 genes were only in the ct-MethSig hypermethylated group, representing an increase in methylation ratio with increasing fraction. Overall, the 520 negatively correlated segments included 231 genes. Of these, 41 were collectively either components of PRC2-EED (embryonic ectoderm development) (32) and SUZ12 (suppressor of zesta 12) (33) or H3K27ME3 (trimethylation of lysine 27 on histone H3 protein subunit) (Figure 3C). We performed a permutation test and the result indicated that PRC2-regulated components were more enriched in ct-MethSig as compared with 1000 randomly selected genomic segments (Supplemental Figure 10). Our discovery of hypermethylation in promoters upstream of these genes provides a biological explanation for their downregulation and introduces a strategy for extending this biological difference to a liquid biopsy application (21, 31).

### The circulating tumor methylation signature comprises segments specific to either normal or malignant prostate epithelium.

We posited that ct-MethSig included components that were specific to either malignant or nonmalignant prostate epithelium. We plotted the kernel density estimation of the ct-MethSig methylation ratios in whole-genome bisulfite sequencing data derived from the nonmalignant prostate epithelium cell (PrEC) line (34), and we observed that there was a bimodal distribution (Figure 3D). We therefore adapted the Gaussian mixture model on methylation ratios of ct-MethSig segments from the prostate cancer cell line LNCaP and our 2 healthy volunteer plasma samples, and then we used the fitted Gaussian distribution on normal PrECs. In PrECs, we identified segments whose methylation ratio distribution aligned with either LNCaP or healthy volunteer plasma. We concluded that the former segments with methylation ratios in normal prostate epithelium similar to LNCaP were prostate epithelium–specific, while the segments with methylation ratios similar to healthy volunteer plasma were prostate cancer–specific (Figure 3D). We then confirmed these findings by showing that CRPC metastases (bone, bladder, liver, and lymph nodes, described further in Supplemental Table 4) included segments attributed to both normal and cancerous prostate epithelium, whereas normal prostate (54-year-old male donor, ENCODE donor ID: ENCDO451RUA) included only segments attributable to normal prostate epithelium. As a result, we could split ct-MethSig into 2 components, circulating prostate cancer–specific and normal prostate-specific signatures. Finally, we used methylation microarray data from 553 prostate cancers from TCGA and 12 CRPC adenocarcinoma from Beltran et al. (21) to show that the distribution of ct-MethSig segments in localized prostate cancer and CRPC tissue includes both cancer and normal components (Supplemental Figure 11).

### Methylation signatures specific to an individual’s cancer.

**Figure 4:**
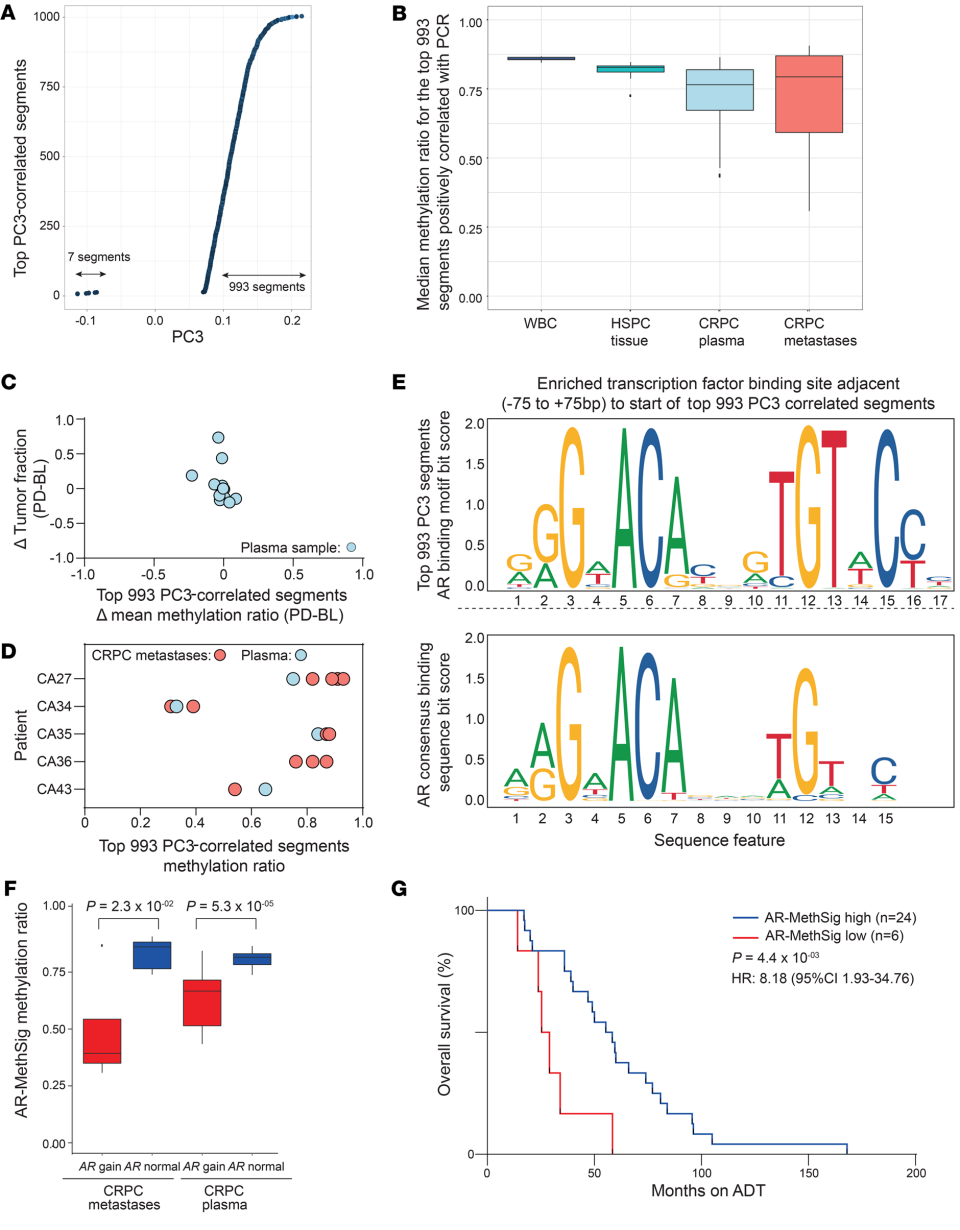
Methylation signatures that could allow subgrouping of mCRPC. (**A**) Top 1000 segments with the highest correlation coefficient between PC3 and methylation ratio. (**B**) Methylation ratio of top 1000 segments highly correlated with PC3 values derived from plasma, white blood cell, HSPC tumor, and CRPC tumor (CASCADE trial). (**C**) Comparison of intraindividual changes in the top-correlated segments defined by targeted methylation NGS on plasma DNA and changes in tumor fraction. The *y* axis denotes the difference (Δ) of mean methylation ratio of the top-correlated segments between baseline and progression samples and the *x* axis denotes the difference in tumor fraction. (**D**) Median methylation ratio of the top-correlated segments of different metastatic sites by patient from the CASCADE rapid warm autopsy program. (**E**) AR binding motif that is overrepresented in regions adjacent to the top correlated segments (top). The consensus AR binding motif is shown as a reference (bottom). (**F**) Methylation ratio of AR-MethSig segments of *AR* gain group (CRPC metastases *n* = 5, CRPC plasma *n* = 18) and nongain group (CRPC metastases *n* = 8, CRPC plasma *n* = 17; Mann-Whitney *U* test). (**G**) Overall survival analysis (start of ADT to death) for AR-MethSig low group versus AR-MethSig high group (Mantel-Cox log-rank test).

We were next interested in plasma DNA methylation changes that could potentially identify distinct methylation subtypes. The second principal component was driven by a single patient (patient 02), so we have not investigated further. We focused on the third principal component, where we found only a weak correlation with tumor fraction (*r* = 0.01, *P* = 0.96, Pearson correlation) (Figure 2B). Similar to the methodology applied to ct-MethSig, we first identified the top 1000 segments that were most correlated with this component’s values. In contrast to ct-MethSig, these were predominantly positively correlated (Figure 4A). Using the median of every segment’s methylation ratio, we were able to incorporate array-based methylation data from biopsies from intermediate-risk HSPC (29) and mCRPC (21). We found that the median methylation ratio in CRPC plasma and tumor samples presented a greater variability in contrast to HSPC or white blood cells (Figure 4B and Supplemental Figure 12). We noted that, in contrast to ct-MethSig, a change in tumor fraction before and after treatment did not change the median methylation ratio of the top-correlated segments with PC3 (Figure 4C). Similarly, interpatient differences were greater than intrapatient variability in multiple metastases and plasma harvested from the same patient at autopsy (Figure 4D and Supplemental Table 4).

Functional enrichment analysis on the top 1000 segments showed enrichment in histone H3 trimethylation markers (Supplemental Tables 5 and 6). We hypothesized that this methylation signature was regulated by a common transcriptional pathway. Therefore, we searched for known transcriptional factor binding sites (TFBSs) adjacent to within 75 base pairs of the start of the top 1000 segments, using a protocol described previously (35). Notably, the AR binding motif was the only significantly overrepresented binding site (local enrichment *P* = 6 × 10^–4^, global enrichment *P* = 3 × 10^–16^; Figure 4E and Supplemental Table 7). We denoted this profile AR-MethSig.

### AR-MethSig hypomethylation strongly associates with AR copy number gain.

Next, we extracted genome-wide copy number profiles from LP-WGS and confirmed high similarity among results from the same sample with and without bisulfite treatment (Supplemental Figure 13). Using LP-WGBS from plasma samples, we observed copy number alterations at a frequency consistent with previously described studies of mCRPC tissue or plasma (20, 36) (for example, most commonly: 8q21-24 gain: prevalence ≥70%; Xq12 gain: prevalence ≥60%; 8p21 loss: prevalence ≥50%, Supplemental Figure 14). We observed more copy number changes with increasing PC1 values, as an increasing tumor fraction improved copy number detection (Supplemental Figure 15). We then confirmed that ct-MethSig or AR-MethSig was not located more frequently in regions of copy number alterations (Supplemental Table 8). To integrate genomic copy number data with specific methylation signatures, we evaluated the correlation of the copy number of every segment across the genome and PC1 values (Kruskal-Wallis test, Supplemental Figure 16). Most notably, we identified a significant difference in PC3 value distributions when comparing *AR* copy number gain and *AR* nongain samples (*P* = 0.018, Kruskal-Wallis test, Supplemental Figure 17). Given the association of PC3 values with *AR* copy number, we confirmed that patient plasma and tissue samples with *AR* gain had a significantly lower AR-MethSig methylation ratio than *AR* copy number normal samples (*P* < 0.001 and *P* = 0.023, respectively, Wilcoxon signed-rank test; Figure 4F).

### The AR-regulatory methylation signature may identify distinct clinical phenotypes.

We found a high agreement for the median methylation ratio of AR-MethSig extracted from high-coverage targeted NGS and LP-WGBS (95% limits of agreement: –0.136–0.076; Supplemental Figure 18), again supporting the use of LP-WGBS, which is amenable to clinical implementation for methylation-based patient stratification. We did not identify any hormone-sensitive cancers harboring a low AR-MethSig median methylation ratio. Likewise, neither of the 2 commonly studied AR-regulated prostate cancer cell lines (LNCaP and VCaP, Supplemental Figure 12) harbored a low AR-MethSig median methylation ratio. We were therefore interested in evaluating the clinical relevance of AR-MethSig, and as we had not observed a change over time in AR-MethSig median methylation ratio, we chose fixed time points over the disease independent of the time of sampling: namely time from start of ADT to death. We observed that AR-MethSig low cancers had poor clinical prognosis (HR = 8.18, 95% CI = 1.93–34.76, *P* = 0.0044; Mantel-Cox log-rank test; Figure 4G).

## Discussion

Here we characterize the plasma methylome in mCRPC and identify prostate cancer–specific methylation signatures. By using a 100-base-pair sliding window strategy, we obtained close to 0.5 million methylation segments in all of the baseline plasma DNA samples subject to custom targeted enrichment NGS, and we used them to construct our PCA. What we believe is novel to our approach was the construction of our model using solely mCRPC plasma DNA with a wide range of tumor fractions. These had a variable ratio of normal DNA primarily arising from white blood cells (14), and tumor DNA that harbors methylation changes that we found are either prostate epithelium–specific or cancer-specific. By using the median methylation ratio of ct-MethSig (segments that highly correlated with PC1), we were able to implement our signature in methylation data with variable CpG coverage, including methylation microarrays or reduced representation bisulfite sequencing. Ct-MethSig did not include genes widely known to be hypermethylated in prostate cancer, such as *GSTP1* (28, 37). This finding could be explained by highly variable methylation levels at these loci in noncancerous plasma DNA.

Because the majority of methylation features extracted from plasma DNA are related to tumor fraction, extracting methylation information specifically related to an individual’s cancer could be challenging across a range of tumor fractions, as seen in clinical practice and exemplified in our cohort. Higher coverage NGS may address this challenge, with capture of sufficient tumor-specific reads even at low circulating tumor. Tissue methylation has been used for subtyping in other cancer types, such as brain tumors (38). To date, methylation NGS data on large mCRPC cohorts linked to clinical outcomes remains limited; international efforts have focused on obtaining genomic and transcriptomic data from tumor biopsies (21, 36, 39). In the study by Beltran et al., selected methylation markers from CRPC patients were used to classify tumors with neuroendocrine differentiation. Pan-genome copy numbers and methylation patterns in cancer tissues can be profiled concurrently and coevolve in advanced prostate cancer (40–42). Here, we identified AR-MethSig spanning 1000 genomic regions in the mCRPC plasma; these segments appear to identify a subgroup of cancers characterized by a more aggressive clinical course and enriched for *AR* copy number gain. Interestingly, we also found that they had hypomethylation at putative AR binding sites. Previous studies have reported worse outcome for patients with *AR* gain in plasma (16, 17) and given the high overlap between this genomic lesion and AR-MethSig, it is possible that our methylation signature identifies the same phenotype. Studies in more prostate cancer patients across the disease spectrum and healthy volunteers are required to validate our methylation subtyping signatures and confirm response prediction.

Our study identified methylation changes in 1000 genomic segments that can be used to track circulating tumor DNA. This could address some of the challenges inherent in plasma genomic studies, including the paucity of common genomic events (20, 36) and clonal hematopoiesis in older populations (43), that limit sensitivity and population-based testing. The plasma methylome could therefore represent an important source of information that complements or replaces genomic testing. In conclusion, our study uses methylation features from plasma DNA to track circulating prostate cancer DNA and identify subtypes of mCRPC with distinct biological mechanisms and differential clinical outcomes.

## Methods

### Study design.

Plasma samples were collected within 30 days of treatment initiation and at progression in 3 biomarker studies. These cohorts have been described previously (16, 17) (Supplemental Table 1). Briefly, patients needed to have histologically or biochemically confirmed prostate adenocarcinoma and be starting abiraterone or enzalutamide for progressive mCRPC. Patients were required to receive abiraterone or enzalutamide until disease progression, as defined by at least 2 of the following: a rise in PSA, worsening symptoms, or radiological progression defined as progression in soft-tissue lesions measured by computed tomography (CT) imaging according to modified Response Evaluation Criteria in Solid Tumors or progression on bone scanning according to criteria adapted from the Prostate Cancer Clinical Trials Working Group 2 guidelines. In keeping with this being a discovery analysis in the roadmap to development of a methylation-based biomarker, we prioritized patients with sufficient vials to allow both genome and methylome assessment. Metastases were obtained at rapid warm autopsy in the Peter MacCallum warm autopsy program CASCADE (Cancer Tissue Collection After Death, Supplemental Table 2) (44).

### Plasma DNA sequencing.

Circulating DNA (10–25 ng) was extracted from plasma using the QIAamp Circulating Nucleic Acid kit (Qiagen) and quantified using the Quant-iT high-sensitivity Picogreen double-stranded DNA Assay Kit (Invitrogen by Thermo Fisher Scientific). Germline DNA was extracted from white blood cells using the QIAamp DNA kit (Qiagen). Genomic NGS was performed as described previously (16). For methylation assessment, raw plasma DNA was bisulfite treated using the ZYMO Gold Kit per the manufacturer’s protocol. We adapted Swift Bioscience Methyl-Seq to generate libraries. CpGs were selected from prior data generated using the Illumina Infinium Human Methylation 450k microarray (Roche Nimblegen targeted capture kit, Epi CpGiant). Probes were designed to hybridize to strands of fully methylated, partially methylated, and fully unmethylated derivatives of the target (Supplemental Material). Libraries were quantified by KAPA library quantification kit (Roche) before pooling and sequencing on an Illumina HiSeq 2500 using paired-end 100-base-pair reads. Sequencing matrices for targeted methylome and LP-WGBS are included in Supplemental Tables 2 and 3, and details on the pipelines for analysis of sequencing data are provided in Supplemental Methods.

### Principal component analysis of targeted plasma methylome.

Methylation segments with methylation ratios available in all baseline samples (*n* = 19) and standard deviation values included in the upper 2 quartiles were subjected to principal component analysis (FactorMineR R package v1.41) (45). Significant principal components were determined using a permutation test as implemented in the jackstraw R package (v1.2) (https://CRAN.R-project.org/package=jackstraw). The projection of all the samples based on the PCA eigenvectors was based on the methylation ratio of regions used in the initial PCA for all the samples. Missing values were imputed based on the PCA method as implemented in the missMDA R package (v1.13) (46).

### Tumor fraction estimation.

Genomically determined tumor fraction was determined from targeted NGS using CLONET as described previously (16, 22). On high-coverage targeted methylation NGS, we calculated PC1 values as described above, and the median of PC1 values extracted from healthy volunteers was set as 0%, whereas the median of PC1 values derived from LNCaP samples was set as 100% tumor purity. The tumor fraction of all the plasma samples was obtained with interpolation using PC1 projected values. For tumor fraction estimation based on LP-WGS on bisulfite-treated or nontreated plasma DNA, we used ichorCNA (47) (Supplemental Methods). For LP-WGBS, we also used PC1 projected values.

### Statistics.

Pearson correlation was used to measure the association between 2 parameters (principal component values versus genomically determined tumor fraction estimation, or different approaches of tumor fraction estimations). The association between copy number status of each region and principal components was estimated using the Kruskal-Wallis test. Mann-Whitney *U* test was used to test significance between 2 groups (*AR* gain versus *AR* nongain; AR-MethSig high group versus AR-MethSig low group). Hazard ratio in overall survival analysis was calculated using the Mantel-Haenszel method. For all tests, a significance threshold of 0.05 was required unless otherwise specified.

### Data availability.

The data that support the findings of the study have been deposited in the European Genome-phenome Archive (EGA study ID: EGAS00001003958).

### Study approval.

Plasma samples were separately approved by the Istituto Scientifico Romagnolo per lo Studio e la Cura dei Tumori (IRST), Meldola, Italy (REC 2192/2013), the Royal Marsden, London, United Kingdom (REC 04/Q0801/6), and the PREMIERE trial (EudraCT: 2014-003192-28, NCT02288936) that was sponsored and conducted by the Spanish Genito-Urinary Oncology Group (SOGUG). Metastatic samples obtained were approved by the Peter MacCallum warm autopsy program, CASCADE (HREC 15/98). All patients provided written informed consent for these analyses.

## Author contributions

AW and GA designed the project. AW, DW, VC, AJ, GG, and AMW performed DNA extractions and sequencing. AW and PC designed and implemented the computational strategy and statistical analysis with input from AF and SL. MBP optimized copy number profiling and tumor fraction estimation. DK and SL performed whole-genome sequencing analysis and gave critical feedback on the statistical workflow. GMF, MB, and FD contributed to genomic determination of tumor content and integrated methylation data from mCRPC data sets. VC, AJ, S Sandhu, SQW, S Salvi, EGB, UDG, and GA contributed patients and obtained samples. AW, PC, DW, and GA wrote the manuscript and the other authors provided critical comments. All authors read and approved the final manuscript. GA was responsible for the overall project.

## Supplementary Material

Supplemental data

## Figures and Tables

**Table 1:**
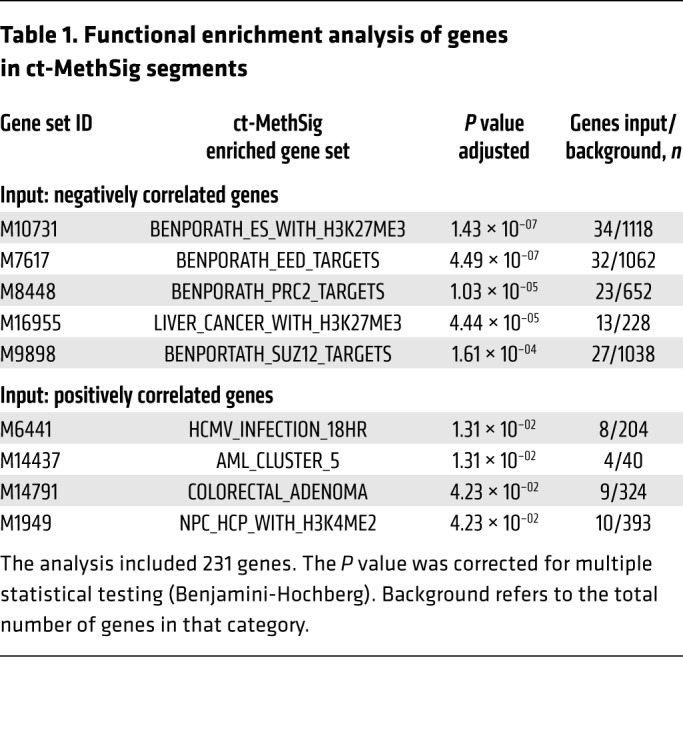
Functional enrichment analysis of genes in ct-MethSig segments

## Version 1 03/09/2020

Electronic publication

## Version 2 04/01/2020

Print issue publication

## Version 3 06/15/2020

Copyright statement corrected in PDF
