# Supplementary material for: Genome-wide plasma DNA methylation features of metastatic prostate cancer
Source: J Clin Invest. 2020 Mar 9;130(4):1991–2000. doi: 10.1172/JCI130887 (PMC7108919; doi:10.1172/JCI130887)
Supplement: Supplemental data [file jci-130-130887-s006.pdf]

Supplementary Methods

1. Processing of targeted methylation NGS data

Data were processed using fastqc to assess quality and read through adapters were trimmed using Trimmomatic v0.36. Since DNA was bisulfite treated, we aligned the reads based on three nucleotides (thymine(T), adenosine(A), guanine(G)) to the human genome (hg)19 using the BSMAP v2.90 (1, 2). The duplicated reads were removed with Picard tools v2.1.0 (http://broadinstitute.github.io/picard), and unaligned reads were clipped (hard-clipped) using the bamUtil 1.0.13(3).

The CpG methylation ratio of each loci was calculated using formula (I), which takes cytosine(C) and thymidine(T) counts from all reads covering each CpG loci.

$$Methylation\ Ratio = \frac{C}{C+T} \quad \text{(I)}$$

From all sites included in our predesigned capture panel (Roche Nimblegen SeqCap EpiGiant), only sites with a minimum coverage of 10X were considered for further analysis of CpG (**Supplementary Fig. S1**). The methylation ratio was computed using the methylKit R package v1.6.2(4).

2. Selection of optimal data inputs for PCA

Adjacent CpG methylation levels are usually highly related, and previously studies have demonstrated high sensitivity of identifying tissue-specific methylation markers using sliding window approaches (5-7). Here we combined adjacent CpG sites into methylation segments of fixed length, and the median methylation ratio across all CpGs within the segment was used to represent the methylation ratio of the segment using methylKit R package v1.6.2(4). Initially we used 100bps with sliding window of 50bps and generated > 1.47 million windows across all CpGs in our target panel. We applied principal component analysis (PCA) using the FactoMineR v1.41 package.

To eliminate potential biases due to the selection of segmentation length, we optimised the segmentation length parameter. To do so, we tested segments of 10bps, 100bps, 1000bps and 10,000bps with sliding windows of 5bps, 50 bps, 500 bps and 5000 bps, respectively. We found that the smaller the window size, the more data we had to drop when combining plasma samples due to variable inputs and sequencing coverage (**Supplementary Fig S19**). We also found that the methylation ratio of 100bps segments with 50 bps sliding window shows high consistency with the methylation ratio estimated at single CpG level (**Supplemental Fig S20**). The correlation of PC1 with genomically-determined tumor fraction was >90% regardless of window sizes (**Supplementary fig. S21**).

Thus, to preserve more detailed methylation information, and to guarantee successful execution in a reasonable amount of time, the setting of 100bps segments with 50 bps sliding window was applied for the rest of our analysis. The setting in our study does not represent the overall best solution, but we presented a framework that is the most suitable for the samples we analysed.

3. Analysis of low passage whole genome bisulfite sequencing (LP-WGBS) data

Reads from LP-WGBS were processed as high coverage NGS. To calculate PC1 values derived from LP-WGBS, we used the default segmentation length of 100 bps and calculated the methylation ration of each segment based on formula (I). To maximize the available information obtained from our data, we imputed methylation data from higher coverage bisulfite data based regularised iterative PCA algorithm (8) (missMDA R package (v1.13)), and projected on the PCA model as described above. The regularisation process with random initialisation can also circumvent the over-fitting problem, which might reduce the generalization capabilities of our findings.

4. Low-passage whole genome sequencing

Low-passage whole genome sequencing (LP-WGS) on both bisulfite-treated and untreated plasma DNA was performed with a target 1x coverage. For each sample, reads from LP-WGS on untreated plasma DNA were aligned to the hg19 using BWA-MEM version 0.7.12-r1039 and de-duplicated using Picard tools v2.1.0. The human genome was then divided into non-overlapping bins of 1 million base pairs, and, for each sample, the de-duplicated reads were counted per bin using HMM Copy (http://compbio.bccrc.ca/software/hmmcopy/) (9). Next, ichorCNA (https://github.com/broadinstitute/ichorCNA) was applied to estimate the tumor content of each sample (10). The algorithm first, removed bins in the centromere regions, with a flanking region of 100,000 base pairs. For all the remaining bins read counts were corrected by GC content and mappability issues. The normalised read counts were then fed into the Hidden Markov model (HMM), which is a probabilistic model assigning each bin into one possible state (hemizygous deletions (HETD, 1 copy), copy neutral (NEUT, 2 copies), copy gain (GAIN, 3 copies), amplification (AMP, 4 copies), and high-level amplification (HLAMP, 5 or more copies). Based on the copy number profile, the model estimated a ploidy and tumor content for every sample. Finally, the algorithm was initiated with ploidy values 2 and 3, and normal fraction, which is 1 minus tumor fraction of 0.0, 0.1, 0.2, 0.3, 0.4, 0.5, 0.6, 0.7, 0.8, 0.9, 0.95. The solution with maximum likelihood among all of these initial combinations was automatically assigned. The CNA status was estimated based on the logR values of each 1Mbp region obtained by the ichorCNA analysis with fixed threshold of 0.5 (GAIN: logR ≥ 0.5, LOSS: logR ≤ 0.5).

5. Methylation Signatures by Principal Component Analysis (PCA)

We applied unscaled PCA using FactoMineR (http://factominer.free.fr) (11). The PCA model comes with the eigenvector, eigenvalues and correlation matrix comprised of correlation coefficient by each segment. We plotted the distribution of the top-K highly correlated segments based on the correlation matrix returned by PCA, and these segments were highly representative of each eigenvector (e.g., principal component 1, or PC1). To identify the optimal value K of highly correlated segments, we tested multiple K values equal to 10, 100, 1,000, and 10,000 and calculate intra-sample variance, and the correlation between median methylation ratio with genomically-determined tumor fraction (**Supplementary Fig S5 and S6**).

6. Gaussian Mixture Model (GMM)

Methylation ratio of ct-MethSig segments derived from LNCaP cell lines, and healthy volunteer plasma were extracted. To estimate the probability density function (pdf), we applied kernel density estimation (kde), assuming a mixture of two Gaussian distributions consistent with the input dataset of normal prostate epithelium (**Fig. 6**). The Gaussian mixture model (see formula II) applies expectation-maximization (EM) to fit the mixtures of Gaussian distributions by an iterative process (12). In our experimentation, the model was executed with maximum iterations of 100 times and 'k-means' method for initialization, and we hypothesized that there were two Gaussian distributions, each of them with its own general covariance. The Gaussian mixture model was subject to cross-validation on random split set of regions over 100 times to prove the robustness of the approach (**Supplementary Fig S22**). The fitted GMM (number of class = 2) was then used to predict ct-MethSig segments of prostate epithelium (PrEC) (13).

Gaussian mixture model: $g_j(x) = \emptyset_{\theta_j}(x); where\ \theta_j = \left(\mu_i, \sigma_j^2\right)$ (II)

7. Analysis of Illumina HumanMethylation450 BeadChip dataset

The microarray processed data were obtained from the Gene Expression Omnibus (14) repository (GSE84043). From the dataset we selected the probes overlapping with MethSig1 segments. The methylation ratio of each segment was obtained considering the median of the β values of the overlapping probes. The tumor fraction estimates by different methods were obtained by the sample information published (15).

8. Statistical Analyses

8.1 Methylation ratio difference with Kruskal-Wallis and Dunn's test

The samples were grouped based on tissue of origin and clinical status (white blood cells, plasma healthy volunteer, plasma baseline and plasma progression). Samples were grouped by ct-MethSig and AR-MethSig, and the median methylation ratio of each 100bp segment was estimated in each group of samples. To keep the analysis consistent, we considered only segments present in all samples (340,467 segments). All the selected segments were split in two groups based on the overlap with the promoter region of known genes (263,262 non-promoter segments, 77,205 promoter segments). The promoter region was defined as 1k base-pair upstream and downstream of the transcription start site (TSS). The significance of the differences among each group was calculated using Kruskal-Wallis test (one-way ANOVA on ranks) as implemented in the R v3.4.0 (https://www.R-project.org (2018)) stats package. After we defined the significance of the differences, we assessed the difference of the methylation ratio across each group using the Dunn's test as implemented in FSA R package v0.8.22 (https://github.com/droglenc/FSA).

8.2 Correlation and association analysis

Correlation analyses of continuous measures were performed using the Pearson correlation method as implemented in the R v3.4.0 stats package. The association analysis between principal components and CNA of each region was performed by grouping the principal component values of each sample based on the CNA observed for the region (LOSS, NEUTRAL and GAIN). The differences in the principal component values distribution among groups was then assessed using the Kruskal-Wallis test (one-way ANOVA on ranks) as implemented in the R v3.4.0 stats package.

8.3 Functional enrichment analysis

Functional enrichment analysis (chemical and genetic perturbations, MSigDB) was executed using the enrich R package (v0.1) based on all the MSigDB main categories (MSigDB database v6.0) (16) with a significance threshold of 0.05 on Benjamini corrected p values.

8.4 Motif enrichment analysis

Motif enrichment analysis was used to identify potential transcriptomic regulators of methylation signatures (MethSig). The start of MethSig top 1000 correlated segments were submitted to find the possible motif binding sequences over-represented as compared to the default background set (17). The pipeline (Pscan-Chip) (17) originally designed for the analysis of chromatin immunoprecipitation followed by next generation sequencing technologies was applied. The program automatically scanned 75 bps preceding and after the 'peak' regions that we submitted with controlled background, and know transcriptional factor binding motifs obtained from JASPAR version 2018. Local enrichment p-value was two-tailed and denoted whether the motif was over-represented in the 150-bp region compared to the genomic regions flanking them. Global enrichment denoted whether the motif binding sequence was over-represented in the region with respect to global background composed of pan-genome putative regulatory regions from various cell lines. We performed the analysis on top highly correlated segments with PC1 or PC3 and other randomly selected regions from our custom, targeted enrichment panel. The result of ar-MethSig was validated by an orthogonal pipeline (18), and the finding was consistent to original approach as described above (data not shown).

References

1. Xi Y, and Li W. BSMAP: whole genome bisulfite sequence MAPping program. *BMC Bioinformatics.* 2009;10:232.
2. Bolger AM, Lohse M, and Usadel B. Trimmomatic: a flexible trimmer for Illumina sequence data. *Bioinformatics.* 2014;30(15):2114-20.
3. Jun G, Wing MK, Abecasis GR, and Kang HM. An efficient and scalable analysis framework for variant extraction and refinement from population-scale DNA sequence data. *Genome Res.* 2015;25(6):918-25.
4. Akalin A, Kormaksson M, Li S, Garrett-Bakelman FE, Figueroa ME, Melnick A, et al. methylKit: a comprehensive R package for the analysis of genome-wide DNA methylation profiles. *Genome Biol.* 2012;13(10):R87.
5. Lehmann-Werman R, Neiman D, Zemmour H, Moss J, Magenheim J, Vaknin-Dembinsky A, et al. Identification of tissue-specific cell death using methylation patterns of circulating DNA. *Proc Natl Acad Sci U S A.* 2016;113(13):E1826-34.
6. Guo S, Diep D, Plongthongkum N, Fung HL, Zhang K, and Zhang K. Identification of methylation haplotype blocks aids in deconvolution of heterogeneous tissue samples and tumor tissue-of-origin mapping from plasma DNA. *Nat Genet.* 2017;49(4):635-42.
7. Sun K, Jiang P, Chan KC, Wong J, Cheng YK, Liang RH, et al. Plasma DNA tissue mapping by genome-wide methylation sequencing for noninvasive prenatal, cancer, and transplantation assessments. *Proc Natl Acad Sci U S A.* 2015;112(40):E5503-12.
8. Josse J, and Husson F. missMDA: A Package for Handling Missing Values in Multivariate Data Analysis. *2016.* 2016;70(1):31.
9. Ha G, Roth A, Lai D, Bashashati A, Ding J, Goya R, et al. Integrative analysis of genome-wide loss of heterozygosity and monoallelic expression at nucleotide resolution reveals disrupted pathways in triple-negative breast cancer. *Genome Res.* 2012;22(10):1995-2007.
10. Adalsteinsson VA, Ha G, Freeman SS, Choudhury AD, Stover DG, Parsons HA, et al. Scalable whole-exome sequencing of cell-free DNA reveals high concordance with metastatic tumors. *Nat Commun.* 2017;8(1):1324.
11. Lê S, Josse J, and Husson F. FactoMineR: An R Package for Multivariate Analysis. *2008.* 2008;25(1):18.
12. Pedregosa F, Ga, #235, Varoquaux l, Gramfort A, Michel V, et al. Scikit-learn: Machine Learning in Python. *J Mach Learn Res.* 2011;12:2825-30.
13. Pidsley R, Zotenko E, Peters TJ, Lawrence MG, Risbridger GP, Molloy P, et al. Critical evaluation of the Illumina MethylationEPIC BeadChip microarray for whole-genome DNA methylation profiling. *Genome Biol.* 2016;17(1):208.
14. Edgar R, Domrachev M, and Lash AE. Gene Expression Omnibus: NCBI gene expression and hybridization array data repository. *Nucleic Acids Res.* 2002;30(1):207-10.
15. Fraser M, Sabelnykova VY, Yamaguchi TN, Heisler LE, Livingstone J, Huang V, et al. Genomic hallmarks of localized, non-indolent prostate cancer. *Nature.* 2017;541(7637):359-64.
16. Liberzon A, Birger C, Thorvaldsdottir H, Ghandi M, Mesirov JP, and Tamayo P. The Molecular Signatures Database (MSigDB) hallmark gene set collection. *Cell Syst.* 2015;1(6):417-25.

17. Zambelli F, Pesole G, and Pavesi G. PscanChIP: Finding over-represented transcription factor-binding site motifs and their correlations in sequences from ChIP-Seq experiments. *Nucleic Acids Res.* 2013;41(Web Server issue):W535-43.
18. Heinz S, Benner C, Spann N, Bertolino E, Lin YC, Laslo P, et al. Simple combinations of lineage-determining transcription factors prime cis-regulatory elements required for macrophage and B cell identities. *Mol Cell.* 2010;38(4):576-89.

Supplementary figures

figure S1. Box plot showing coverage distribution in target regions by bisulfite high-coverage next-generation sequencing (NGS) in plasma samples.

figure S2. Box plot showing methylation ratio distribution for baseline (A) and progression (B) plasma samples and white blood cells (C) presented separately.

figure S3. The genomic annotation based on location of methylation segments in the custom targeted panel and in segments covered >10X in 19 baseline samples.

figure S4. Bland-Altman plot showing agreement for tumor fraction estimation by genomically-determined tumor fraction and on LP-WGBS.

figure S5. Correlation of median methylation ratio of selected segments with genomically-determined tumor fraction. Y-axis shows the correlation value and the X-axis denotes the number of top correlated segments.

figure S6. Standard deviation of methylation ratios of selected segments. Y-axis shows the standard deviation and the X-axis denotes the number of top correlated segments.

figure S7. Methylation ratios of *GSTP1*, *APC*, and *RASSF1A* across different tissue types—healthy volunteer plasma, white blood cells, CRPC plasma samples, LNCaP cell line.

figure S8. ct-MethSig segment methylation ratio derived from mCRPC tissues lined by tumor fraction

figure S9. Correlation between HSPC tissue tumor fraction estimation by ct-MethSig and molecularly-defined tumor fraction.

figure S10. Permutation test on genes overlapping with ct-MethSig; the red dot represents the gene enrichment test (GSEA) *P* value in genes overlapping with ct-MethSig and the box represents *P* values of the permutation test with 1000-time iteration.

figure S11. Methylation ratio distribution of circulating normal prostate specific or prostate cancer specific component in localized prostate cancer from TCGA

figure S12. Median methylation ratio of 993 MethSig3 segments positively correlated with PC3 values across different sample types—plasma, white blood cells, cell lines (LNCaP, LNCaP95, VCaP), CASCADE tumor (mCRPC biopsy) are plotted against the median methylation ratio of top correlated segments with ct-MethSig.

figure S13. Copy number alteration plots from LP-WGS on plasma DNA with and without bisulfite treatment

figure S14. Prevalence of gain and loss events lined by chromosome position extracted from LP-WGBS on mCRPC plasma samples.

figure S15. Analysis of copy number profiles on low-pass whole genome bisulfite sequencing. Matrix shows gains (red) and losses (blue) ordered by chromosomal position (columns) for individual patient samples (one per row) ordered by tumor purity. Bar chart on the left shows tumor fraction per sample. Bar chart on the right shows the number of gain (red) or loss (blue) events per sample.

figure S16. Manhattan plot showing the level of significance of the association between PC1 value distribution and copy number alterations ordered by chromosome position. The segment containing AR is highlighted as green dot (not significant, P = 0.18).

figure S17. Manhattan plot showing the level of significance of the association between PC3 value distribution and copy number alterations ordered by chromosome position. The segment containing *AR* is highlighted as a green dot (*P* = 0.018, Kruskal-Wallis test).

figure S18. Bland-Altman plot showing agreement between targeted methylation NGS and LP-WGBS on AR-MethSig median methylation ratio

figure S19. Percentage of data to drop on different window sizes (10bps, 100bps, 1000bps, 10000bps)

figure S20. Distribution of methylation ratio by different segment size (10 bps, 100 bps, 1,000 bps, 10,000 bps)

figure S21. Correlation of genomically-determined tumor fraction and PC1 values derived from PCA on different window sizes (10bps, 100bps, 1000bps, 10000bps)

figure S22. Performance of Gaussian Mixture Model (k-fold cross-validation, k=100)

Supplementary Tables

Table S1. Patient plasma sample characteristics.

Table S2. Targeted methylome sequencing matrix (total reads, mapped reads, % mapped reads)

Table S3. LP-WGBS matrix (total reads, mapped reads, % mapped reads)

Table S4. CASCADE patient and sample characteristics.

Table S5. Genes overlapping with AR-MethSig

Table S6. Functional enrichment of top correlated segments with principal component three

Table S7. AR-MethSig motif enrichment analysis.

Table S8. Contingency tables showing ct-MethSig and AR-MethSig segments in copy number aberrant regions.

figure S1.

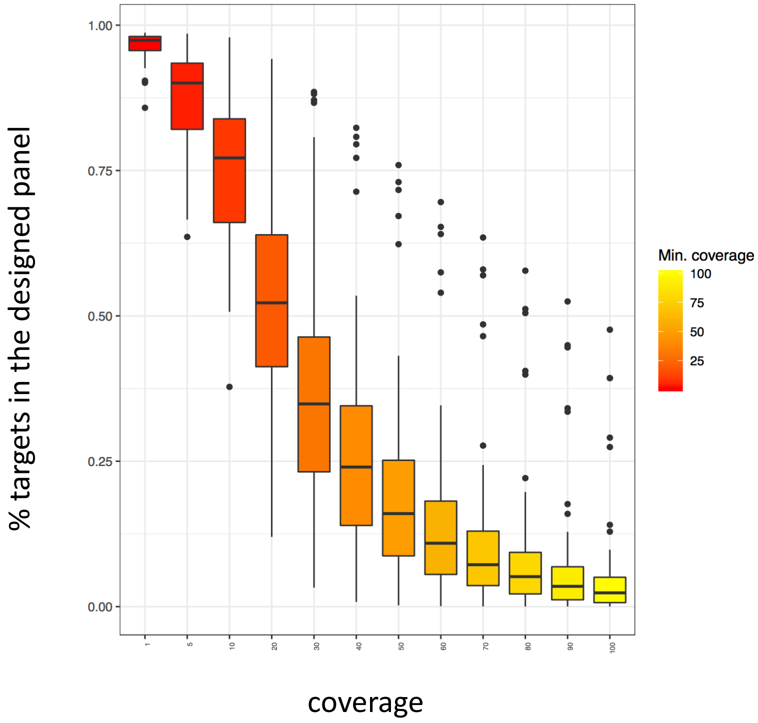
% targets in the designed panel
1.00
0.75
0.50
0.25
0.00
1
5
10
20
30
40
50
60
70
80
90
100
coverage
Min. coverage
100
75
50
25

figure S2.

(A)

(B)

(C)

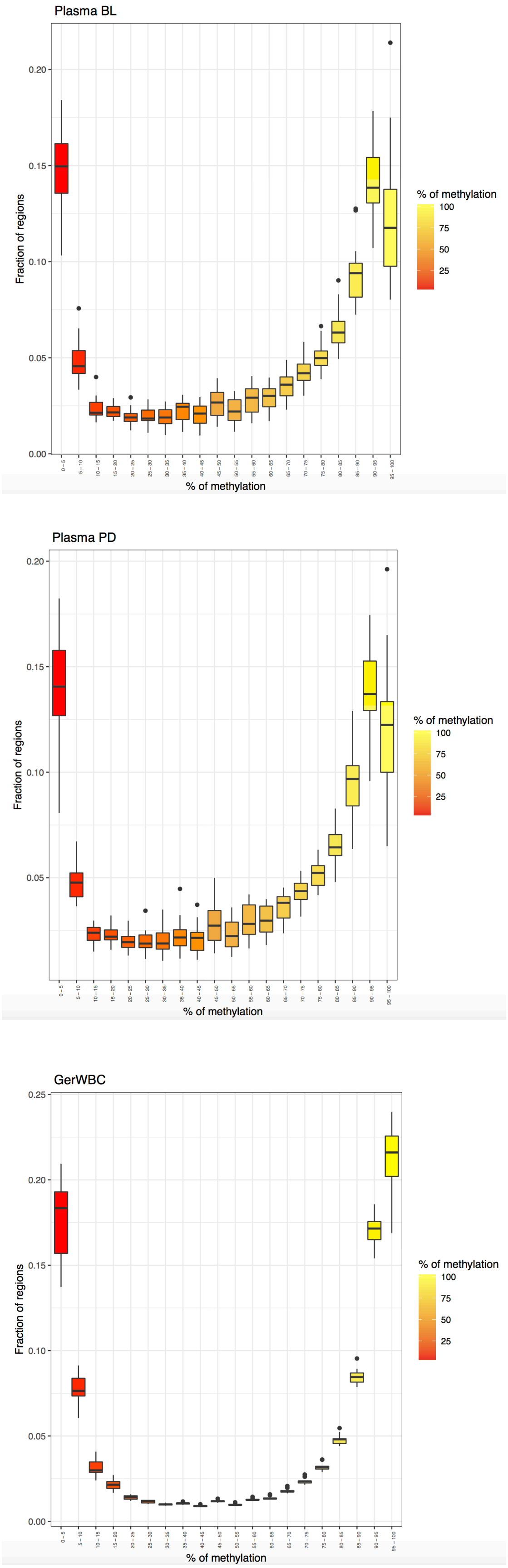

figure S3

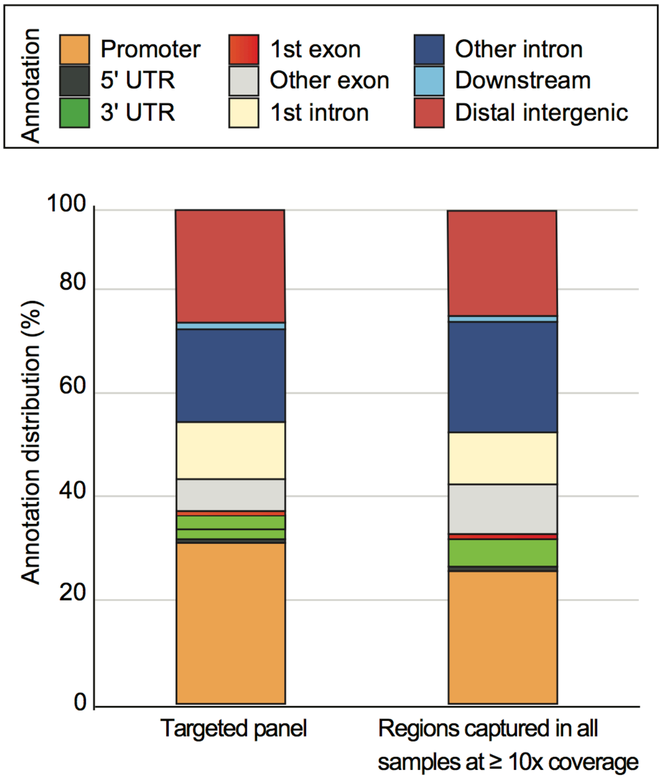
Annotation
Promoter
5' UTR
3' UTR
1st exon
Other exon
1st intron
Other intron
Downstream
Distal intergenic
Annotation distribution (%)
100
80
60
40
20
0
Targeted panel
Regions captured in all samples at ≥ 10x coverage

figure S4.

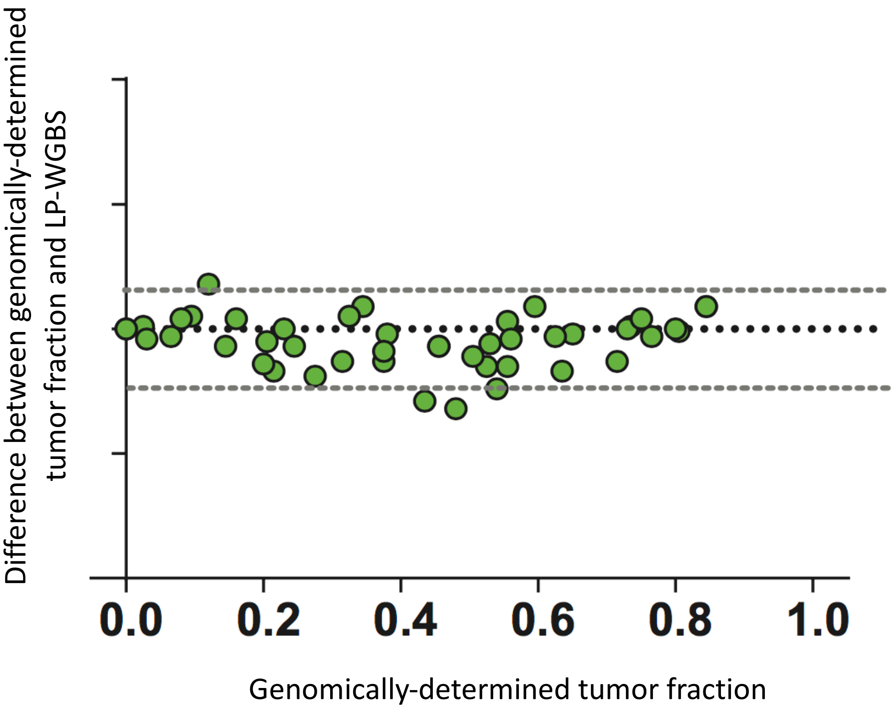

**Bias**	**-0.0480**
**SD of bias**	**0.1028**
**95% Limits of Agreement**	
**From**	**-0.2495**
**To**	**0.1534**

figure S5.

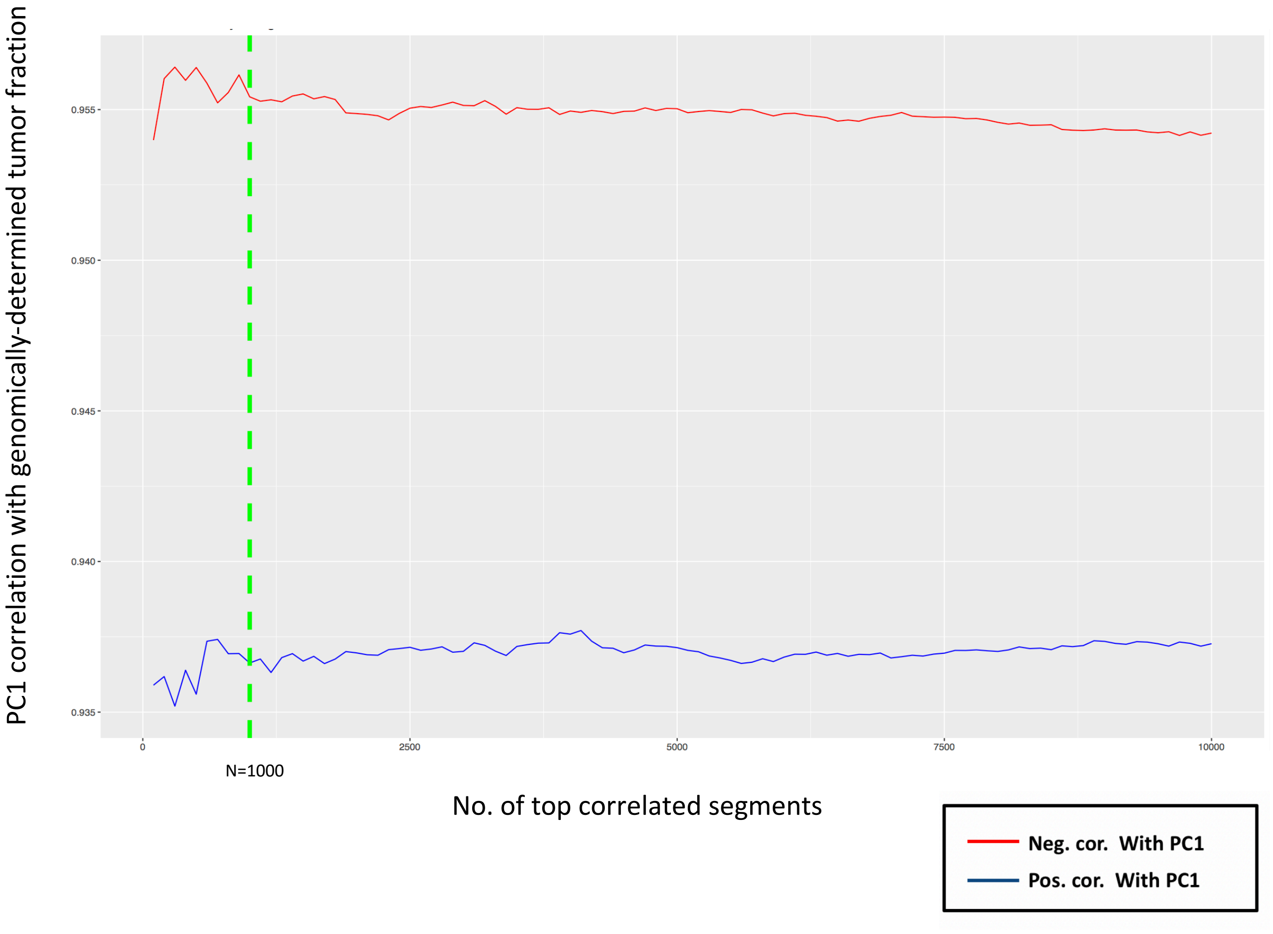
PC1 correlation with genomically-determined tumor fraction
0.955
0.950
0.945
0.940
0.935
0
2500
5000
7500
10000
N=1000
No. of top correlated segments
Neg. cor. With PC1
Pos. cor. With PC1

figure S6.

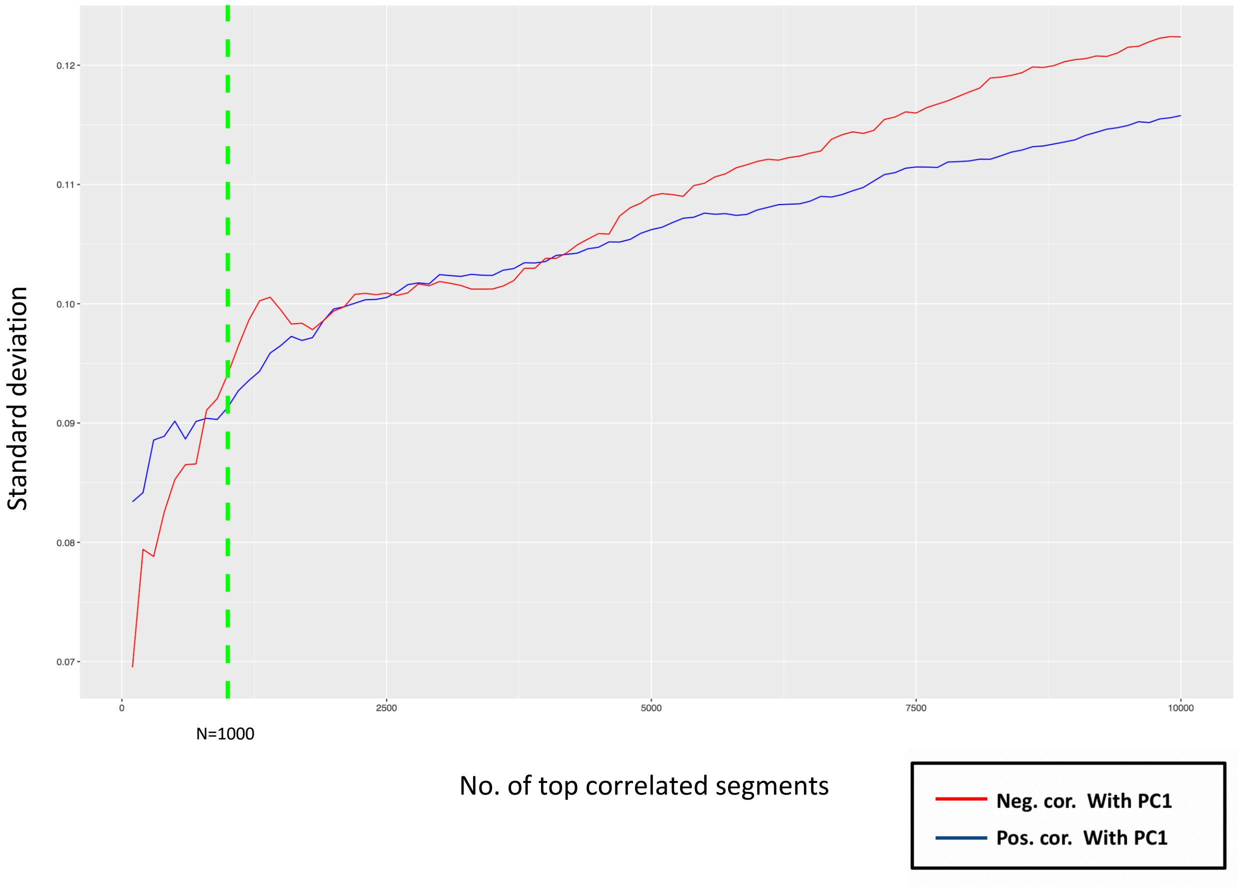
Standard deviation
0.12
0.11
0.10
0.09
0.08
0.07
0
2500
5000
7500
10000
N=1000
No. of top correlated segments
Neg. cor. With PC1
Pos. cor. With PC1

figure S7

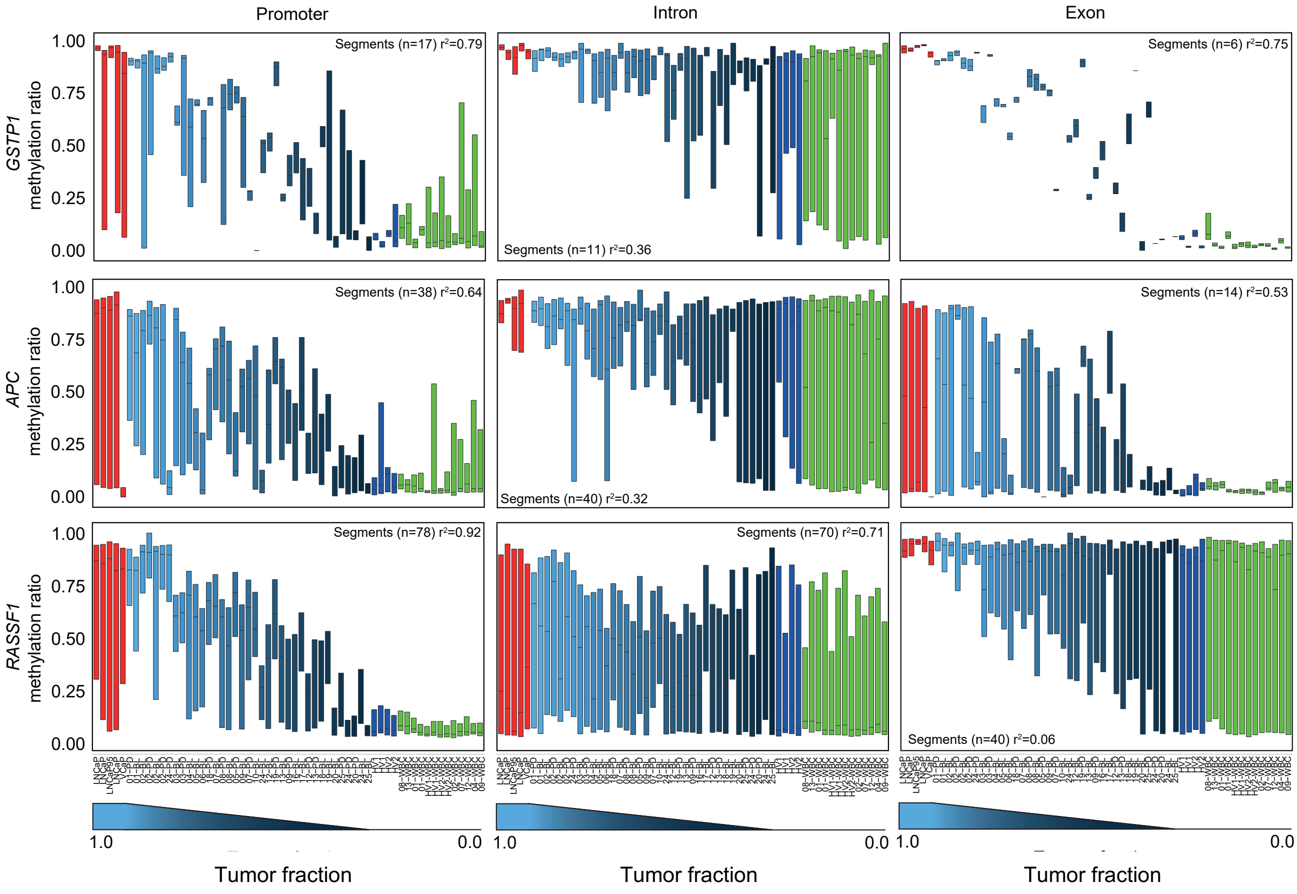

Promoter
Intron
Exon
GSTP1 methylation ratio
APC methylation ratio
RASSF1 methylation ratio
1.00
0.75
0.50
0.25
0.00
Segments (n=17) r²=0.79
Segments (n=11) r²=0.36
Segments (n=6) r²=0.75
Segments (n=38) r²=0.64
Segments (n=40) r²=0.32
Segments (n=14) r²=0.53
Segments (n=78) r²=0.92
Segments (n=70) r²=0.71
Segments (n=40) r²=0.06
1.0
0.0
Tumor fraction

figure S8.

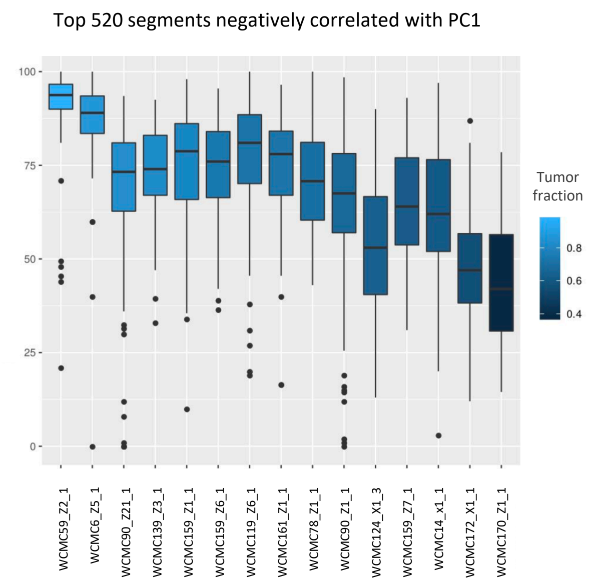
Top 520 segments negatively correlated with PC1
100
75
50
25
0
WCMC59_Z2_1
WCMC6_Z5_1
WCMC90_Z21_1
WCMC139_Z3_1
WCMC159_Z1_1
WCMC159_Z6_1
WCMC119_Z6_1
WCMC161_Z1_1
WCMC78_Z1_1
WCMC90_Z1_1
WCMC124_X1_3
WCMC159_Z7_1
WCMC14_x1_1
WCMC172_X1_1
WCMC170_Z1_1
Tumor fraction
0.8
0.6
0.4

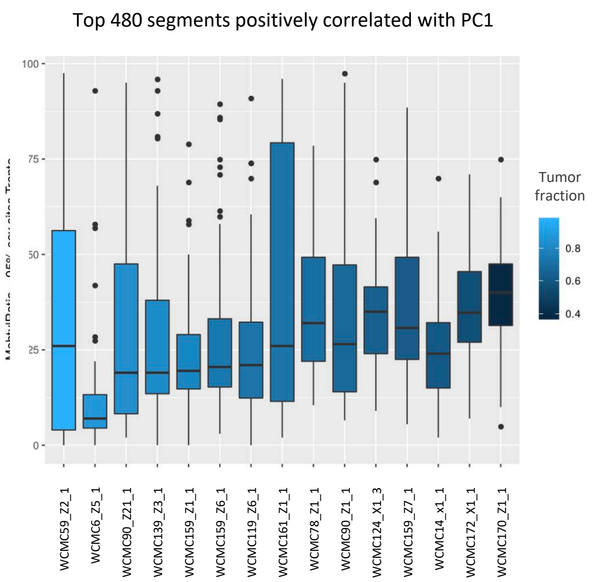
Top 480 segments positively correlated with PC1
100
75
50
25
0
WCMC59_Z2_1
WCMC6_Z5_1
WCMC90_Z21_1
WCMC139_Z3_1
WCMC159_Z1_1
WCMC159_Z6_1
WCMC119_Z6_1
WCMC161_Z1_1
WCMC78_Z1_1
WCMC90_Z1_1
WCMC124_X1_3
WCMC159_Z7_1
WCMC14_x1_1
WCMC172_X1_1
WCMC170_Z1_1
Tumor fraction
0.8
0.6
0.4

figure S9.

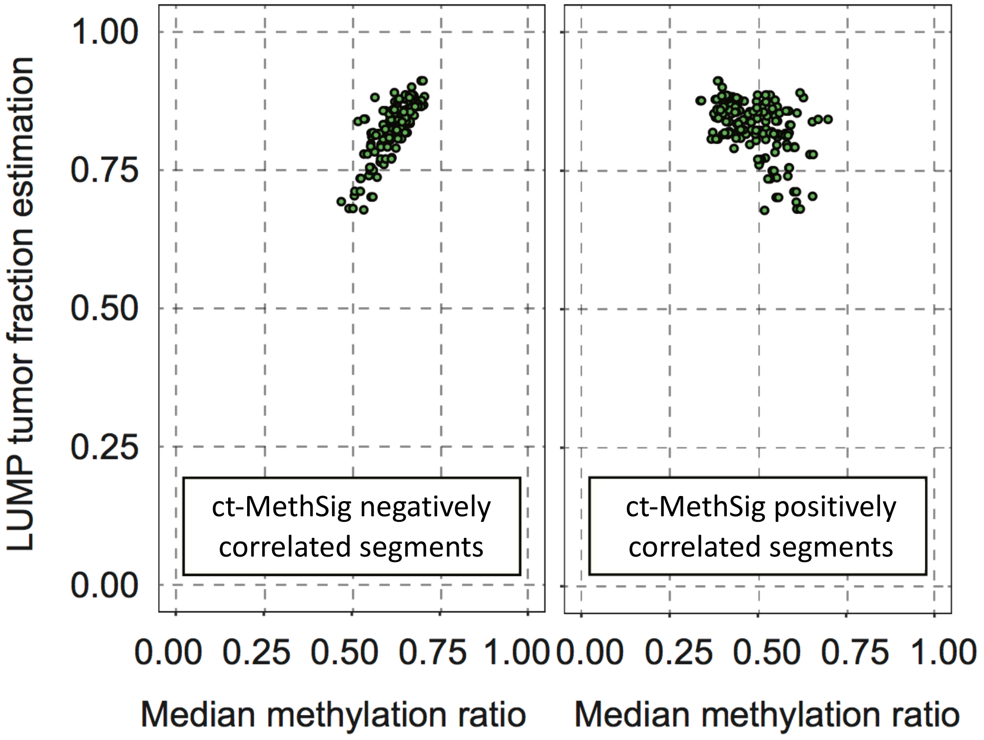

LUMP tumor fraction estimation
1.00
0.75
0.50
0.25
0.00
ct-MethSig negatively correlated segments
ct-MethSig positively correlated segments
0.00 0.25 0.50 0.75 1.00
Median methylation ratio
0.00 0.25 0.50 0.75 1.00
Median methylation ratio

figure S10.

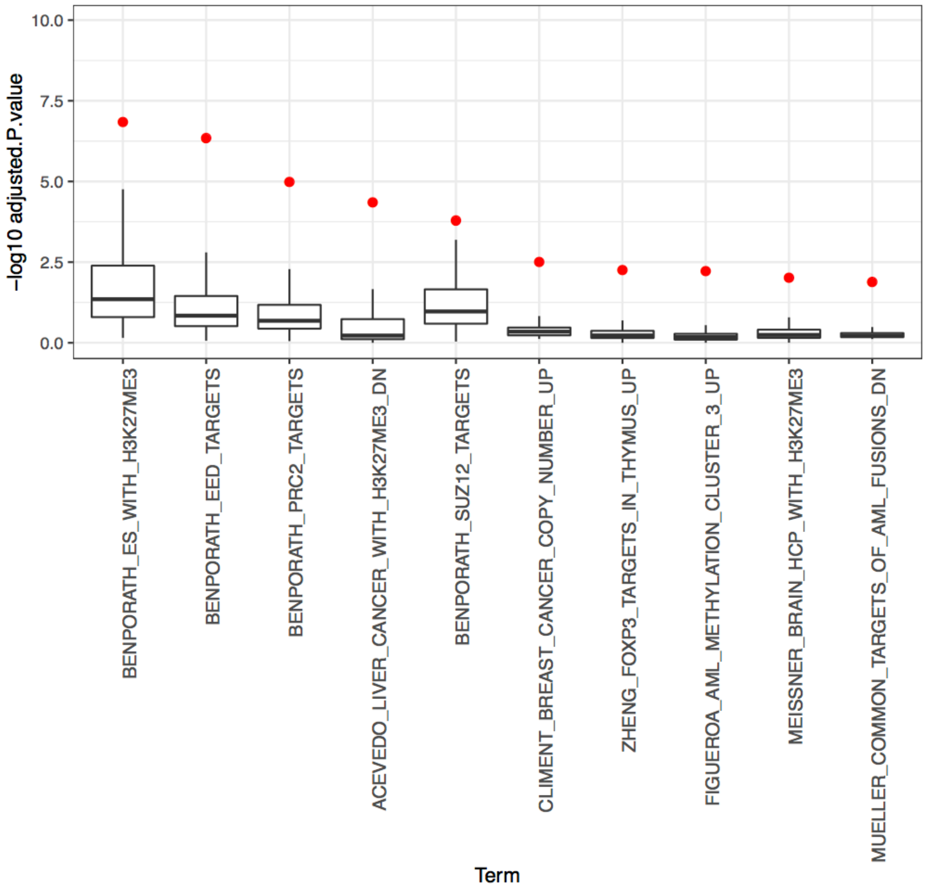

10.0
7.5
5.0
2.5
0.0
−log10 adjusted.P.value
BENPORATH_ES_WITH_H3K27ME3
BENPORATH_EED_TARGETS
BENPORATH_PRC2_TARGETS
ACEVEDO_LIVER_CANCER_WITH_H3K27ME3_DN
BENPORATH_SUZ12_TARGETS
CLIMENT_BREAST_CANCER_COPY_NUMBER_UP
ZHENG_FOXP3_TARGETS_IN_THYMUS_UP
FIGUEROA_AML_METHYLATION_CLUSTER_3_UP
MEISSNER_BRAIN_HCP_WITH_H3K27ME3
MUELLER_COMMON_TARGETS_OF_AML_FUSIONS_DN
Term

figure S11

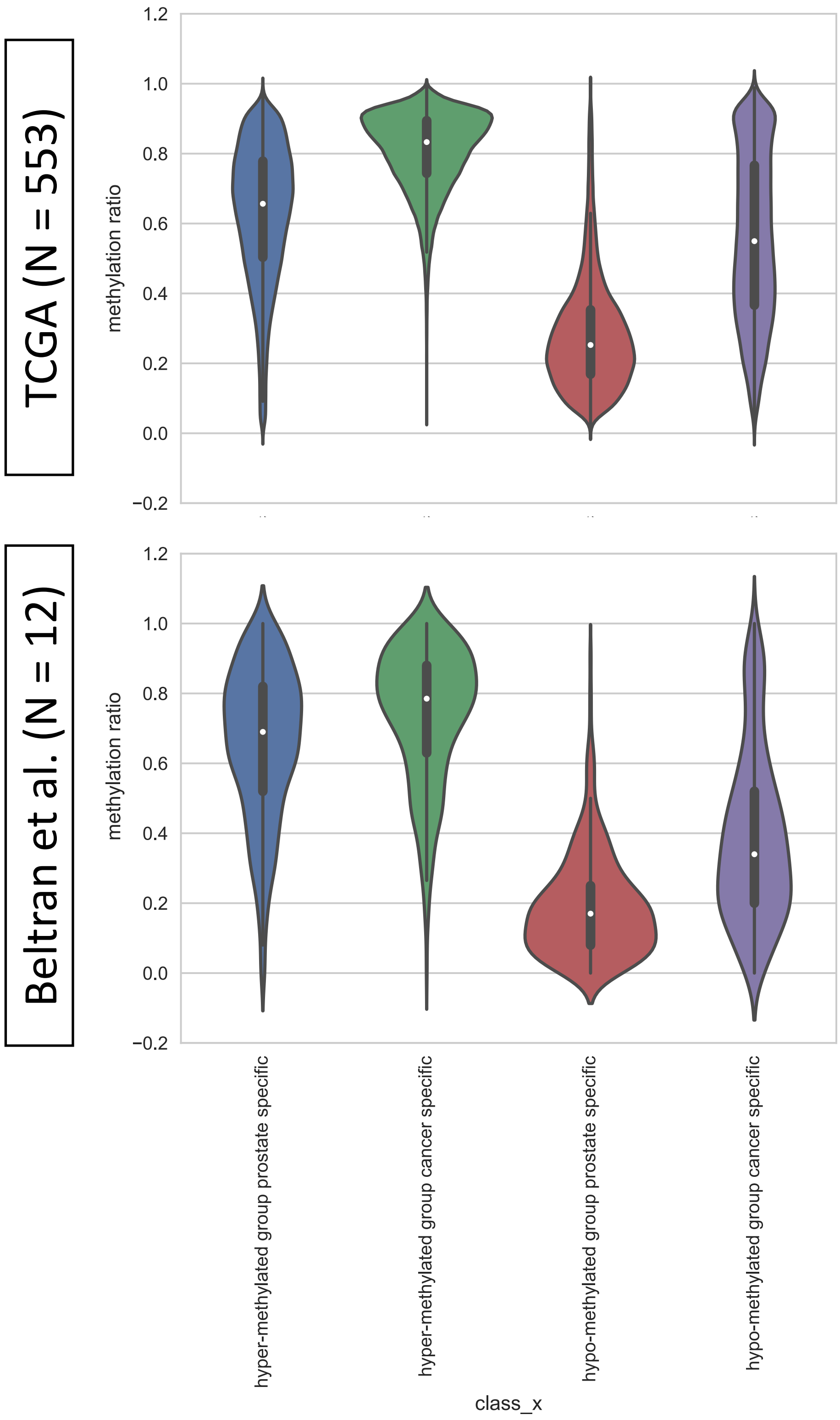
TCGA (N = 553)
Beltran et al. (N = 12)
methylation ratio
1.2
1.0
0.8
0.6
0.4
0.2
0.0
−0.2
hyper-methylated group prostate specific
hyper-methylated group cancer specific
hypo-methylated group prostate specific
hypo-methylated group cancer specific
class_x

figure S12

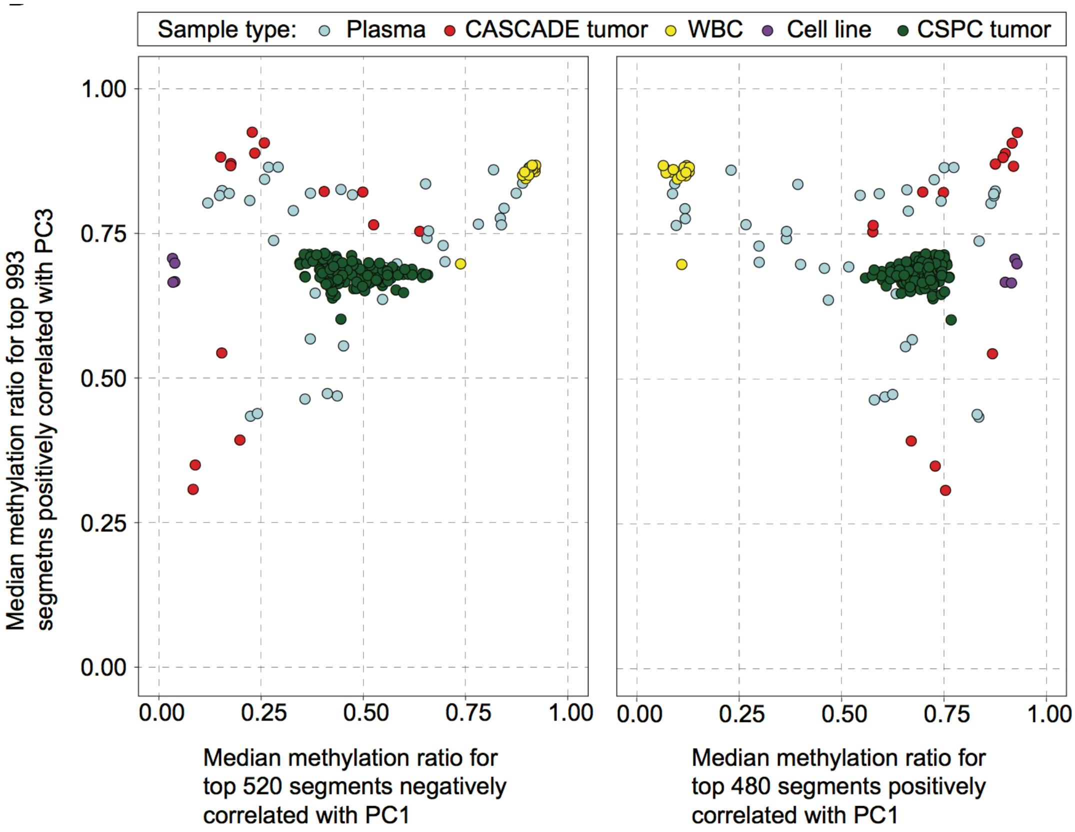

Sample type: Plasma CASCADE tumor WBC Cell line CSPC tumor
Median methylation ratio for top 993 segmetns positively correlated with PC3
1.00
0.75
0.50
0.25
0.00
0.00 0.25 0.50 0.75 1.00
0.00 0.25 0.50 0.75 1.00
Median methylation ratio for top 520 segments negatively correlated with PC1
Median methylation ratio for top 480 segments positively correlated with PC1

figure S13

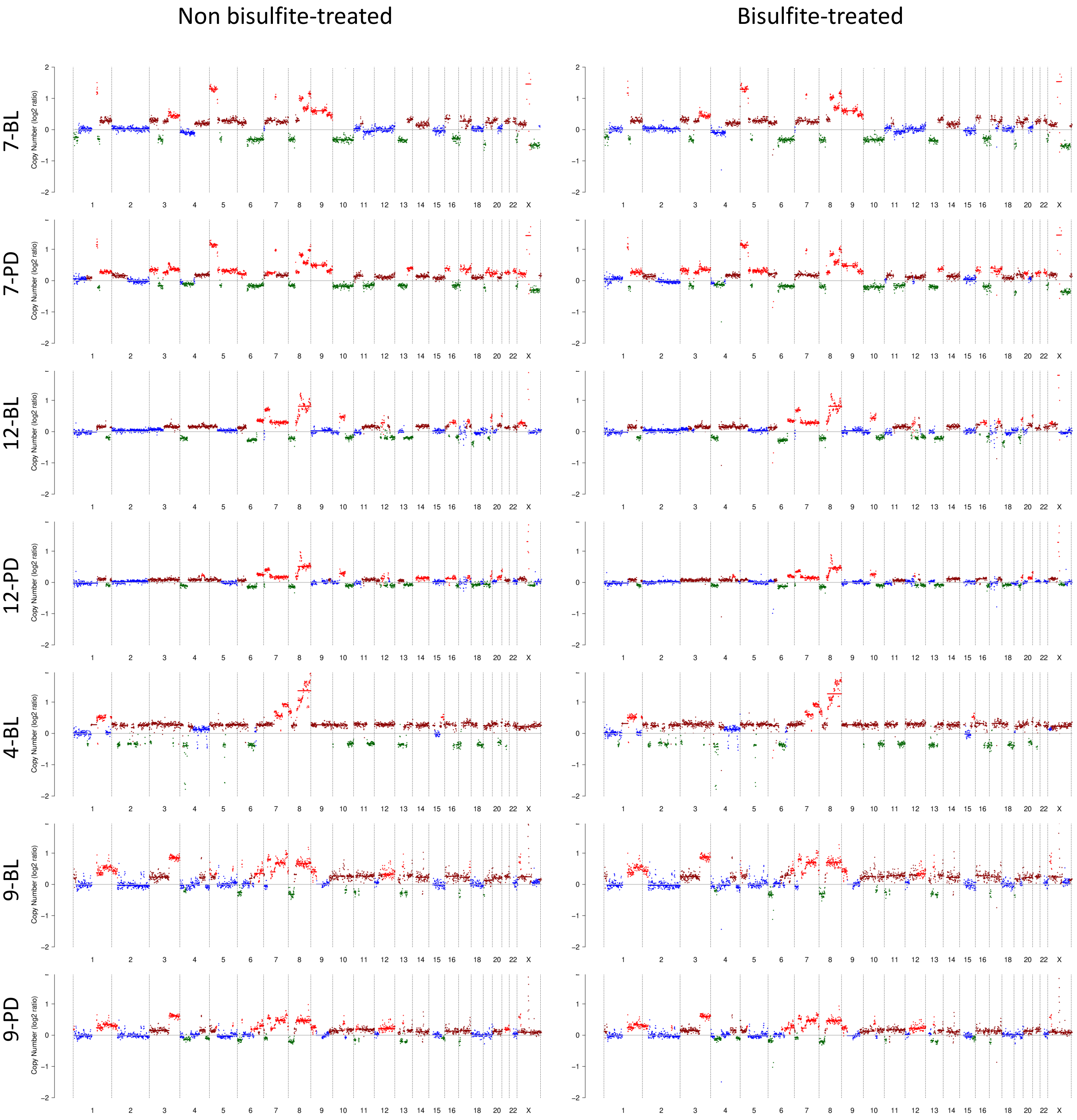

Non bisulfite-treated
Bisulfite-treated
7-BL
7-PD
12-BL
12-PD
4-BL
9-BL
9-PD
Copy Number (log2 ratio)
1 2 3 4 5 6 7 8 9 10 11 12 13 14 15 16 18 20 22 X

figure S14.

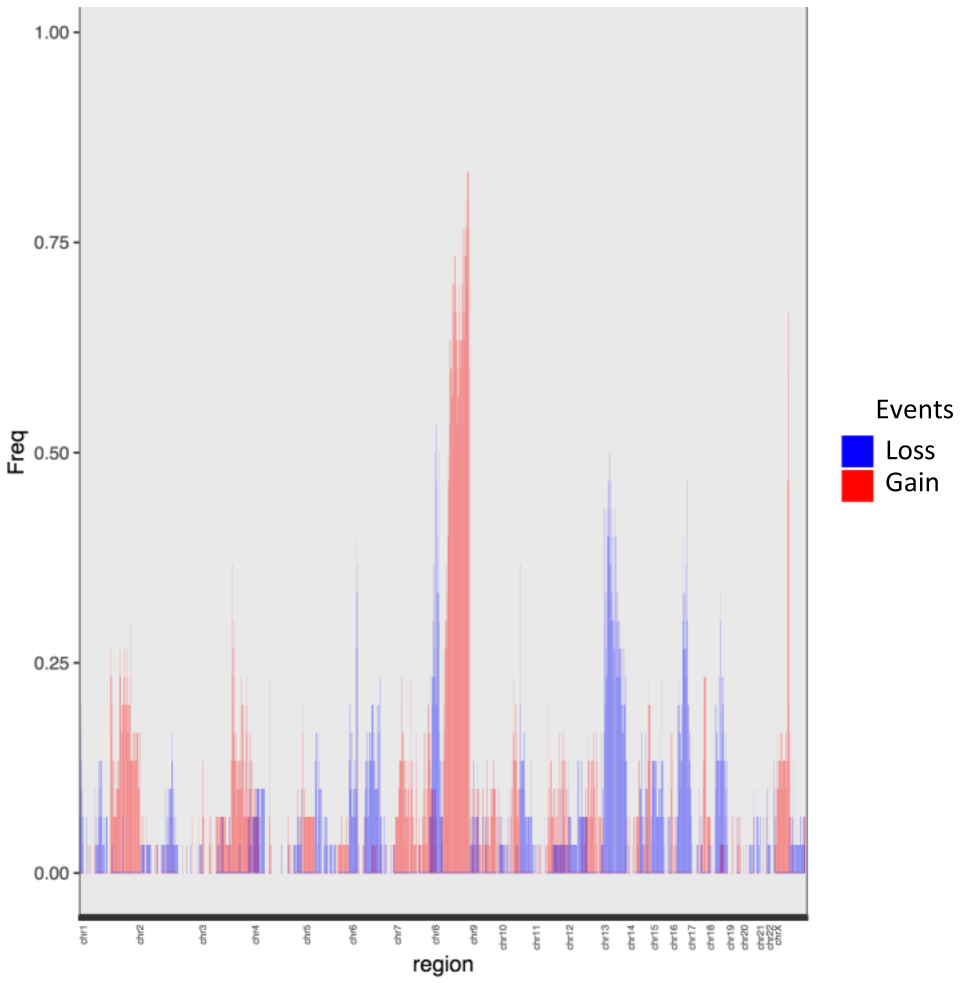

1.00
0.75
0.50
0.25
0.00
Freq
chr1
chr2
chr3
chr4
chr5
chr6
chr7
chr8
chr9
chr10
chr11
chr12
chr13
chr14
chr15
chr16
chr17
chr18
chr19
chr20
chr21
chr22
chrX
region
Events
Loss
Gain

figure S15.

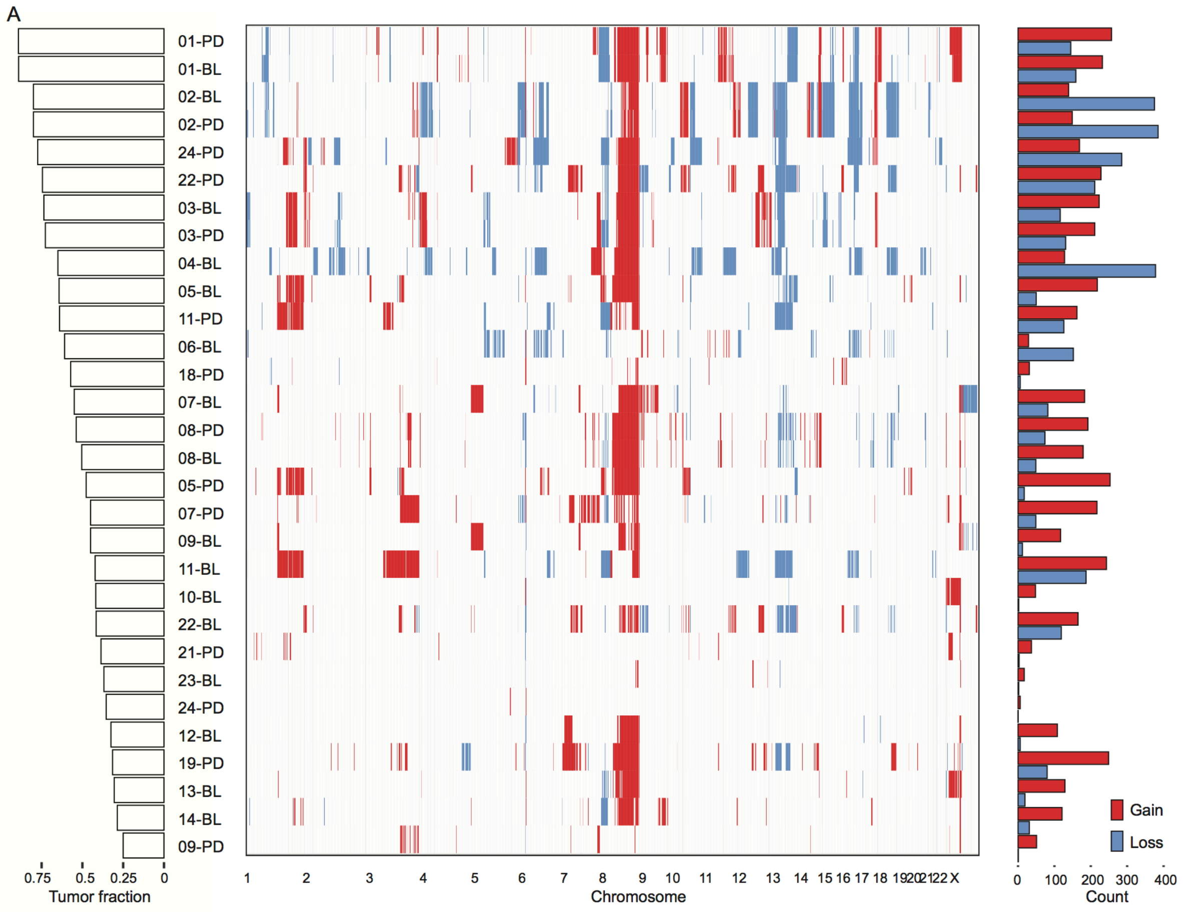
A
01-PD
01-BL
02-BL
02-PD
24-PD
22-PD
03-BL
03-PD
04-BL
05-BL
11-PD
06-BL
18-PD
07-BL
08-PD
08-BL
05-PD
07-PD
09-BL
11-BL
10-BL
22-BL
21-PD
23-BL
24-PD
12-BL
19-PD
13-BL
14-BL
09-PD
0.75 0.5 0.25 0
Tumor fraction
1 2 3 4 5 6 7 8 9 10 11 12 13 14 15 16 17 18 19 20 21 22 X
Chromosome
0 100 200 300 400
Count
Gain
Loss

figure S16.

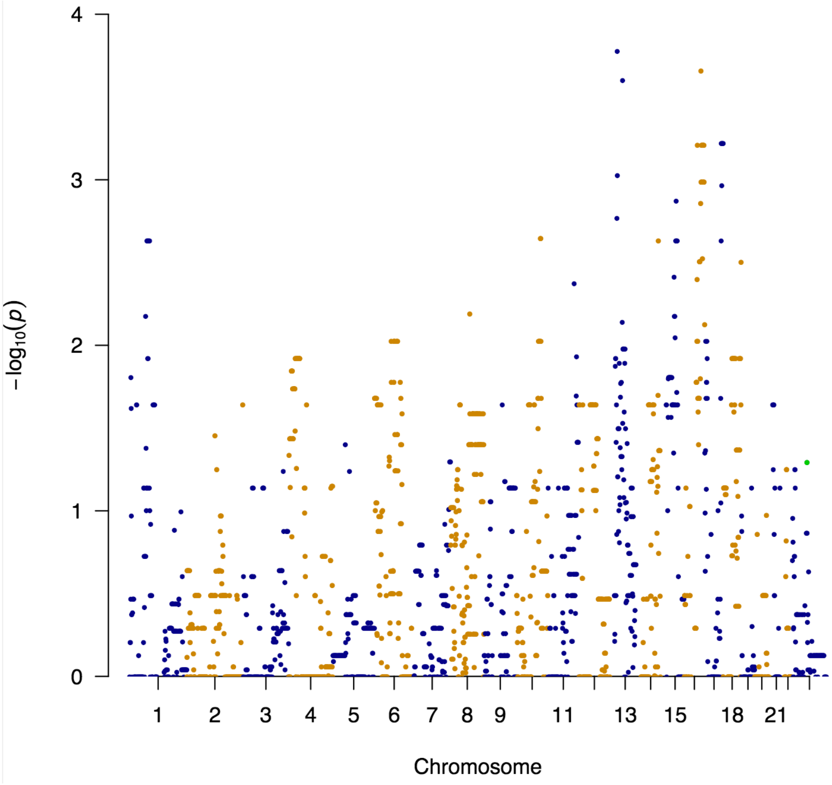

figure S17.

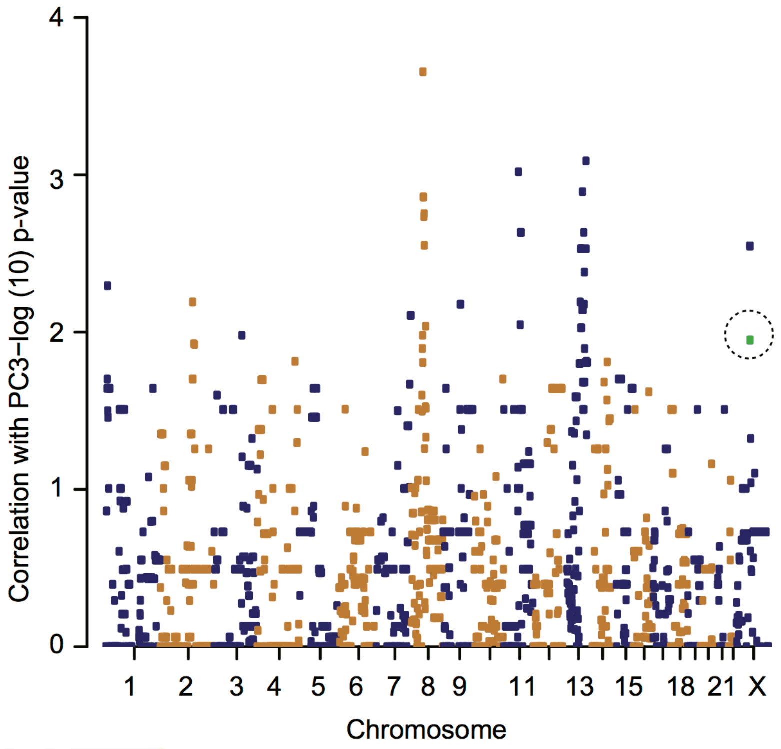

Correlation with PC3-log (10) p-value
4
3
2
1
0
1
2
3
4
5
6
7
8
9
11
13
15
18
21
X
Chromosome

figure S18.

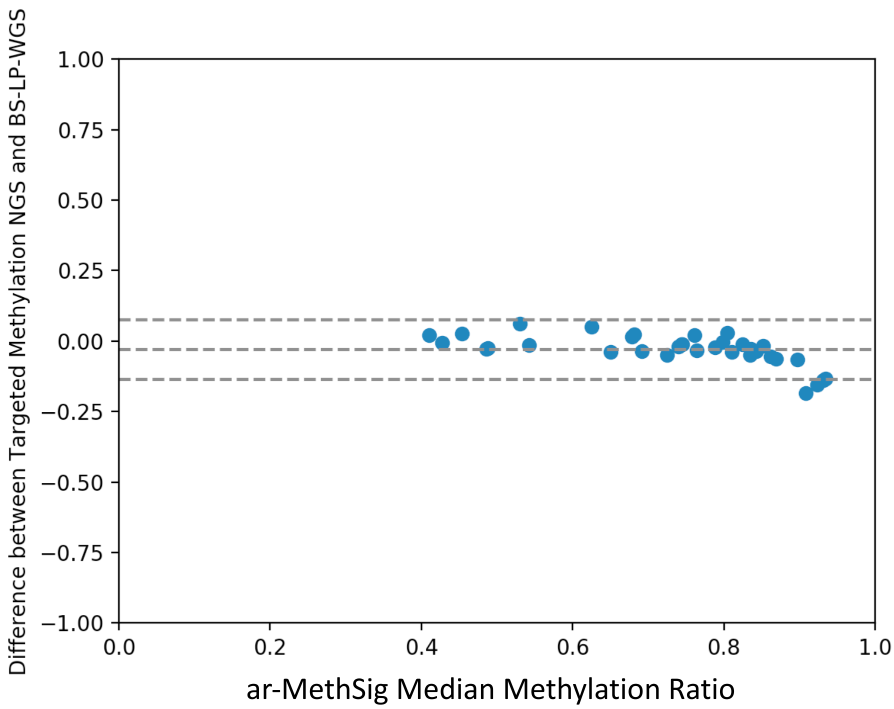

**Bias**	**0.029**
**SD of bias**	**0.054**
**95% Limits of Agreement**	
**From**	**- 0.136**
**To**	**0.076**

figure S19.

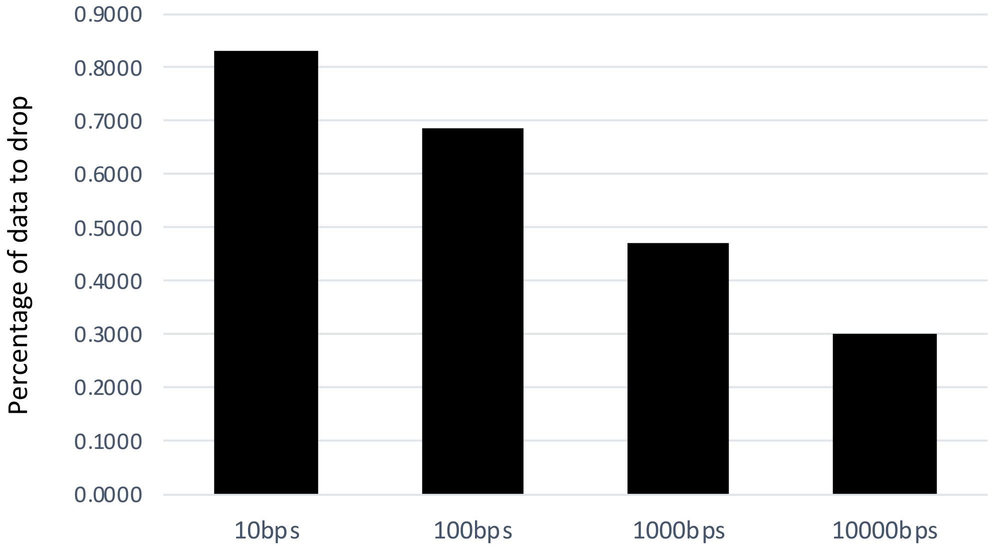
Percentage of data to drop
0.9000
0.8000
0.7000
0.6000
0.5000
0.4000
0.3000
0.2000
0.1000
0.0000
10bps
100bps
1000bps
10000bps

figure S20.

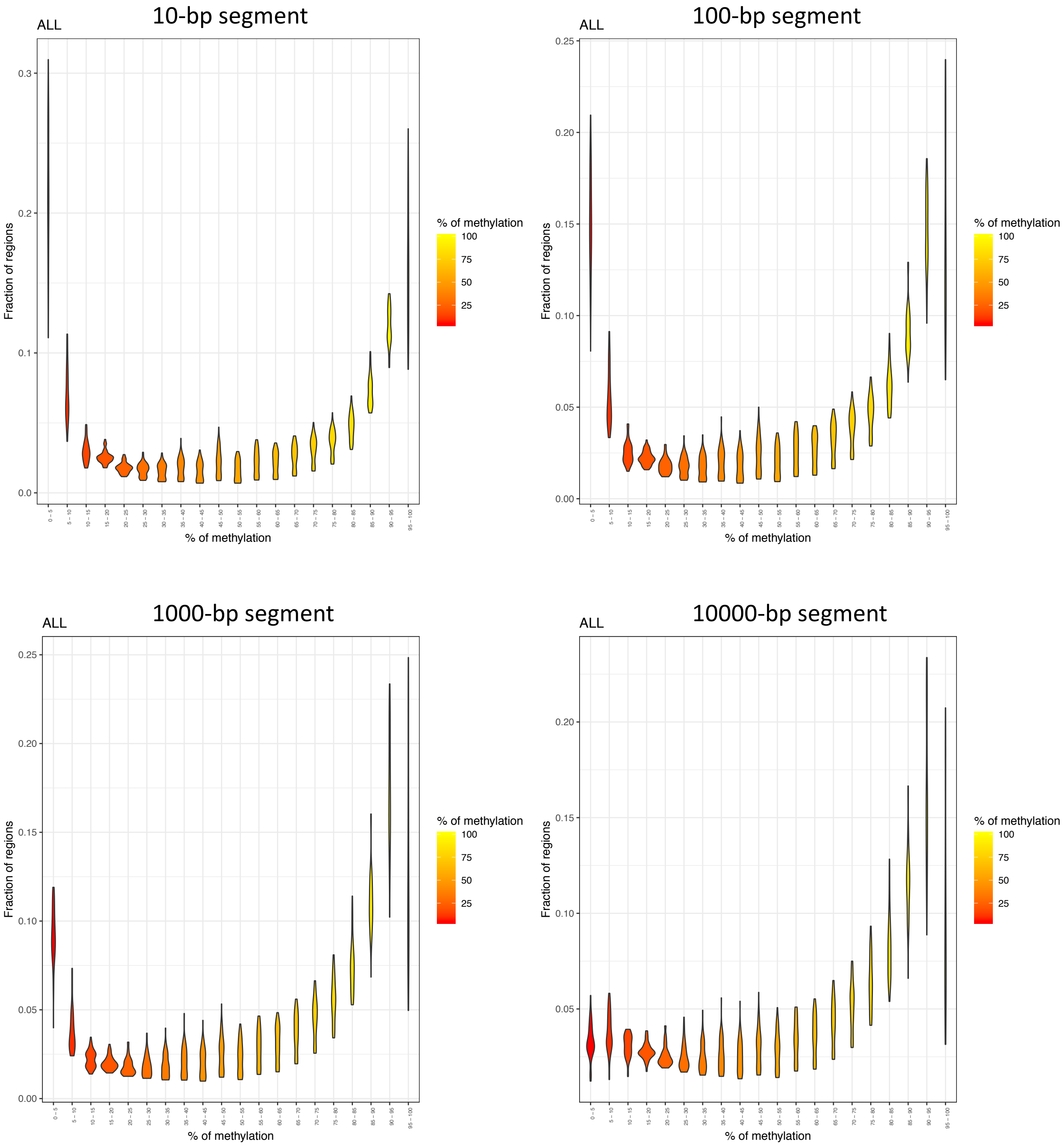
ALL
10-bp segment
100-bp segment
1000-bp segment
10000-bp segment
Fraction of regions
% of methylation
% of methylation
100
75
50
25

figure S21.

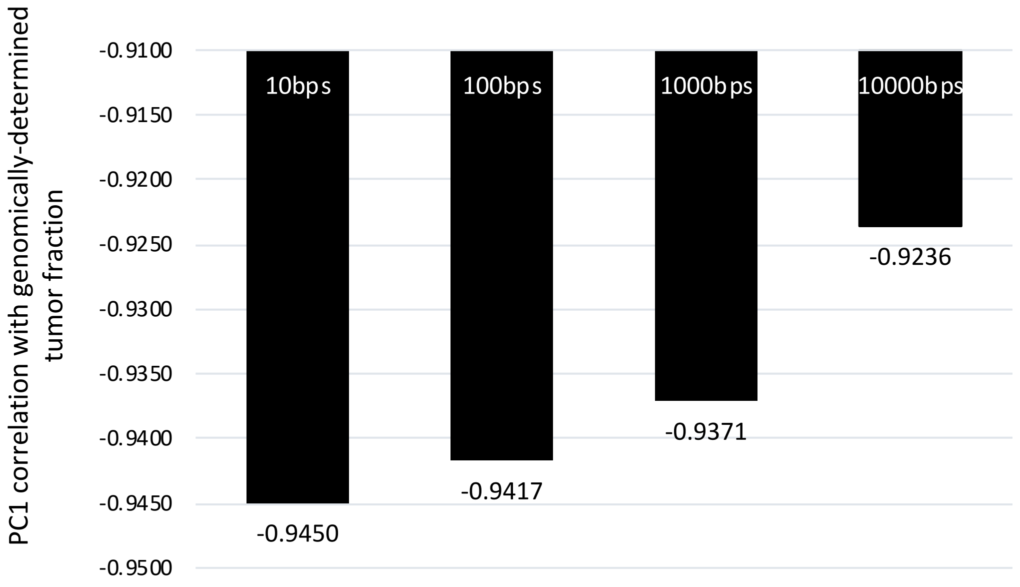
PC1 correlation with genomically-determined tumor fraction
-0.9100
-0.9150
-0.9200
-0.9250
-0.9300
-0.9350
-0.9400
-0.9450
-0.9500
10bps
100bps
1000bps
10000bps
-0.9450
-0.9417
-0.9371
-0.9236

figure S22

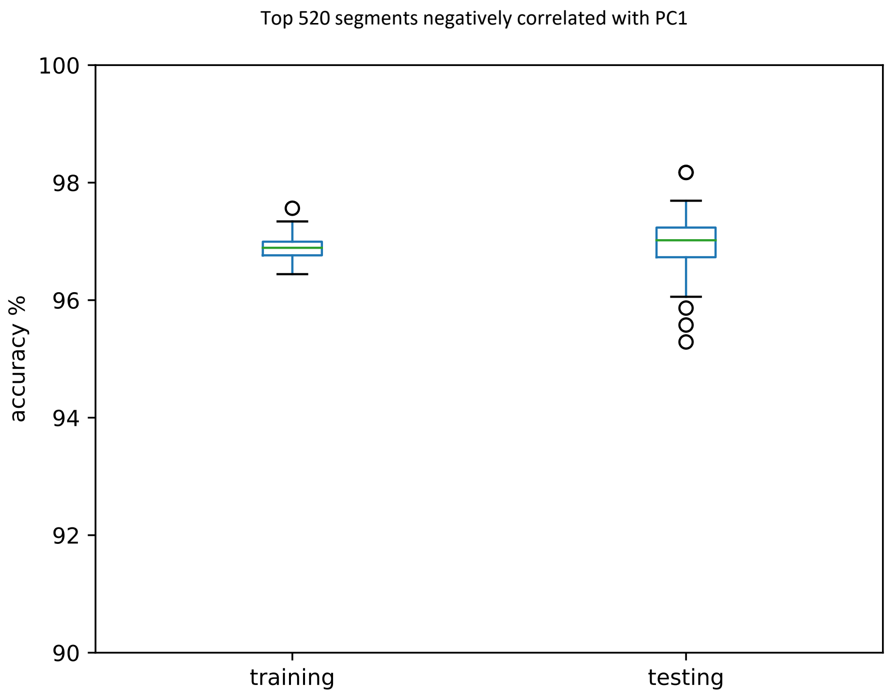
Top 520 segments negatively correlated with PC1
100
98
96
94
92
90
accuracy %
training
testing

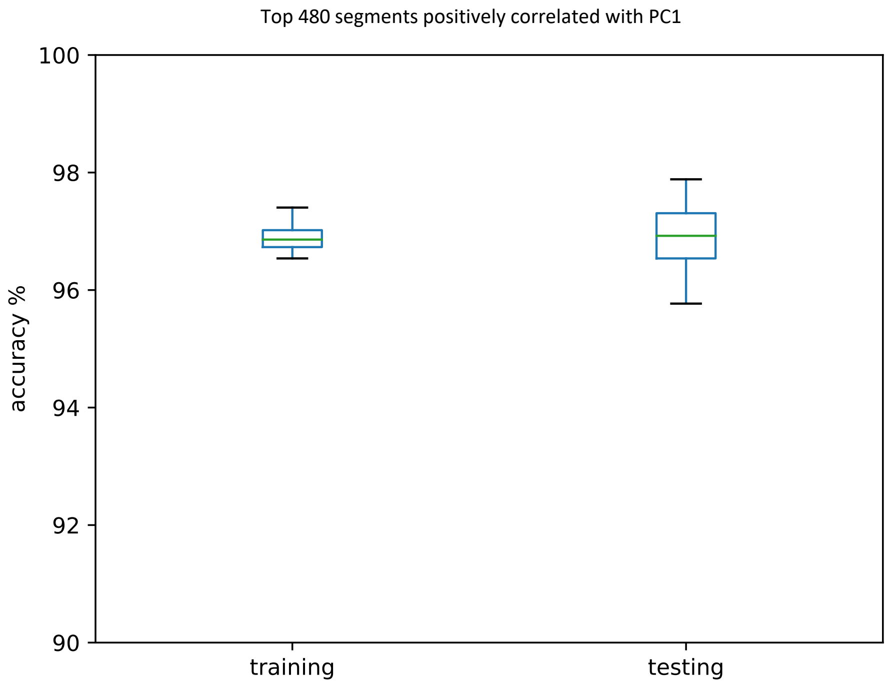
Top 480 segments positively correlated with PC1
100
98
96
94
92
90
accuracy %
training
testing

Supplementary Table S1

ID	sample_type	Age	Baseline(BL) or Progression(PD)	tumor_fraction	PSA level (ng/dL)	targeted genome NGS	high_coverage WGS	targeted methylome	LP-WGBS	LP-WGS	targeted methylome on leukocytes	abiraterone (ABI) or enzalutamide (ENZ)	Start of ADT to death (months)	AR-MethSig median methylation ratio	Sample Collection
**1**	Plasma	71	BL	0.891	403.5	Yes	No	Yes	Yes	No	Yes	ABI	14.2	low	IRST
**1**	Plasma	71	PD	0.892	685	Yes	No	Yes	Yes	No	Yes	ABI	14.2	low	IRST
**2**	Plasma	42	BL	0.8	x	No	Yes	Yes	Yes	No	Yes	ABI	59.6	-	Royal Marsden
**2**	Plasma	42	PD	0.8	12.68	No	Yes	Yes	Yes	No	No	ABI	59.6	-	Royal Marsden
**3**	Plasma	82	BL	0.74	132	Yes	No	Yes	Yes	No	No	ABI	55.4	-	IRST
**3**	Plasma	82	PD	0.73	215.6	Yes	No	Yes	Yes	No	Yes	ABI	55.4	-	IRST
**4**	Plasma	76	BL	0.65	67.79	No	Yes	Yes	Yes	Yes	Yes	ENZ	95.7	-	PREMIERE trial
**5**	Plasma	90	BL	0.643	675.6	Yes	No	Yes	Yes	No	No	ABI	23.8	low	IRST
**5**	Plasma	90	PD	0.477	611.4	Yes	No	Yes	Yes	No	No	ABI	23.8	low	IRST
**6**	Plasma	80	BL	0.61	335	Yes	No	Yes	Yes	No	No	ABI	77.2	-	Royal Marsden
**7**	Plasma	80	PD	0.45	20	No	Yes	Yes	Yes	Yes	Yes	ENZ	40.1	-	PREMIERE trial
**7**	Plasma	80	BL	0.55	197.9	No	Yes	Yes	Yes	Yes	No	ENZ	40.1	-	PREMIERE trial
**8**	Plasma	76	BL	0.5	65.12	Yes	No	Yes	Yes	No	Yes	ABI	25.4	low	IRST
**8**	Plasma	76	PD	0.54	196.5	Yes	No	Yes	Yes	No	No	ABI	25.4	low	IRST
**9**	Plasma	82	PD	0.25	5.73	No	Yes	Yes	Yes	Yes	Yes	ENZ	47.1	-	PREMIERE trial
**9**	Plasma	82	BL	0.45	24.87	No	Yes	Yes	Yes	Yes	No	ENZ	47.1	-	PREMIERE trial
**10**	Plasma	78	BL	0.418	221.4	Yes	No	Yes	Yes	No	No	ABI	39	-	IRST
**11**	Plasma	66	BL	0.353	6.8	Yes	No	No	Yes	No	No	ABI	35.6	-	IRST
**11**	Plasma	66	PD	0.64	6.9	Yes	No	No	Yes	No	No	ABI	35.6	-	IRST
**12**	Plasma	63	PD	0.18	28.49	No	Yes	Yes	Yes	Yes	Yes	ENZ	20	-	PREMIERE trial
**12**	Plasma	63	BL	0.325	33.02	No	Yes	Yes	Yes	Yes	No	ENZ	20	-	PREMIERE trial
**13**	Plasma	90	BL	0.31	141.2	Yes	No	Yes	Yes	No	Yes	ABI	17.2	-	IRST
**13**	Plasma	90	PD	0.18	96.1	Yes	No	Yes	Yes	No	No	ABI	17.2	-	IRST
**14**	Plasma	83	BL	0.301	71.86	Yes	No	No	Yes	No	No	ABI	50.1	-	IRST
**14**	Plasma	83	PD	0.227	98.43	Yes	No	No	Yes	No	No	ABI	50.1	-	IRST
**15**	Plasma	70	BL	0.227	58.53	Yes	No	No	Yes	No	No	ABI	20.7	-	IRST
**15**	Plasma	70	PD	0.013	6.89	Yes	No	No	Yes	No	No	ABI	20.7	-	IRST
**16**	Plasma	69	BL	0.21	92	Yes	No	Yes	Yes	No	No	ABI	58.4	-	Royal Marsden
**17**	Plasma	70	BL	0.18	3150	Yes	No	Yes	Yes	No	No	ABI	96.3	-	Royal Marsden
**18**	Plasma	75	BL	0.134	3.5	Yes	No	Yes	Yes	No	No	ABI	80.9	-	IRST
**18**	Plasma	75	PD	0.57	5.89	Yes	No	Yes	Yes	No	No	ABI	80.9	-	IRST
**19**	Plasma	78	BL	0.128	126.1	Yes	No	Yes	Yes	No	No	ABI	29	low	IRST
**19**	Plasma	78	PD	0.315	488.7	Yes	No	Yes	Yes	No	No	ABI	29	low	IRST
**20**	Plasma	62	BL	0.12	54.26	Yes	No	Yes	Yes	No	No	ABI	60.1	-	IRST
**20**	Plasma	62	PD	0.051	83.47	Yes	No	Yes	Yes	No	No	ABI	60.1	-	IRST
**21**	Plasma	82	BL	0.096	69.89	Yes	No	No	Yes	No	No	ABI	35.5	-	IRST
**21**	Plasma	82	PD	0.386	7.52	Yes	No	No	Yes	No	No	ABI	35.5	-	IRST
**22**	Plasma	70	BL	0.094	53.83	Yes	No	No	Yes	No	No	ABI	49.1	-	IRST
**22**	Plasma	70	PD	0.218	551.9	Yes	No	No	Yes	No	No	ABI	49.1	-	IRST
**23**	Plasma	65	BL	0.046	25.98	Yes	No	No	Yes	No	No	ABI	104.5	-	IRST
**24**	Plasma	76	BL	0.04	277	Yes	No	Yes	No	No	Yes	ABI	74	-	IRST
**24**	Plasma	76	PD	0.09	50.07	Yes	No	Yes	No	No	x	ABI	74	-	IRST
**24**	Plasma	76	PD_2	0.354	684	Yes	No	Yes	Yes	No	No	ENZ	74	-	IRST
**24**	Plasma	76	PD_3	0.774	825	Yes	No	Yes	Yes	No	No	ENZ	74	-	IRST
**25**	Plasma	67	BL	0.05	4.15	Yes	No	Yes	Yes	No	No	ABI	17.5	-	IRST
**25**	Plasma	67	PD	0.086	2.84	Yes	No	Yes	Yes	No	No	ABI	17.5	-	IRST
**HV1**	Plasma	30	HV1_R1	0	x	No	No	Yes	Yes	No	Yes	x	x	-	x
**HV1**	Plasma	30	HV1_R2	0	x	No	No	Yes	Yes	No	Yes	x	x	-	x
**HV2**	Plasma	60	HV2_R1	0	x	No	No	Yes	No	No	Yes	x	x	-	x
**HV2**	Plasma	60	HV2_R2	0	x	No	No	Yes	No	No	Yes	x	x	-	x

Supplementary Table S2

ID	Baseline(BL) or Progression(PD)	sample_type	**targeted methylome**	Total Sequences	Mapped Reads	% Mapped Reads	% bisulfite conversion
1	BL	Plasma	Yes	186555220	167111560	89.58%	96.6%
1	PD	Plasma	Yes	200776658	179727102	89.52%	96.5%
2	BL	Plasma	Yes	65664842	58274518	88.75%	97.0%
2	PD	Plasma	Yes	69840940	62048086	88.84%	97.0%
3	BL	Plasma	Yes	152787676	137065257	89.71%	96.7%
3	PD	Plasma	Yes	165073482	147544256	89.38%	96.5%
4	BL	Plasma	Yes	294076770	248481849	84.50%	95.4%
5	BL	Plasma	Yes	58272348	51930705	89.12%	96.7%
5	PD	Plasma	Yes	46653168	41603630	89.18%	96.6%
6	BL	Plasma	Yes	155781620	135133214	86.75%	97.1%
7	PD	Plasma	Yes	78308978	68536718	87.52%	96.5%
7	BL	Plasma	Yes	47126584	39744701	84.34%	96.2%
8	BL	Plasma	Yes	168923880	150406167	89.04%	96.6%
8	PD	Plasma	Yes	200709408	178679515	89.02%	96.5%
9	PD	Plasma	Yes	290996960	249810225	85.85%	96.5%
9	BL	Plasma	Yes	368847482	318176786	86.26%	96.6%
10	BL	Plasma	Yes	48419738	40566381	83.78%	97.1%
11	BL	Plasma	No	x	x	x	x
11	PD	Plasma	No	x	x	x	x
12	PD	Plasma	Yes	329218080	279039378	84.76%	96.4%
12	BL	Plasma	Yes	92879856	76751623	82.64%	96.2%
13	BL	Plasma	Yes	183498796	164867778	89.85%	96.5%
13	PD	Plasma	Yes	201791470	179503148	88.95%	96.6%
14	BL	Plasma	No	x	x	x	x
14	PD	Plasma	No	x	x	x	x
15	BL	Plasma	No	x	x	x	x
15	PD	Plasma	No	x	x	x	x
16	BL	Plasma	Yes	148832250	128633440	86.43%	97.0%
17	BL	Plasma	Yes	136306032	116853097	85.73%	96.9%
18	BL	Plasma	Yes	62626728	55853347	89.18%	96.7%
18	PD	Plasma	Yes	51544194	45752062	88.76%	96.8%
19	BL	Plasma	Yes	26710136	23904934	89.50%	97.2%
19	PD	Plasma	Yes	32932662	29646068	90.02%	96.9%
20	BL	Plasma	Yes	106508740	95230613	89.41%	96.8%
20	PD	Plasma	Yes	120300158	107326545	89.22%	96.7%
21	BL	Plasma	No	x	x	x	x
21	PD	Plasma	No	x	x	x	x
22	BL	Plasma	No	x	x	x	x
22	PD	Plasma	No	x	x	x	x
23	BL	Plasma	No	x	x	x	x
24	BL	Plasma	Yes	136490762	121086940	88.71%	96.6%
24	PD	Plasma	Yes	150877238	135508492	89.81%	96.6%
24	PD_2	Plasma	Yes	44467698	39895075	89.72%	97.1%
24	PD_3	Plasma	Yes	50086012	44301319	88.45%	96.1%
25	BL	Plasma	Yes	57279412	51098610	89.21%	96.5%
25	PD	Plasma	Yes	58802244	52411556	89.13%	96.4%
HV1	HV1_R1	Plasma	Yes	56437928	51092703	90.53%	96.9%
HV1	HV1_R2	Plasma	Yes	93236514	82308864	88.28%	96.6%
HV2	HV2_R1	Plasma	Yes	29924470	27088855	90.52%	96.7%
HV2	HV2_R2	Plasma	Yes	139484410	123219652	88.34%	96.9%

Supplementary Table S3

ID	Baseline(BL) or Progression(PD)	sample_type	**LP-WGBS**	Total Sequences	Mapped Reads	% Mapped Reads	% bisulfite conversion
1	BL	Plasma	Yes	61555740	54210537	88.1%	96.6%
1	PD	Plasma	Yes	63848530	55948225	87.6%	96.5%
2	BL	Plasma	Yes	42761332	36870167	86.2%	96.0%
2	PD	Plasma	Yes	42617996	36617189	85.9%	96.4%
3	BL	Plasma	Yes	59129422	52246290	88.4%	96.7%
3	PD	Plasma	Yes	56151334	49450718	88.1%	96.5%
4	BL	Plasma	Yes	66690658	55297446	82.9%	95.4%
5	BL	Plasma	Yes	41154970	35842986	87.1%	96.7%
5	PD	Plasma	Yes	42454336	36955379	87.0%	96.6%
6	BL	Plasma	Yes	63468228	54152133	85.3%	97.1%
7	PD	Plasma	Yes	58724038	50405549	85.8%	96.5%
7	BL	Plasma	Yes	52757540	44105950	83.6%	96.4%
8	BL	Plasma	Yes	48997884	42906582	87.6%	96.6%
8	PD	Plasma	Yes	57210482	50038902	87.5%	96.5%
9	PD	Plasma	Yes	62950726	52762980	83.8%	96.5%
9	BL	Plasma	Yes	61130412	51111528	83.6%	96.6%
10	BL	Plasma	Yes	63448740	52815910	83.2%	97.1%
11	BL	Plasma	Yes	45050378	37661639	83.6%	95.8%
11	PD	Plasma	Yes	50541554	42458690	84.0%	96.0%
12	PD	Plasma	Yes	67810208	55181787	81.4%	96.5%
12	BL	Plasma	Yes	52569972	43274790	82.3%	96.4%
13	BL	Plasma	Yes	63728198	56487135	88.6%	96.5%
13	PD	Plasma	Yes	58990260	51675897	87.6%	96.4%
14	BL	Plasma	Yes	59532904	49524710	83.2%	96.5%
14	PD	Plasma	Yes	54159938	44737438	82.6%	96.8%
15	BL	Plasma	Yes	50568866	42863081	84.8%	96.0%
15	PD	Plasma	Yes	53716688	44653610	83.1%	95.9%
16	BL	Plasma	Yes	67981380	58207701	85.6%	97.0%
17	BL	Plasma	Yes	77716154	63939783	82.3%	96.9%
18	BL	Plasma	Yes	46956924	40616653	86.5%	96.7%
18	PD	Plasma	Yes	40703206	35013233	86.0%	96.7%
19	BL	Plasma	Yes	61515246	52087141	84.7%	97.3%
19	PD	Plasma	Yes	67569626	57153038	84.6%	96.9%
20	BL	Plasma	Yes	55356132	48049486	86.8%	96.8%
20	PD	Plasma	Yes	50586228	43874943	86.7%	96.7%
21	BL	Plasma	Yes	54428970	44556958	81.9%	96.0%
21	PD	Plasma	Yes	49474000	41833481	84.6%	96.0%
22	BL	Plasma	Yes	50742732	40663027	80.1%	96.5%
22	PD	Plasma	Yes	59781268	48210928	80.6%	96.5%
23	BL	Plasma	Yes	54949210	45533981	82.9%	96.5%
24	BL	Plasma	No	x	x	x	x
24	PD	Plasma	No	x	x	x	x
24	PD_2	Plasma	Yes	80786148	67488386	83.5%	97.1%
24	PD_3	Plasma	Yes	36084166	29019487	80.4%	96.1%
25	BL	Plasma	Yes	44581236	38693632	86.8%	96.5%
25	PD	Plasma	Yes	45563992	39312802	86.3%	96.4%
HV1	HV1_R1	Plasma	Yes	61954404	52870463	85.3%	96.9%
HV1	HV1_R2	Plasma	Yes	80314646	66651587	83.0%	96.6%
HV2	HV2_R1	Plasma	No	x	x	x	x
HV2	HV2_R2	Plasma	No	x	x	x	x

Supplementary Table S4

patient	sample_id	metastatic sites	time on ADT (month)	time from ADT to death (month)	AR-MethSig median methylation ratio
CA27	M225	base of bladder	51.0	84.0	-
CA27	M231	spine lumbar	51.0	84.0	-
CA27	M226	dural base skull	51.0	84.0	-
CA27	M219	right adrenal gland	51.0	84.0	-
CA34	M334	Liver: right lobe A	26.5	58.5	low
CA34	M336	Liver: right lobe C	26.5	58.5	low
CA34	M337	Liver: left lobe A	26.5	58.5	low
CA35	M442	Left para-aortic lymph node track	60.0	168.0	-
CA35	M442B	Left para-aortic lymph node track B	60.0	168.0	-
CA36	M294	Roof left orbit - brain	27.0	66.0	-
CA36	M296	Dura - left orbit	27.0	66.0	-
CA36	M307	Liver: right lobe deposit C	27.0	66.0	-
CA43	M437	Liver: left lobe nodule 1	3.0	34.0	low

Supplementary Table S5

Genes overlapping with AR-MethSig

WNT16	NPY	CBS	TUSC5
MEOX1	CNTFR	SPTBN4	SORCS2
ASB4	SARDH	PGLYRP2	ADARB2
FYN	PREX1	DMKN	ADAMTSL5
ZBTB32	PTGIS	JAK1	KLK12
MAN2B2	KCNK15	MEGF6	MPPED1
INSRR	RUNX2	DISC1	TMPRSS6
ELN	HS3ST3B1	PQLC3	SAMD11
HEXB	TMEM74B	PF4	ERC2
FSTL4	RFPL3	ABLIM2	GABRD
FOXN3	CBFA2T3	ANKRD33B	DNAH17
HHAT	ZSCAN10	HIST1H2AA	LRRIQ4
CAMK2B	EXOC3L2	AQP3	PDCD1
DGKG	COL5A1	ELFN2	KRTDAP
TLE2	KHDRBS3	MS4A8	LINC00523
SLC9A3	NREP	PRRX2	AJAP1
KIF26A	SRPK2	C16orf92	ANXA6
RORA	SCRN1	ZNF180	LINC00336
SPTB	IGF2BP3	LY6D	DNM3
CNGB1	LMX1B	C11orf85	DLGAP2
ST6GALNAC2	MGARP	DEGS2	CARD11
EPHA8	MAP2K5	KLHL30	NTRK1
IGF2BP2	ITGA11	SFTPC	ZNF583
PAG1	BCAR3	KCTD19	C2CD4A
PKD2L2	CDK15	PCSK9	SLC34A3
CRYBG3	SLC38A4	WNT10B	SMOC1
OPRK1	GALNS	LRRN2	RASSF9
PILRA	KSR1	MGMT	MUC2
TGFB2	ARSG	KSR2	C1orf95
BAMBI	ASGR1	DSCAM	IGFL2
HPS4	MMEL1	P2RY6	PCDHGA1
CACNA1I	PRDM16	PLEKHG5	EXOC3L4
PVALB	FHAD1	CAMTA1	MIR548D2
RIN3	SUSD4	NXNL1	MIR133A2
ASB2	KCNN3	FBXL14	ARL2
NTSR1	MEIS1	PRND	MIR1268A
CHRNA4	GULP1	LRRC15	EBF2
CCM2L	PTH2R	RCAN2	PLXNA4
MGRN1	IQSEC1	DAB1	URAHP
PLLP	SLC25A26	C2orf70	LINC00703
ZNF423	NKD2	SLC6A19	LINC00162
WFDC1	ANKRD31	LEP	LINC00705
FAM189A1	SLC17A4	AMZ1	ELFN1
CGB	HIST1H2BA	GPR152	MROH5
ZFR2	ANO7	CABP4	STEAP2-AS1
BBC3	SLC2A12	LINC00521	LINC00704
ZNRF4	C7orf50	DLEU1	LINC00689
COMP	SDK1	KBTBD11	EMBP1
VIPR2	NTMT1	OR51F2	ADAM6
CPVL	PARD3	UMODL1	DPY19L2P4
CHN2	SERPING1	C2orf73	ERICH1-AS1
CRHR2	GRIK4	UCN3	TDGF1
CLIP2	GGTLC1	C9orf50	MICAL3
CDH23	PLCH2	HTR1D	ETV5
EBF3	SCN2B	TH	LINC00535
RGS9	CDH22	PAK2	FMN1
SOD3	NUDT22	C9orf139	GPR162
FAM149A	CCDC3	FUT7	KBTBD11-OT1
DNAJC4	VENTX	AATK	CCDC177
BIRC2	TRIM36	CCDC172	MIR548W
CALCA	PITPNC1	CAMK1D	ESPNP
SLC6A12	FGD5	URAD	TRABD2B
FGF1	ODF1	ASCL2	TSNAX-DISC1
RBP1	KCNMA1	B3GALT5	
EFCC1	CACNA2D3	SLC35F3	
PLCD4	MEGF11	ARSI	
NR5A2	RADIL	TBX1	
KIF17	GDPD5	KCNJ12	
ESRRB	SCUBE1	PIWIL3	
NPY	SPON2	C14orf180	

Supplementary Table S6

Term	Type	ID	Input.numbe	Background.r	P.value	adjusted.P.value
MIKKELSEN_MCV6_HCP_WITH_H3K27ME3	MSigDB	M1954	28	431	3.58E-11	1.19E-07
MARTENS_TRETINOIN_RESPONSE_UP	MSigDB	M2098	35	706	1.64E-10	2.71E-07
BENPORATH_ES_WITH_H3K27ME3	MSigDB	M10371	37	995	1.08E-07	0.000119034
BENPORATH_EED_TARGETS	MSigDB	M7617	34	921	5.45E-07	0.000451695
BENPORATH_SUZ12_TARGETS	MSigDB	M9898	32	924	5.37E-06	0.003561478
MEISSNER_NPC_HCP_WITH_H3K4ME3_AND_H3K27ME3	MSigDB	M1935	10	144	2.96E-05	0.016353513
NAKAMURA_METASTASIS_MODEL_DN	MSigDB	M15940	5	39	4.58E-05	0.01899284
MEISSNER_NPC_HCP_WITH_H3_UNMETHYLATED	MSigDB	M1936	20	487	4.16E-05	0.01899284
MEISSNER_BRAIN_HCP_WITH_H3K4ME3_AND_H3K27ME3	MSigDB	M1941	32	1035	5.34E-05	0.019656077
BENPORATH_PRC2_TARGETS	MSigDB	M8448	22	594	8.33E-05	0.027602582

Supplementary Table S7

CHR	START of ar-MethSig	END of ar-MethSig	REG_STRAN	ABS_SITE_START	ABS_SITE_END	REL_SITE_ST	REL_SITE_EN	SITE_STRAN	SCORE	SITE
chr14	105052526	105052675	+	105052565	105052581	-36	-21	+	0.876674	GGGAACAGGATTTACCT
chr14	105052476	105052625	+	105052565	105052581	14	29	+	0.876674	GGGAACAGGATTTACCT
chr14	105052426	105052575	+	105052565	105052581	64	79	+	0.876674	GGGAACAGGATTTACCT
chr1	861676	861825	+	861753	861769	2	17	-	0.872295	GGGGTCACCGTGTTCTC
chr4	7252376	7252525	+	7252514	7252530	63	78	-	0.871746	AAGAACAGTGGGTTCCC
chr15	41219376	41219525	+	41219494	41219510	43	58	+	0.866725	GGGTAGAGAGTGTTCCA
chr2	113379876	113380025	+	113379977	113379993	26	41	+	0.854291	AGCCACACGGTGTGCCT
chr1	48360376	48360525	+	48360443	48360459	-8	7	+	0.852908	GTGAACATGACGTACCA
chr1	48360326	48360475	+	48360443	48360459	42	57	+	0.852908	GTGAACATGACGTACCA
chr1	200707026	200707175	+	200707112	200707128	11	26	+	0.852876	AAGAACAGGGTGCTCCG
chr1	200706976	200707125	+	200707112	200707128	61	76	+	0.852876	AAGAACAGGGTGCTCCG
chr14	106320426	106320575	+	106320481	106320497	-20	-5	+	0.845683	AGGGACACGTTGTACAG
chr2	121279826	121279975	+	121279826	121279842	-75	-60	+	0.840785	GGGAATGTGCTGTACCC
chr2	121279776	121279925	+	121279826	121279842	-25	-10	+	0.840785	GGGAATGTGCTGTACCC
chr2	121279726	121279875	+	121279826	121279842	25	40	+	0.840785	GGGAATGTGCTGTACCC
chr15	60919376	60919525	+	60919386	60919402	-65	-50	+	0.832409	GGGTACACAGTGATCAG
chr15	60919326	60919475	+	60919386	60919402	-15	0	+	0.832409	GGGTACACAGTGATCAG
chr1	15655876	15656025	+	15655958	15655974	7	22	+	0.831211	AGGCACGCGCTGTCCTG
chr1	15655826	15655975	+	15655958	15655974	57	72	+	0.831211	AGGCACGCGCTGTCCTG
chr5	142005126	142005275	+	142005141	142005157	-60	-45	-	0.830344	TGGTACACCACGGTCCA
chr14	105044876	105045025	+	105044899	105044915	-52	-37	+	0.822933	GAGCGCACTGTGTGCCA
chr14	105044826	105044975	+	105044899	105044915	-2	13	+	0.822933	GAGCGCACTGTGTGCCA
chr3	169540226	169540375	+	169540253	169540269	-48	-33	-	0.821421	AGGAGCTCTGTGTTCTC
chr3	169540176	169540325	+	169540253	169540269	2	17	-	0.821421	AGGAGCTCTGTGTTCTC
chr7	2959076	2959225	+	2959089	2959105	-62	-47	+	0.820473	TGGTACATGGTGTCACG
chr7	2959026	2959175	+	2959089	2959105	-12	3	+	0.820473	TGGTACATGGTGTCACG
chr5	73969126	73969275	+	73969228	73969244	27	42	-	0.817906	TGCCACAGAATGTTCCC
chr14	103691276	103691425	+	103691303	103691319	-48	-33	-	0.817436	TGGTCCACTGCGTCCCT
chr14	103691226	103691375	+	103691303	103691319	2	17	-	0.817436	TGGTCCACTGCGTCCCT
chr16	4673826	4673975	+	4673954	4673970	53	68	-	0.814584	GGGAACACCTTGCACAC
chr9	136566976	136567125	+	136567043	136567059	-8	7	-	0.814301	CAGCACAGCATGAACCC
chr9	136566926	136567075	+	136567043	136567059	42	57	-	0.814301	CAGCACAGCATGAACCC
chr3	64338476	64338625	+	64338481	64338497	-70	-55	-	0.813636	AAGAACAGTATGTCTCT
chr3	64338426	64338575	+	64338481	64338497	-20	-5	-	0.813636	AAGAACAGTATGTCTCT
chr17	60828076	60828225	+	60828091	60828107	-60	-45	-	0.810002	CATCACACCCTGTTCCA
chr7	44279576	44279725	+	44279698	44279714	47	62	+	0.803908	AGGTACAGCTTGTCCAG
chr4	140201576	140201725	+	140201587	140201603	-64	-49	-	0.803644	TGGCTCATTCTGTACCA
chr4	140201526	140201675	+	140201587	140201603	-14	1	-	0.803644	TGGCTCATTCTGTACCA
chr10	3797376	3797525	+	3797383	3797399	-68	-53	+	0.802711	GGGAACACGTCATGCTC
chr10	3797326	3797475	+	3797383	3797399	-18	-3	+	0.802711	GGGAACACGTCATGCTC
chr4	26493326	26493475	+	26493377	26493393	-24	-9	+	0.802679	GGGTCCTGGGTGTTCCA
chr5	170743776	170743925	+	170743888	170743904	37	52	+	0.79747	GGGAAGGCGGTTTTCCT
chr7	65970026	65970175	+	65970143	65970159	42	57	+	0.797394	CAGACCAGGTTGTACTC
chr8	125249776	125249925	+	125249789	125249805	-62	-47	+	0.796722	GAGGACAGAGCTTTCCC
chr14	104623526	104623675	+	104623620	104623636	19	34	+	0.796695	GGGCAATTAGTGTTCCT
chr16	2863726	2863875	+	2863767	2863783	-34	-19	+	0.794934	ACGAACACCGTGTGATT
chr5	180597576	180597725	+	180597592	180597608	-59	-44	-	0.794353	CAGCACACACTGGCCCC
chr5	180597526	180597675	+	180597592	180597608	-9	6	-	0.794353	CAGCACACACTGGCCCC
chr5	180597476	180597625	+	180597592	180597608	41	56	-	0.794353	CAGCACACACTGGCCCC
chr19	5455226	5455375	+	5455365	5455381	64	79	+	0.792216	TGGGACAGAATGTAGCA
chr12	322876	323025	+	322897	322913	-54	-39	+	0.790961	GGGCACCCCTTGTCCTG
chr12	322826	322975	+	322897	322913	-4	11	+	0.790961	GGGCACCCCTTGTCCTG
chr6	168629976	168630125	+	168629993	168630009	-58	-43	+	0.790707	TGGAACCAAGAGTTCCC
chr6	168629926	168630075	+	168629993	168630009	-8	7	+	0.790707	TGGAACCAAGAGTTCCC
chr6	168629876	168630025	+	168629993	168630009	42	57	+	0.790707	TGGAACCAAGAGTTCCC
chr2	26788626	26788775	+	26788750	26788766	49	64	+	0.789367	GGTTCCATGCTGTTCCT
chr17	26795176	26795325	+	26795229	26795245	-22	-7	+	0.785916	GAGGACTTGGTCTTCCC
chr7	89747876	89748025	+	89747885	89747901	-66	-51	-	0.78478	CAGTAAACGCTGTCCCG
chr7	89747826	89747975	+	89747885	89747901	-16	-1	-	0.78478	CAGTAAACGCTGTCCCG
chr22	43827726	43827875	+	43827739	43827755	-62	-47	-	0.784424	CAGCACATAATGAGCTC
chr3	194097026	194097175	+	194097117	194097133	16	31	-	0.783168	TGGAACACTGGGACCCT
chr3	194096976	194097125	+	194097117	194097133	66	81	-	0.783168	TGGAACACTGGGACCCT
chr19	35981426	35981575	+	35981490	35981506	-11	4	+	0.7821	AGGCATGGACTGTTCCT
chr19	35981376	35981525	+	35981490	35981506	39	54	+	0.7821	AGGCATGGACTGTTCCT
chr10	3568726	3568875	+	3568746	3568762	-55	-40	-	0.782035	AAGAACTAAGTGTTACT
chr19	36195326	36195475	+	36195411	36195427	10	25	-	0.78194	GGGAGCGTTGCGTCCCT
chr19	36195276	36195425	+	36195411	36195427	60	75	-	0.78194	GGGAGCGTTGCGTCCCT
chr10	131357076	131357225	+	131357194	131357210	43	58	+	0.781329	GGGGGCGGCGTGTGCTC
chr9	33448176	33448325	+	33448220	33448236	-31	-16	+	0.781194	GAGCACGTCGTGTGTCG
chr7	1251226	1251375	+	1251295	1251311	-6	9	-	0.780791	TGGTCCACAGGGTCCCT
chr7	1251176	1251325	+	1251295	1251311	44	59	-	0.780791	TGGTCCACAGGGTCCCT
chr4	1564076	1564225	+	1564145	1564161	-6	9	+	0.780756	GAGGACAGAGTGGCCCA
chr4	1564026	1564175	+	1564145	1564161	44	59	+	0.780756	GAGGACAGAGTGGCCCA
chr22	37499726	37499875	+	37499799	37499815	-2	13	+	0.780437	AAGTACATGGGGTGCAG
chr10	131744276	131744425	+	131744286	131744302	-65	-50	-	0.780002	AAAAAAATCCTGTTCCT
chr19	17138776	17138925	+	17138916	17138932	65	80	-	0.779974	TCCAACACCCTGTTCTT
chr4	3752176	3752325	+	3752188	3752204	-63	-48	-	0.778441	GGGCTCTCCTTGTTCTT
chr22	43621726	43621875	+	43621831	43621847	30	45	-	0.777512	ATGAACAACGCTTTCCT
chr17	21278776	21278925	+	21278779	21278795	-72	-57	-	0.777355	GGGCACAGACTGGCCTC
chr10	94448376	94448525	+	94448391	94448407	-60	-45	+	0.776906	TAGACCATTATGTACAT
chr16	90114976	90115125	+	90114994	90115010	-57	-42	-	0.776834	AATAACACATTCTTCCT
chr2	54560476	54560625	+	54560535	54560551	-16	-1	+	0.776389	GCGAACAGCATGTGCAG
chr4	74847726	74847875	+	74847760	74847776	-41	-26	-	0.775917	CGGCACGTTTTATTCCC
chr17	60214476	60214625	+	60214519	60214535	-32	-17	-	0.775747	CGGCACACCTGGTTCAT
chr21	47399476	47399625	+	47399559	47399575	8	23	-	0.775201	TTACACAACGTGTTCCT
chr1	2424626	2424775	+	2424666	2424682	-35	-20	-	0.773081	TGGGTCAGCGCGTGCCT
chr1	2424576	2424725	+	2424666	2424682	15	30	-	0.773081	TGGGTCAGCGCGTGCCT
chr1	2424526	2424675	+	2424666	2424682	65	80	-	0.773081	TGGGTCAGCGCGTGCCT
chr5	137225226	137225375	+	137225274	137225290	-27	-12	-	0.771242	AGGAACTCTGCGGCCCC
chr5	137225176	137225325	+	137225274	137225290	23	38	-	0.771242	AGGAACTCTGCGGCCCC
chr17	79109726	79109875	+	79109790	79109806	-11	4	-	0.77075	AAGGACACCCTGCACAC
chr17	79109676	79109825	+	79109790	79109806	39	54	-	0.77075	AAGGACACCCTGCACAC
chr13	31620276	31620425	+	31620408	31620424	57	72	-	0.770562	AGGCCCATCGGGTGCCT
chr14	106208326	106208475	+	106208348	106208364	-53	-38	+	0.770025	CGGTACGTGCTGTTGTA
chr14	106208276	106208425	+	106208348	106208364	-3	12	+	0.770025	CGGTACGTGCTGTTGTA
chr11	72980776	72980925	+	72980865	72980881	14	29	-	0.769534	CGGAACAAGCTTTCCCA
chr11	72980726	72980875	+	72980865	72980881	64	79	-	0.769534	CGGAACAAGCTTTCCCA
chr7	564176	564325	+	564182	564198	-69	-54	+	0.76916	GGGGACACGGCGTTAAG
chr16	1159776	1159925	+	1159853	1159869	2	17	+	0.768869	GATCACACGCTGATCCT
chr16	1159726	1159875	+	1159853	1159869	52	67	+	0.768869	GATCACACGCTGATCCT

Supplementary Table S7 (con’d)

chr1	223435676	223435825	+	223435710	223435726	-41	-26	-	0.768226	GTGCAAATCGTGTGCTT
chr1	223435626	223435775	+	223435710	223435726	9	24	-	0.768226	GTGCAAATCGTGTGCTT
chr8	54164576	54164725	+	54164615	54164631	-36	-21	+	0.766828	AGTAACAAAATGATCCA
chr8	54164526	54164675	+	54164615	54164631	14	29	+	0.766828	AGTAACAAAATGATCCA
chr16	57916776	57916925	+	57916899	57916915	48	63	+	0.76667	AGGCAGAGGATGCTCCT
chr19	56914726	56914875	+	56914807	56914823	6	21	-	0.766002	CAGAACTCACTGAACTC
chr19	56914676	56914825	+	56914807	56914823	56	71	-	0.766002	CAGAACTCACTGAACTC
chr22	25160776	25160925	+	25160855	25160871	4	19	+	0.765194	AGGAACAGTGTGGTTTG
chr14	89881626	89881775	+	89881628	89881644	-73	-58	+	0.765096	GACAACATGCTGCTCTC
chr7	158800876	158801025	+	158800989	158801005	38	53	-	0.764857	TGGTACTTTCTGTATTT
chr21	44494676	44494825	+	44494747	44494763	-4	11	-	0.763198	CAGAAAGCCGTGGACCC
chr21	44494626	44494775	+	44494747	44494763	46	61	-	0.763198	CAGAAAGCCGTGGACCC
chr8	142452376	142452525	+	142452500	142452516	49	64	-	0.763149	GGTGTCACCGTGTGCTT
chr12	111136976	111137125	+	111137035	111137051	-16	-1	+	0.762786	GGGCACATTTCGTGTTC
chr3	64225026	64225175	+	64225123	64225139	22	37	+	0.761576	GGGAACATGGAGATGCT
chr3	196515476	196515625	+	196515525	196515541	-26	-11	-	0.761283	GTGCACAACATGTGTCC
chr3	128724926	128725075	+	128724958	128724974	-43	-28	+	0.761114	AGTTAAGTAATGTACCC
chr3	128724876	128725025	+	128724958	128724974	7	22	+	0.761114	AGTTAAGTAATGTACCC
chr9	122734526	122734675	+	122734566	122734582	-35	-20	-	0.761051	CAGAAGAAATTGTGCTT
chr9	122734476	122734625	+	122734566	122734582	15	30	-	0.761051	CAGAAGAAATTGTGCTT
chr2	242797776	242797925	+	242797796	242797812	-55	-40	-	0.760926	CAGGACATTGCTTTCCA
chr2	242797726	242797875	+	242797796	242797812	-5	10	-	0.760926	CAGGACATTGCTTTCCA
chr2	242797676	242797825	+	242797796	242797812	45	60	-	0.760926	CAGGACATTGCTTTCCA
chr2	27938076	27938225	+	27938200	27938216	49	64	+	0.760802	GGGGACACCATGGTGCA
chr5	137225326	137225475	+	137225357	137225373	-44	-29	+	0.76041	AGGGACAGATGGTCCCA
chr5	137225276	137225425	+	137225357	137225373	6	21	+	0.76041	AGGGACAGATGGTCCCA
chr7	1329376	1329525	+	1329404	1329420	-47	-32	+	0.760336	GGGGACGGGGGGAACCC
chr7	1329326	1329475	+	1329404	1329420	3	18	+	0.760336	GGGGACGGGGGGAACCC
chr7	1329276	1329425	+	1329404	1329420	53	68	+	0.760336	GGGGACGGGGGGAACCC
chr1	3347876	3348025	+	3347983	3347999	32	47	+	0.760279	TGGGACAGCGCGTGCAG
chr3	196515526	196515675	+	196515643	196515659	42	57	+	0.760258	GGGAAGAGCCTGTTTCA
chr14	93154676	93154825	+	93154804	93154820	53	68	-	0.760198	CGAGACGGCATGTTCTT
chr3	194090526	194090675	+	194090660	194090676	59	74	-	0.75894	CAGGACACATTTTCCTC
chr1	204655126	204655275	+	204655190	204655206	-11	4	+	0.75865	AGTCACAGCATGCACCT
chr1	204655076	204655225	+	204655190	204655206	39	54	+	0.75865	AGTCACAGCATGCACCT
chr22	50457076	50457225	+	50457172	50457188	21	36	-	0.758632	GGCTGCAACCTGTACTC
chr22	37771226	37771375	+	37771275	37771291	-26	-11	-	0.758478	AGGAAGGCACCGTCCTC
chr22	37771176	37771325	+	37771275	37771291	24	39	-	0.758478	AGGAAGGCACCGTCCTC
chr14	105105026	105105175	+	105105144	105105160	43	58	+	0.758448	GAGAACACAGCGTAGGG
chr2	159705526	159705675	+	159705566	159705582	-35	-20	-	0.756755	AAGTAACCCCTGTGCTT
chr2	159705476	159705625	+	159705566	159705582	15	30	-	0.756755	AAGTAACCCCTGTGCTT
chr5	111090026	111090175	+	111090131	111090147	30	45	+	0.756378	AAGTAGGTAACGTTCTG
chr15	99974726	99974875	+	99974826	99974842	25	40	-	0.756	CAGAACACGGTGACGCT
chr14	94406426	94406575	+	94406556	94406572	55	70	+	0.75556	AGGGACAGCCAGATCCC
chr17	74237526	74237675	+	74237567	74237583	-34	-19	-	0.755025	TAGAACATGACTTTCAC
chr16	14380576	14380725	+	14380621	14380637	-30	-15	-	0.754879	CGCCACATCGTGCACTC
chr16	14380526	14380675	+	14380621	14380637	20	35	-	0.754879	CGCCACATCGTGCACTC
chr12	47219876	47220025	+	47219960	47219976	9	24	+	0.754653	GTGTACCCAGTGTTATT
chr10	3373226	3373375	+	3373332	3373348	31	46	+	0.754633	GAGAGAAGGCTGTTCTC
chr8	914376	914525	+	914488	914504	37	52	-	0.753319	TCTAACATGCTGTGCCT
chr4	3288776	3288925	+	3288790	3288806	-61	-46	+	0.753066	AGGGGCACCTAGTTCTT
chr4	3288726	3288875	+	3288790	3288806	-11	4	+	0.753066	AGGGGCACCTAGTTCTT
chr4	3288676	3288825	+	3288790	3288806	39	54	+	0.753066	AGGGGCACCTAGTTCTT
chr3	185788626	185788775	+	185788626	185788642	-75	-60	-	0.752785	AGGCACAGCCTGGTTCC
chr16	29242026	29242175	+	29242075	29242091	-26	-11	-	0.752636	GGCTATAGCCTGTGCCC
chr16	29241976	29242125	+	29242075	29242091	24	39	-	0.752636	GGCTATAGCCTGTGCCC
chr7	120967726	120967875	+	120967734	120967750	-67	-52	+	0.752458	GGGTACCCACTGAGCTG
chr19	41061926	41062075	+	41061926	41061942	-75	-60	-	0.751816	GGGGTCACGCTCTGCCC
chr9	137731726	137731875	+	137731836	137731852	35	50	+	0.751691	TGGAACTGTGTGTTTTG
chr1	210612226	210612375	+	210612353	210612369	52	67	-	0.75115	AGGAAGGCCATGTGGCT
chr14	105044926	105045075	+	105044929	105044945	-72	-57	+	0.75114	GAGAAAAGTTTGCTCCC
chr1	205913876	205914025	+	205913951	205913967	0	15	+	0.750646	GGGAAGGATGTGTGCAT
chr1	205913826	205913975	+	205913951	205913967	50	65	+	0.750646	GGGAAGGATGTGTGCAT
chr17	25798576	25798725	+	25798580	25798596	-71	-56	-	0.750629	TGGAACACAGTGTCAGG
chr22	37771276	37771425	+	37771296	37771312	-55	-40	-	0.749976	TAGGAGATCTCGTTCTT
chr6	169351276	169351425	+	169351296	169351312	-55	-40	-	0.749828	GAGAGCAACTTTTACCT
chr4	3776376	3776525	+	3776466	3776482	15	30	+	0.747761	GATTACACGTTGAGCCA
chr1	156828576	156828725	+	156828711	156828727	60	75	+	0.746071	AATGACACAGGGTTCTG
chr10	131691176	131691325	+	131691239	131691255	-12	3	-	0.743974	TGGCACACACGGTTCGG
chr5	172924726	172924875	+	172924729	172924745	-72	-57	-	0.74366	TAGGACAATGCCTTCCA
chr9	135033126	135033275	+	135033195	135033211	-6	9	-	0.743455	AGGCACGACTCATACCC
chr10	4697326	4697475	+	4697372	4697388	-29	-14	-	0.743364	CAGGACAGAACATTCTC
chr10	4697276	4697425	+	4697372	4697388	21	36	-	0.743364	CAGGACAGAACATTCTC
chr10	3494226	3494375	+	3494241	3494257	-60	-45	-	0.742628	CATTCCACATTGTTCTT
chr2	242797826	242797975	+	242797848	242797864	-53	-38	+	0.742396	AGGAACCTGCTGGCCCA
chr6	33561326	33561475	+	33561447	33561463	46	61	+	0.742236	GCGAACAGTCTGTGATT
chr3	97542076	97542225	+	97542087	97542103	-64	-49	-	0.742191	GCTTACACTGTCTTCTC
chr10	12543276	12543425	+	12543287	12543303	-64	-49	+	0.742054	CAGAGCACTGTGCACTG
chr14	104865726	104865875	+	104865747	104865763	-54	-39	-	0.742026	GAGTAAGACATCTTCCC
chr1	3534326	3534475	+	3534415	3534431	14	29	+	0.741944	GGGAACCGGGGGTTGCT
chr1	3534276	3534425	+	3534415	3534431	64	79	+	0.741944	GGGAACCGGGGGTTGCT
chr17	21278826	21278975	+	21278844	21278860	-57	-42	+	0.74184	GACAAAATGGAGTTCCC
chr20	30618776	30618925	+	30618826	30618842	-25	-10	-	0.741588	CAGGACATCGGCTACTT
chr7	99987426	99987575	+	99987456	99987472	-45	-30	-	0.740949	CCAGACACCCTGTGCCC
chr9	140127226	140127375	+	140127258	140127274	-43	-28	-	0.740884	AAGGACAACGTGGTGCT
chr9	140127176	140127325	+	140127258	140127274	7	22	-	0.740884	AAGGACAACGTGGTGCT
chr12	133178876	133179025	+	133178940	133178956	-11	4	-	0.740797	CCTAACACTGTGTTCAT
chr10	3448926	3449075	+	3449026	3449042	25	40	+	0.740721	AGGCACCAGCGGTTCCA
chr12	299626	299775	+	299638	299654	-63	-48	+	0.740254	AAGAACAGTGCGGAACT
chr1	23521276	23521425	+	23521301	23521317	-50	-35	-	0.740157	CAGCTCGCGGTTTTCCC
chr8	1644876	1645025	+	1644898	1644914	-53	-38	-	0.739654	AGGTATAGAATGGGCTC
chr8	1644826	1644975	+	1644898	1644914	-3	12	-	0.739654	AGGTATAGAATGGGCTC
chr7	1266026	1266175	+	1266161	1266177	60	75	-	0.738836	CTGAACAACGTGAGCCG
chr15	29825226	29825375	+	29825252	29825268	-49	-34	-	0.737961	TTGTACACAGGCTACTT
chr17	74581326	74581475	+	74581450	74581466	49	64	-	0.737698	CAATAGAAAGTGTACTT
chr2	3697376	3697525	+	3697391	3697407	-60	-45	+	0.73762	AGGAAACCAGAGTGCCC
chr16	89009426	89009575	+	89009486	89009502	-15	0	+	0.737386	AGGAAAACATTCTGCCG
chr16	89009376	89009525	+	89009486	89009502	35	50	+	0.737386	AGGAAAACATTCTGCCG
chr17	76522776	76522925	+	76522837	76522853	-14	1	+	0.737288	AAGTACAGAGAGTCACG
chr1	200143026	200143175	+	200143117	200143133	16	31	+	0.737135	TGGTAGAAGGTGTCCAG
chr7	1251126	1251275	+	1251231	1251247	30	45	-	0.737062	AAGAGAACTCCGTTCTC

Supplementary Table S7 (con’d)

chr20	43377476	43377625	+	43377484	43377500	-67	-52	-	0.736943	TAGCACACAGCCATCCC
chr20	43377426	43377575	+	43377484	43377500	-17	-2	-	0.736943	TAGCACACAGCCATCCC
chr3	14862826	14862975	+	14862861	14862877	-40	-25	-	0.736523	CGGTCCATCTCCTTCCC
chr1	94270076	94270225	+	94270197	94270213	46	61	+	0.736275	CAGAACAGGATGCAATT
chr8	143535776	143535925	+	143535781	143535797	-70	-55	+	0.735991	GAGCACACCGTGAGGTG
chr8	143535726	143535875	+	143535781	143535797	-20	-5	+	0.735991	GAGCACACCGTGAGGTG
chr8	143535676	143535825	+	143535781	143535797	30	45	+	0.735991	GAGCACACCGTGAGGTG
chr8	143535626	143535775	+	143535711	143535727	10	25	-	0.735584	CGGAACACCAGGGTGCC
chr8	143535576	143535725	+	143535711	143535727	60	75	-	0.735584	CGGAACACCAGGGTGCC
chr1	226756376	226756525	+	226756515	226756531	64	79	+	0.735218	AGGGACAGACCTTGCTC
chr11	67462776	67462925	+	67462837	67462853	-14	1	+	0.734221	GTGAACAGAGTTTATCT
chr11	67462726	67462875	+	67462837	67462853	36	51	+	0.734221	GTGAACAGAGTTTATCT
chr14	106174226	106174375	+	106174228	106174244	-73	-58	-	0.73318	CTGGACACGCTGTAGCA
chr14	106174176	106174325	+	106174228	106174244	-23	-8	-	0.73318	CTGGACACGCTGTAGCA
chr14	106174126	106174275	+	106174228	106174244	27	42	-	0.73318	CTGGACACGCTGTAGCA
chr8	144471976	144472125	+	144472092	144472108	41	56	+	0.732895	GAGGGCCTGGTGTGCTG
chr9	89410926	89411075	+	89410955	89410971	-46	-31	+	0.732773	AGGAGCGTGTTCTTCCA
chr9	89410876	89411025	+	89410955	89410971	4	19	+	0.732773	AGGAGCGTGTTCTTCCA
chr9	89410826	89410975	+	89410955	89410971	54	69	+	0.732773	AGGAGCGTGTTCTTCCA
chr13	106063076	106063225	+	106063080	106063096	-71	-56	-	0.732705	GAGTACATGCCTTTTTC
chr13	106063026	106063175	+	106063080	106063096	-21	-6	-	0.732705	GAGTACATGCCTTTTTC
chr13	106062976	106063125	+	106063080	106063096	29	44	-	0.732705	GAGTACATGCCTTTTTC
chr14	100624926	100625075	+	100624984	100625000	-17	-2	+	0.73183	GATTACAAAGTGTGGCA
chr14	100624876	100625025	+	100624984	100625000	33	48	+	0.73183	GATTACAAAGTGTGGCA
chr10	130844126	130844275	+	130844182	130844198	-19	-4	+	0.731576	GGCGACATTTAGTGCCC
chr4	3691226	3691375	+	3691265	3691281	-36	-21	-	0.7315	TGGTGCACTGCTTGCTT
chr11	2292976	2293125	+	2293042	2293058	-9	6	+	0.731325	TGGGACGTCGAGAACTT
chr19	14313576	14313725	+	14313654	14313670	3	18	-	0.731231	CAGCACAGGAGGGACCT
chr10	3343026	3343175	+	3343038	3343054	-63	-48	+	0.731039	AGAAACAGCGAGTAGCC
chr10	3342976	3343125	+	3343038	3343054	-13	2	+	0.731039	AGAAACAGCGAGTAGCC
chr10	3342926	3343075	+	3343038	3343054	37	52	+	0.731039	AGAAACAGCGAGTAGCC
chr22	25160376	25160525	+	25160443	25160459	-8	7	-	0.731033	TGGTAAGCACTGATCTC
chr11	75222326	75222475	+	75222344	75222360	-57	-42	+	0.731003	AAGGTCAGGGTTTGCCC
chr4	1535526	1535675	+	1535526	1535542	-75	-60	-	0.730619	GAGGCCACCATCTGCTT
chr1	226756326	226756475	+	226756329	226756345	-72	-57	+	0.730596	AAGAACAGCCAGGTCTG
chr11	92806326	92806475	+	92806352	92806368	-49	-34	-	0.73058	CAGCACTCCCCATACCC
chr14	94463726	94463875	+	94463846	94463862	45	60	-	0.730398	AACTAGACCATTTTCCC
chr11	45392476	45392625	+	45392606	45392622	55	70	-	0.730197	GAATACTTCCTGTCCTA
chr19	36004876	36005025	+	36004923	36004939	-28	-13	+	0.729784	GGGGACAGGACCCACCC
chr19	36004826	36004975	+	36004923	36004939	22	37	+	0.729784	GGGGACAGGACCCACCC
chr10	131706676	131706825	+	131706792	131706808	41	56	+	0.729328	GGAGACGGGATATACCT
chr10	4194376	4194525	+	4194404	4194420	-47	-32	-	0.728399	AGGAACAGCATGGCGCA
chr12	86230726	86230875	+	86230746	86230762	-55	-40	+	0.728111	AGGACCACAGGGCTCCA
chr12	86230676	86230825	+	86230746	86230762	-5	10	+	0.728111	AGGACCACAGGGCTCCA
chr14	103569326	103569475	+	103569374	103569390	-27	-12	-	0.727961	GGTTCCACCAGGTGCCT
chr14	103569276	103569425	+	103569374	103569390	23	38	-	0.727961	GGTTCCACCAGGTGCCT
chr10	3300426	3300575	+	3300505	3300521	4	19	+	0.727881	GAGGACAAGAGGCTCCG
chr10	3300376	3300525	+	3300505	3300521	54	69	+	0.727881	GAGGACAAGAGGCTCCG
chr10	4386776	4386925	+	4386889	4386905	38	53	+	0.727255	TGGAACAGGATGACTCA
chr5	150538326	150538475	+	150538362	150538378	-39	-24	-	0.726304	AATAAAACAGTATACCC
chr5	150538276	150538425	+	150538362	150538378	11	26	-	0.726304	AATAAAACAGTATACCC
chr5	150538226	150538375	+	150538362	150538378	61	76	-	0.726304	AATAAAACAGTATACCC
chr1	64197376	64197525	+	64197467	64197483	16	31	+	0.726278	AAGAAGGACGCGTGCTG
chr1	64197326	64197475	+	64197467	64197483	66	81	+	0.726278	AAGAAGGACGCGTGCTG
chr9	132482326	132482475	+	132482328	132482344	-73	-58	+	0.725764	TGGCACCAAGGGTTCTG
chr9	132482276	132482425	+	132482328	132482344	-23	-8	+	0.725764	TGGCACCAAGGGTTCTG
chr13	113807776	113807925	+	113807807	113807823	-44	-29	-	0.725739	AGGCACAGCTGGGTCCA
chr3	46622476	46622625	+	46622516	46622532	-35	-20	-	0.724816	GAGAAGAGCAGGTTCAC
chr11	120592076	120592225	+	120592146	120592162	-5	10	+	0.72459	AGAGATAGTTTGTTCCT
chr11	120592026	120592175	+	120592146	120592162	45	60	+	0.72459	AGAGATAGTTTGTTCCT
chr8	142452326	142452475	+	142452332	142452348	-69	-54	+	0.724217	GAGGACATGGAGAAACC
chr11	120561176	120561325	+	120561192	120561208	-59	-44	+	0.723138	AAGAACTGGCTGCGCCA
chr9	132383026	132383175	+	132383107	132383123	6	21	-	0.722221	AAGTACATGCCAGTCTT
chr5	149683176	149683325	+	149683206	149683222	-45	-30	+	0.722198	TGGAACCCGATGAGCAT
chr9	132383276	132383425	+	132383381	132383397	30	45	-	0.722094	GAGCACTTAGCCTGCCT
chr20	61979376	61979525	+	61979434	61979450	-17	-2	-	0.721773	CAGACCAAAGCCTTCCC
chr20	61979326	61979475	+	61979434	61979450	33	48	-	0.721773	CAGACCAAAGCCTTCCC
chr2	86037076	86037225	+	86037201	86037217	50	65	+	0.721425	GAATACATGAAGAACCA
chr7	1458976	1459125	+	1459060	1459076	9	24	+	0.720484	CCCTACGGGGTGTGCCC
chr9	138171626	138171775	+	138171767	138171783	66	81	+	0.720375	TGGGAGAATGTGTTCGG
chr3	127173576	127173725	+	127173601	127173617	-50	-35	-	0.720203	CAGGGCTTGGTGTCCCT
chr1	157140676	157140825	+	157140703	157140719	-48	-33	-	0.720167	GGGGGCGCCCTGGACTC
chr6	17988876	17989025	+	17988904	17988920	-47	-32	-	0.720053	GTGTACGGCAGGTGCTA
chr6	17988826	17988975	+	17988904	17988920	3	18	-	0.720053	GTGTACGGCAGGTGCTA
chr7	2728676	2728825	+	2728685	2728701	-66	-51	+	0.71978	AAGCCCCCGGTTTTCCC
chr10	3479976	3480125	+	3480023	3480039	-28	-13	+	0.719774	GAGAGCGGACTGTTACT
chr10	3479926	3480075	+	3480023	3480039	22	37	+	0.719774	GAGAGCGGACTGTTACT
chr7	4843826	4843975	+	4843860	4843876	-41	-26	-	0.719772	CGGAAGAGTGCGTGCAT
chr14	104639676	104639825	+	104639702	104639718	-49	-34	+	0.719625	GAGAAGGCGGCTTTCTA
chr20	62004676	62004825	+	62004700	62004716	-51	-36	-	0.719422	CGGGACTGACTCTTCCT
chr20	62004626	62004775	+	62004700	62004716	-1	14	-	0.719422	CGGGACTGACTCTTCCT
chr7	30029826	30029975	+	30029863	30029879	-38	-23	-	0.719243	AGCCAGACCCTGTCCCA
chr7	30029776	30029925	+	30029863	30029879	12	27	-	0.719243	AGCCAGACCCTGTCCCA
chr22	18508226	18508375	+	18508262	18508278	-39	-24	-	0.718404	GGGCTCTCTCTGGTCCC
chr16	24697376	24697525	+	24697386	24697402	-65	-50	-	0.718384	CAGGACCTCTTGGACCT
chr16	24697326	24697475	+	24697386	24697402	-15	0	-	0.718384	CAGGACCTCTTGGACCT
chr1	21913376	21913525	+	21913418	21913434	-33	-18	+	0.718288	GAGTACAGGATTTTAAT
chr20	23969726	23969875	+	23969764	23969780	-37	-22	+	0.718285	AGTTCCAGGGCGTCCTC
chr7	23471726	23471875	+	23471789	23471805	-12	3	+	0.717889	TAGAACAGAGTCTGACA
chr7	23471676	23471825	+	23471789	23471805	38	53	+	0.717889	TAGAACAGAGTCTGACA
chr10	4230476	4230625	+	4230509	4230525	-42	-27	+	0.71772	AAGCATCCTGTGAACCC
chr10	4230426	4230575	+	4230509	4230525	8	23	+	0.71772	AAGCATCCTGTGAACCC
chr22	26877576	26877725	+	26877667	26877683	16	31	-	0.716807	TGGCAGGCCTTGTTACT
chr22	26877526	26877675	+	26877667	26877683	66	81	-	0.716807	TGGCAGGCCTTGTTACT
chr20	44934626	44934775	+	44934681	44934697	-20	-5	-	0.716715	GGCCAAACTTTGTCCTC
chr20	44934576	44934725	+	44934681	44934697	30	45	-	0.716715	GGCCAAACTTTGTCCTC
chr1	2527376	2527525	+	2527389	2527405	-62	-47	-	0.716309	CGGTGCTGCCCGTGCCC
chr1	2527326	2527475	+	2527389	2527405	-12	3	-	0.716309	CGGTGCTGCCCGTGCCC
chr3	64305676	64305825	+	64305708	64305724	-43	-28	-	0.715649	TAGAACTTTCCATACTT
chr3	64305626	64305775	+	64305708	64305724	7	22	-	0.715649	TAGAACTTTCCATACTT
chr14	93154626	93154775	+	93154699	93154715	-2	13	-	0.715579	CATTCCATGACGTGCCC

Supplementary Table S7 (con'd)

chr5	1010876	1011025	+	1011007	1011023	56	71	-	0.715578	CGCTGCACCATGAACCT
chr15	62358676	62358825	+	62358677	62358693	-74	-59	+	0.715567	AGACATGAAATGTTCCC
chr6	170585776	170585925	+	170585893	170585909	42	57	-	0.715446	TGCAACATACTGACCTT
chr19	17138726	17138875	+	17138740	17138756	-61	-46	-	0.715442	AGTCGCACTGTGTTGCC
chr9	129282626	129282775	+	129282661	129282677	-40	-25	-	0.715401	AACAAGACCAGGTACTT
chr9	129282576	129282725	+	129282661	129282677	10	25	-	0.715401	AACAAGACCAGGTACTT
chr9	139587826	139587975	+	139587868	139587884	-33	-18	-	0.715137	CTGGACACCCTCTGCCG
chr9	139587776	139587925	+	139587868	139587884	17	32	-	0.715137	CTGGACACCCTCTGCCG
chr14	70476726	70476875	+	70476858	70476874	57	72	+	0.71504	AGCAGCATTGTGTAATT
chr1	200175476	200175625	+	200175496	200175512	-55	-40	-	0.714357	GACAACACCTTATCCTA
chr17	27347076	27347225	+	27347145	27347161	-6	9	+	0.71427	GGGCCCGAGGTGAGCTT
chr17	77536126	77536275	+	77536263	77536279	62	77	+	0.714121	AAGAACATTCTGACTCG
chr15	33437276	33437425	+	33437376	33437392	25	40	-	0.713709	GGCTGCACAAGGTGCCT
chr14	101123276	101123425	+	101123308	101123324	-43	-28	-	0.713096	CAGCACCCGCTGTCACT
chr14	101123226	101123375	+	101123308	101123324	7	22	-	0.713096	CAGCACCCGCTGTCACT
chr11	118042626	118042775	+	118042666	118042682	-35	-20	+	0.713089	AGGGACAAGCTGAGGCC
chr5	2204476	2204625	+	2204561	2204577	10	25	+	0.713081	GGGCACAGTGGGAATCC
chr10	4358426	4358575	+	4358449	4358465	-52	-37	-	0.712507	AAACACATTATGTCTTT
chr10	3378776	3378925	+	3378813	3378829	-38	-23	-	0.712474	TTGCACAGCACGCACTT
chr16	67335976	67336125	+	67336007	67336023	-44	-29	-	0.712111	CATTACTTTGTTTTCCT
chr14	104851976	104852125	+	104852067	104852083	16	31	-	0.71207	TAGAACACCAGTCACTC
chr14	104851926	104852075	+	104852067	104852083	66	81	-	0.71207	TAGAACACCAGTCACTC
chr17	35277326	35277475	+	35277377	35277393	-24	-9	+	0.711936	GGGAGCAGGCAGGACCC
chr17	35277276	35277425	+	35277377	35277393	26	41	+	0.711936	GGGAGCAGGCAGGACCC
chr1	231761526	231761675	+	231761653	231761669	52	67	-	0.71168	AGGACCTCAGCCTTCCT
chr11	62100676	62100825	+	62100695	62100711	-56	-41	+	0.710832	GGAAAGGTGGTTTTCCC
chr11	62100626	62100775	+	62100695	62100711	-6	9	+	0.710832	GGAAAGGTGGTTTTCCC
chr11	62100576	62100725	+	62100695	62100711	44	59	+	0.710832	GGAAAGGTGGTTTTCCC
chr16	49530476	49530625	+	49530499	49530515	-52	-37	-	0.710661	AATAAAACTGTTTTCTT
chr3	54353576	54353725	+	54353643	54353659	-8	7	+	0.710601	AGTTAAACCGTGTTTTG
chr19	554876	555025	+	554944	554960	-7	8	-	0.710297	TGGCACGTCCTGGTGCT
chr19	554826	554975	+	554944	554960	43	58	-	0.710297	TGGCACGTCCTGGTGCT
chr14	106095426	106095575	+	106095533	106095549	32	47	-	0.710252	CATCCCGTCATGTTCCT
chr1	7130326	7130475	+	7130462	7130478	61	76	-	0.710251	AGTTACTTGTTTTTCCT
chr10	5406476	5406625	+	5406607	5406623	56	71	+	0.710223	AGGTCCAGGCTCTACAT
chr4	7652026	7652175	+	7652086	7652102	-15	0	-	0.709356	CAGAACGTGCTGTGTGC
chr8	144303226	144303375	+	144303297	144303313	-4	11	-	0.708927	AGTTTCATTTTGCTCCC
chr8	144303176	144303325	+	144303297	144303313	46	61	-	0.708927	AGTTTCATTTTGCTCCC
chr4	1537476	1537625	+	1537489	1537505	-62	-47	+	0.708447	ATGTGCACAATGTGCAG
chr1	200003226	200003375	+	200003308	200003324	7	22	-	0.708441	AGTTTCACAAGGTGCTT
chr4	3288826	3288975	+	3288926	3288942	25	40	-	0.708372	TTGTTCAAGGTGGACTC
chr17	81035976	81036125	+	81035996	81036012	-55	-40	+	0.708355	TGTCACTTGATGTGCTG
chr5	10653226	10653375	+	10653307	10653323	6	21	+	0.708336	GGGTCCAGGCAGCTCCT
chr5	10653176	10653325	+	10653307	10653323	56	71	+	0.708336	GGGTCCAGGCAGCTCCT
chr10	3250676	3250825	+	3250793	3250809	42	57	-	0.707985	GGGTGCGCTGCTTCCTC
chr8	25902126	25902275	+	25902156	25902172	-45	-30	+	0.707899	AGGCACGGGGTGCGCGG
chr10	94448476	94448625	+	94448587	94448603	36	51	+	0.707897	TTGGACAAATAGTTCCA
chr1	21044826	21044975	+	21044850	21044866	-51	-36	+	0.707717	AAGGACCTGTTGCCCCT
chr19	17571526	17571675	+	17571661	17571677	60	75	+	0.707689	GAGAACAGGGAGGCCAT
chr7	73465976	73466125	+	73466112	73466128	61	76	+	0.707666	GAGGGGCTGGTGTTCCT
chr6	168533376	168533525	+	168533434	168533450	-17	-2	+	0.707438	AAGATCAGCGCGTCCAG
chr5	114514576	114514725	+	114514686	114514702	35	50	+	0.707295	GGCTACAGAATCATCCT
chr1	7601876	7602025	+	7601897	7601913	-54	-39	+	0.706944	GGGCTCAGAATGCGCTG
chr1	15655776	15655925	+	15655812	15655828	-39	-24	+	0.706872	GGGCTCCTCATGATCTC
chr14	104617876	104618025	+	104617905	104617921	-46	-31	+	0.706808	AGGGAGAGGGTCTCCTG
chr14	104617826	104617975	+	104617905	104617921	4	19	+	0.706808	AGGGAGAGGGTCTCCTG
chr12	124607826	124607975	+	124607931	124607947	30	45	+	0.706445	ATGAACCCTTTATTCCA
chr3	14595776	14595925	+	14595898	14595914	47	62	-	0.706408	AGGATCCCACTGGGCCC
chr9	122800776	122800925	+	122800802	122800818	-49	-34	+	0.70631	GGCAGCATGCTCTTCCT
chr7	4678576	4678725	+	4678709	4678725	58	73	-	0.706007	AGGAATATAAGGTCCAC
chr10	4445976	4446125	+	4446009	4446025	-42	-27	+	0.705995	GAGGCCAGAGTATCCTC
chr7	1686926	1687075	+	1686990	1687006	-11	4	-	0.705945	TTGGACGCGCTGTGGCC
chr15	100016226	100016375	+	100016303	100016319	2	17	-	0.705671	GGGAACATCTTCCTTCT
chr10	103326626	103326775	+	103326724	103326740	23	38	-	0.705632	AGGCACAACCCTGACCT
chr1	1097226	1097375	+	1097349	1097365	48	63	-	0.705624	AAGCAAACACTTTGCCA
chr12	49366076	49366225	+	49366080	49366096	-71	-56	-	0.705479	GGGGACAGCTTTGGCCT
chr1	1957676	1957825	+	1957766	1957782	15	30	-	0.705206	AGGGGCGCCTTCTTCTT
chr14	106229476	106229625	+	106229559	106229575	8	23	-	0.704909	GAGAAAGCTCCGTGCAC
chr17	79109526	79109675	+	79109556	79109572	-45	-30	+	0.704829	GTCTCCAGGTTGTTCCC
chr5	74532226	74532375	+	74532299	74532315	-2	13	+	0.704054	AAGCACATGTTCTGGTC
chr21	46973126	46973275	+	46973134	46973150	-67	-52	-	0.703812	AGGCACCGCGCATCCCT
chr5	140710276	140710425	+	140710335	140710351	-16	-1	+	0.703637	GGGAATATTCACTACTC
chr4	187071076	187071225	+	187071199	187071215	48	63	+	0.703198	AAGTAAAAGCTGTGGCA
chr14	94451326	94451475	+	94451407	94451423	6	21	+	0.702249	GAGAACTGAGTGGATTT
chr12	44857976	44858125	+	44857988	44858004	-63	-48	+	0.702243	AGGATCAAAATGTAAAA
chr12	44857926	44858075	+	44857988	44858004	-13	2	+	0.702243	AGGATCAAAATGTAAAA
chr7	158800526	158800675	+	158800654	158800670	53	68	+	0.702034	GAAAAGAGGGTGGACCA
chr15	102094576	102094725	+	102094683	102094699	32	47	-	0.702032	TGTTAGGTTGCGTACTT
chr19	3820976	3821125	+	3821076	3821092	25	40	-	0.702014	TGGCCGGCAGTGTCCCT
chr3	129326676	129326825	+	129326757	129326773	6	21	-	0.701989	TGCCAGACACTGTGCCA
chr3	129326626	129326775	+	129326757	129326773	56	71	-	0.701989	TGCCAGACACTGTGCCA
chr22	40051426	40051575	+	40051538	40051554	37	52	-	0.701972	CCGTACCTATGGTTCCC
chr7	30717926	30718075	+	30718047	30718063	46	61	+	0.701661	AGGAGACTCGTGTGCTT
chr8	94508376	94508525	+	94508413	94508429	-38	-23	+	0.701616	GGGCAAGCAGAGCACCC
chr6	168629826	168629975	+	168629886	168629902	-15	0	-	0.701485	AGGCTCACCCGCTTCTT
chr6	168629776	168629925	+	168629886	168629902	35	50	-	0.701485	AGGCTCACCCGCTTCTT
chr14	106174276	106174425	+	106174345	106174361	-6	9	-	0.70148	GTGCACGTGAGGTTCGC
chr1	7601926	7602075	+	7602020	7602036	19	34	+	0.701279	CAGAACCAAGCGTTACA
chr1	1974726	1974875	+	1974817	1974833	16	31	-	0.701166	TGGCAGGGCGTGTCCGC
chr1	1974676	1974825	+	1974817	1974833	66	81	-	0.701166	TGGCAGGGCGTGTCCGC
chr17	14206926	14207075	+	14206996	14207012	-5	10	-	0.701148	AGGCACGTGAGGGCCTC
chr17	14206876	14207025	+	14206996	14207012	45	60	-	0.701148	AGGCACGTGAGGGCCTC
chr6	168617376	168617525	+	168617434	168617450	-17	-2	+	0.701095	GAGCCCAGACTGCTCTG
chr6	168617326	168617475	+	168617434	168617450	33	48	+	0.701095	GAGCCCAGACTGCTCTG
chr15	75019226	75019375	+	75019256	75019272	-45	-30	-	0.70102	TAGCTCAACCTGGCCCC
chr16	1316076	1316225	+	1316101	1316117	-50	-35	-	0.700756	GAGGACCCCTCGAGCCC
chr17	60214576	60214725	+	60214664	60214680	13	28	+	0.700195	GGGAAGCCGTTCTTCTG
chr17	60214526	60214675	+	60214664	60214680	63	78	+	0.700195	GGGAAGCCGTTCTTCTG
chr15	66543826	66543975	+	66543881	66543897	-20	-5	-	0.699893	CAGCACAGCACCTAACC
chr1	7539026	7539175	+	7539035	7539051	-66	-51	+	0.699635	GGGCAAAGAGTGAAATC
chr8	1923026	1923175	+	1923109	1923125	8	23	-	0.699604	AAGTTCGCCCGATTCCC

Supplementary Table S7 (con’d)

chr8	1922976	1923125	+	1923109	1923125	58	73	-	0.699604	AAGTTCGCCCGATTCCC
chr17	34995976	34996125	+	34996056	34996072	5	20	-	0.699588	GAGAGCAGCCGGCTCCC
chr8	22018376	22018525	+	22018453	22018469	2	17	+	0.699487	GGGTAGAGTGTAGACCA
chr15	102094526	102094675	+	102094628	102094644	27	42	+	0.699484	TGGAACCGGACGTGGTT
chr16	30034726	30034875	+	30034769	30034785	-32	-17	+	0.699298	GAGCATAAAGTGTGATG
chr22	37215826	37215975	+	37215862	37215878	-39	-24	+	0.698587	AGGAAAAGAATGAAACA
chr10	2978726	2978875	+	2978767	2978783	-34	-19	+	0.698239	TCGTATGAAGTGTGCTT
chr1	38517126	38517275	+	38517155	38517171	-46	-31	+	0.698002	GGCTCCAGGATGCTCCA
chr11	72973976	72974125	+	72973987	72974003	-64	-49	+	0.697991	GGGAAGAGTGTGAAGTG
chr7	3488726	3488875	+	3488745	3488761	-56	-41	-	0.697775	GGATTCATTGAGTCCTT
chr5	140710426	140710575	+	140710544	140710560	43	58	+	0.697644	GAGCATGCCGTGTCTCG
chr3	139258226	139258375	+	139258348	139258364	47	62	-	0.697486	CAGTACCCAGTGAAGTC
chr14	105052376	105052525	+	105052478	105052494	27	42	-	0.697426	TGGAATATTATGCAGCC
chr3	66139526	66139675	+	66139640	66139656	39	54	+	0.697327	AGGAAAATAAGATTCCG
chr11	4842976	4843125	+	4843047	4843063	-4	11	-	0.697275	AGATACACTACCATCCT
chr16	85201726	85201875	+	85201794	85201810	-7	8	+	0.696706	TGGAGCCCGACGTCCTG
chr15	78186426	78186575	+	78186535	78186551	34	49	+	0.696679	GAGCACAGCAGCTGCTG
chr15	29611876	29612025	+	29611903	29611919	-48	-33	-	0.696666	CAGAACAAACTGCCACT
chr8	143546776	143546925	+	143546876	143546892	25	40	-	0.696452	GGGTTGAGTATGCTCTC
chr7	73790676	73790825	+	73790750	73790766	-1	14	-	0.696219	GAGACCGCCATGCACGT
chr2	242151476	242151625	+	242151594	242151610	43	58	-	0.695944	GTCTACATTGCCTTCTT
chr21	44724626	44724775	+	44724649	44724665	-52	-37	-	0.695935	GGCCACACCGTTGCCCC
chr7	5319476	5319625	+	5319610	5319626	59	74	-	0.695828	CCGAAATTACTGTACCT
chr19	45003726	45003875	+	45003825	45003841	24	39	+	0.69574	AAGCGCGTTGTCTGCTC
chr5	2205876	2206025	+	2205908	2205924	-43	-28	-	0.695739	CTGTACACACTGCGGCC
chr5	2205826	2205975	+	2205908	2205924	7	22	-	0.695739	CTGTACACACTGCGGCC
chr1	6531076	6531225	+	6531189	6531205	38	53	-	0.695638	GGTTACCGCGCGCTCCC
chr7	158818076	158818225	+	158818153	158818169	2	17	-	0.695415	GACCACCACACGTGCCC
chr7	1328926	1329075	+	1328962	1328978	-39	-24	-	0.695398	TAGCAGAAGCTGTGCGT
chr7	120967776	120967925	+	120967866	120967882	15	30	-	0.695041	CTAAACACGTTGCACTT
chr10	3280976	3281125	+	3281056	3281072	5	20	+	0.694725	GGGCACAGAGGGAATCG
chr5	73969076	73969225	+	73969189	73969205	38	53	+	0.694106	TAGAAAAATGTGATCAT
chr10	4358476	4358625	+	4358573	4358589	22	37	-	0.693686	AGACTCTCTTTGTTCTC
chr10	134303826	134303975	+	134303916	134303932	15	30	+	0.693092	GGCCACCAGCTGTTCAT
chr7	1684526	1684675	+	1684554	1684570	-47	-32	-	0.692822	GGGAGAGGCCTGTCCTC
chr6	168629726	168629875	+	168629781	168629797	-20	-5	-	0.692428	AATTTCGGTCTGTTCCG
chr10	3602676	3602825	+	3602712	3602728	-39	-24	+	0.692333	AGGTAAAGACACTACCT
chr10	3602626	3602775	+	3602712	3602728	11	26	+	0.692333	AGGTAAAGACACTACCT
chr21	41027776	41027925	+	41027875	41027891	24	39	+	0.692166	GTGGGCACAGTGGTCTG
chr7	1407276	1407425	+	1407403	1407419	52	67	-	0.692136	GATTTCATTTTGCACCT
chr1	229480026	229480175	+	229480127	229480143	26	41	-	0.691929	GGTCAGACCGTCTGCTC
chr16	474226	474375	+	474327	474343	26	41	-	0.691607	GAATGCAGTATCTTCCT
chr1	1936476	1936625	+	1936508	1936524	-43	-28	-	0.691354	CTGTGCATCCTGTTACA
chr1	1936426	1936575	+	1936508	1936524	7	22	-	0.691354	CTGTGCATCCTGTTACA
chr1	1936376	1936525	+	1936508	1936524	57	72	-	0.691354	CTGTGCATCCTGTTACA
chr5	1207326	1207475	+	1207371	1207387	-30	-15	+	0.69113	GAGCACCTTGGGCACCG
chr10	3708426	3708575	+	3708440	3708456	-61	-46	-	0.691057	CAAAACACACAGTTGCT
chr10	3708376	3708525	+	3708440	3708456	-11	4	-	0.691057	CAAAACACACAGTTGCT
chr10	3708326	3708475	+	3708440	3708456	39	54	-	0.691057	CAAAACACACAGTTGCT
chr14	100631676	100631825	+	100631705	100631721	-46	-31	-	0.691	CAGACCCCAGCGTTCGC
chr9	6716276	6716425	+	6716374	6716390	23	38	+	0.690858	ACGATCGCTGGGTTCTA
chr6	33561276	33561425	+	33561278	33561294	-73	-58	-	0.690722	CAGATGATGATGTTGCT
chr20	61979276	61979425	+	61979283	61979299	-68	-53	+	0.690628	AGGCAAACGGGGTCACT
chr20	61979226	61979375	+	61979283	61979299	-18	-3	+	0.690628	AGGCAAACGGGGTCACT
chr19	4566426	4566575	+	4566500	4566516	-1	14	-	0.6906	AAGACCATCTGCTTCTC
chr14	70348376	70348525	+	70348445	70348461	-6	9	+	0.690273	GAGAAGGTGCTGTCTCG
chr14	70348326	70348475	+	70348445	70348461	44	59	+	0.690273	GAGAAGGTGCTGTCTCG
chr2	219487476	219487625	+	219487574	219487590	23	38	-	0.690107	TTGACCGCTATGAACCT
chr7	131831476	131831625	+	131831507	131831523	-44	-29	-	0.690057	TAGCCCATCACTTACCA
chr2	219487426	219487575	+	219487565	219487581	64	79	-	0.689272	TGGAACTCATTGACCGC
chr12	116944026	116944175	+	116944163	116944179	62	77	+	0.68912	GGGAACAGACTGAGGAC
chr11	64739726	64739875	+	64739777	64739793	-24	-9	-	0.688978	CGTTATGCAGGGTACCT
chr11	64739676	64739825	+	64739777	64739793	26	41	-	0.688978	CGTTATGCAGGGTACCT
chr10	4194426	4194575	+	4194499	4194515	-2	13	-	0.688912	GTGACAACCACGTCCCT
chr7	72787926	72788075	+	72787951	72787967	-50	-35	-	0.688623	CAGAACTACCCCTTCCG
chr11	67219426	67219575	+	67219531	67219547	30	45	-	0.688505	TGCGACAGGCTGTGGCC
chr1	1936526	1936675	+	1936610	1936626	9	24	-	0.688453	CGGGCCACTCTATGCCA
chr7	1686576	1686725	+	1686582	1686598	-69	-54	-	0.688256	AAGTTCAGAGGGCTCCA
chr17	77644426	77644575	+	77644564	77644580	63	78	+	0.688047	CAGCACCCGGCCTGCCA
chr6	112132876	112133025	+	112132902	112132918	-49	-34	+	0.687475	ACCTACAAGTTGTAGCC
chr6	112132826	112132975	+	112132902	112132918	1	16	+	0.687475	ACCTACAAGTTGTAGCC
chr19	17571476	17571625	+	17571571	17571587	20	35	-	0.687453	AGCACCAGCCGGTTCTC
chr10	28971126	28971275	+	28971207	28971223	6	21	+	0.686807	GGGCACGAGAACTGCTG
chr11	2293126	2293275	+	2293164	2293180	-37	-22	+	0.686672	GGGAAACGCGTGCCCTC
chr4	24796526	24796675	+	24796539	24796555	-62	-47	+	0.686621	GAGTGCGGCTAGTGCCA
chr4	24796476	24796625	+	24796539	24796555	-12	3	+	0.686621	GAGTGCGGCTAGTGCCA
chr5	131350026	131350175	+	131350094	131350110	-7	8	+	0.686429	AAGAAAGTCATGCACAT
chr11	2293176	2293325	+	2293214	2293230	-37	-22	+	0.686315	GGTGACCAGATGCTCCT
chr12	322076	322225	+	322111	322127	-40	-25	-	0.686276	AAACACACCAATTTCCA
chr16	474176	474325	+	474178	474194	-73	-58	+	0.686071	GGGAGCCTGCTGTCTCT
chr16	474126	474275	+	474178	474194	-23	-8	+	0.686071	GGGAGCCTGCTGTCTCT
chr14	104639726	104639875	+	104639786	104639802	-15	0	+	0.686006	TCCCACATGTTGTTCAC
chr1	17022476	17022625	+	17022597	17022613	46	61	+	0.685924	GCCAACATGGTGAAACC
chr14	100631726	100631875	+	100631766	100631782	-35	-20	+	0.685514	GAGAACTGTGAGTGACA
chr2	239048526	239048675	+	239048656	239048672	55	70	+	0.68505	TGGAATGCAGGGTTCAG
chr17	1811226	1811375	+	1811272	1811288	-29	-14	+	0.684835	AGGCAGAGCCTGCGCTG
chr11	2190026	2190175	+	2190070	2190086	-31	-16	+	0.684706	CAGGCCAGGGTTTGCCA
chr6	37502976	37503125	+	37503034	37503050	-17	-2	-	0.684464	AGGAAGGCTGGGTGTCT
chr17	66288726	66288875	+	66288841	66288857	40	55	-	0.684394	TTGCTCGCTGAGTTCTC
chr6	134350826	134350975	+	134350906	134350922	5	20	-	0.684385	TTAAACATTTTGTATTT
chr6	134350776	134350925	+	134350906	134350922	55	70	-	0.684385	TTAAACATTTTGTATTT
chr20	47278426	47278575	+	47278509	47278525	8	23	-	0.684303	AGGGAAACCAGCTGCCT
chr6	156954426	156954575	+	156954565	156954581	64	79	+	0.68425	GCGGACGATCTGAACTG
chr17	78456326	78456475	+	78456370	78456386	-31	-16	-	0.684232	TAAAACACCATTCTCCG
chr5	2537676	2537825	+	2537747	2537763	-4	11	-	0.684051	TGTGGCTCAGTGTTCTT
chr7	3019076	3019225	+	3019173	3019189	22	37	+	0.684047	AGAGACACTAAGAGCCT
chr11	2206026	2206175	+	2206140	2206156	39	54	-	0.684016	AGGTCCGCTTGCTTCCC
chr8	136510476	136510625	+	136510585	136510601	34	49	+	0.683949	GTGGCCAGAATGATCTT
chr5	132944026	132944175	+	132944090	132944106	-11	4	+	0.683858	GAGAACCTGGTGCTAAC
chr7	1358126	1358275	+	1358225	1358241	24	39	-	0.683401	ATGCACAAATGGTCCTA
chr16	22776026	22776175	+	22776106	22776122	5	20	-	0.683386	TTGTCCAGGATGAGCCC

Supplementary Table S7 (con'd)

chr16	22775976	22776125	+	22776106	22776122	55	70	-	0.683386	TTGTCCAGGATGAGCCC
chr7	44279526	44279675	+	44279664	44279680	63	78	+	0.683186	TCGCACACGGAGTGTCG
chr14	104770726	104770875	+	104770766	104770782	-35	-20	+	0.683159	AGAAACCCTCTATTCTG
chr3	186170626	186170775	+	186170627	186170643	-74	-59	-	0.682855	CGACACTCAGTCTCCCC
chr15	75019276	75019425	+	75019282	75019298	-69	-54	-	0.682837	CGTTACGCGCCATCCCC
chr1	203525526	203525675	+	203525609	203525625	8	23	+	0.682721	GAGAACACATTTCATTT
chr16	281326	281475	+	281436	281452	35	50	+	0.682609	AAGAAGGCTATGATTCT
chr10	1602426	1602575	+	1602481	1602497	-20	-5	-	0.68258	AGAAACATTATTTGATC
chr17	75789476	75789625	+	75789553	75789569	2	17	+	0.682472	GGGAATGTGCTCTACAA
chr8	143545076	143545225	+	143545099	143545115	-52	-37	-	0.682434	AGGTACTTTGTTCCCCA
chr5	1855976	1856125	+	1856111	1856127	60	75	+	0.681871	TGCAATACAATTTACCA
chr5	2335526	2335675	+	2335659	2335675	58	73	-	0.68162	TGCTATGCATTGTGCCA
chr3	13058826	13058975	+	13058862	13058878	-39	-24	-	0.681443	GCAAACAATAGGTGCTC
chr3	13058776	13058925	+	13058862	13058878	11	26	-	0.681443	GCAAACAATAGGTGCTC
chr11	67619726	67619875	+	67619738	67619754	-63	-48	-	0.68131	GTGAAGGGCTGGTACCC
chr10	3544576	3544725	+	3544585	3544601	-66	-51	-	0.681235	GTGCTCACTGGGTGCTG
chr10	3544526	3544675	+	3544585	3544601	-16	-1	-	0.681235	GTGCTCACTGGGTGCTG
chr1	57718876	57719025	+	57718902	57718918	-49	-34	-	0.681003	AGGGTAGCACTGTCCTC
chr22	37500626	37500775	+	37500685	37500701	-16	-1	-	0.680882	ATAAATACCGTGTAGCT
chr22	37500576	37500725	+	37500685	37500701	34	49	-	0.680882	ATAAATACCGTGTAGCT
chr22	37771126	37771275	+	37771131	37771147	-70	-55	+	0.680871	GAGAAGGCGGTGGGCGT
chr5	173097476	173097625	+	173097518	173097534	-33	-18	-	0.680813	TAAAAAAACATGAACCC
chr5	173097426	173097575	+	173097518	173097534	17	32	-	0.680813	TAAAAAAACATGAACCC
chr10	4386726	4386875	+	4386865	4386881	64	79	+	0.680799	TGGAAAACATTCTTCAG
chr8	143570826	143570975	+	143570833	143570849	-68	-53	-	0.680575	CAGAAACGCGTGTGCGC
chr14	106438026	106438175	+	106438119	106438135	18	33	+	0.680331	GAGGAGGCATAGTGCCA
chr12	116008026	116008175	+	116008084	116008100	-17	-2	+	0.680142	TAGGACAATTAGCACCA
chr12	116007976	116008125	+	116008084	116008100	33	48	+	0.680142	TAGGACAATTAGCACCA
chr8	1922926	1923075	+	1922954	1922970	-47	-32	-	0.679927	TACAAAACCACGTTTCC
chr2	3697426	3697575	+	3697467	3697483	-34	-19	-	0.6798	TGGGATTCAGTGTTTTT
chr7	2728826	2728975	+	2728895	2728911	-6	9	+	0.679752	GGGGAGCATGAGTGCCC
chr7	2728776	2728925	+	2728895	2728911	44	59	+	0.679752	GGGGAGCATGAGTGCCC
chr15	33437226	33437375	+	33437313	33437329	12	27	+	0.679658	AGAAATCATGTGTGCTC
chr19	15585376	15585525	+	15585503	15585519	52	67	-	0.679548	AATGACATAATGTTGGC
chr7	99987526	99987675	+	99987601	99987617	0	15	+	0.679427	GGGCAAACGACGCTCAG
chr7	99987476	99987625	+	99987601	99987617	50	65	+	0.679427	GGGCAAACGACGCTCAG
chr2	239695676	239695825	+	239695686	239695702	-65	-50	-	0.679296	CACCGCACACTGTGCCG
chr19	3030226	3030375	+	3030257	3030273	-44	-29	+	0.679205	CAGAACTGGAAGAACCA
chr19	3030176	3030325	+	3030257	3030273	6	21	+	0.679205	CAGAACTGGAAGAACCA
chr1	22975576	22975725	+	22975606	22975622	-45	-30	+	0.678915	GAGAACCTGGCCTGTCC
chr1	22975526	22975675	+	22975606	22975622	5	20	+	0.678915	GAGAACCTGGCCTGTCC
chr21	46799776	46799925	+	46799802	46799818	-49	-34	-	0.678868	CAGAACAGAAGCTCCTG
chr9	139925726	139925875	+	139925734	139925750	-67	-52	+	0.678573	GGGCACAAAGATGTCCG
chr8	144212926	144213075	+	144212976	144212992	-25	-10	+	0.678228	AAGACCGTGATGGAACC
chr7	73465926	73466075	+	73465953	73465969	-48	-33	-	0.678202	GGAGACACCCGGGCCCC
chr7	73465876	73466025	+	73465953	73465969	2	17	-	0.678202	GGAGACACCCGGGCCCC
chr17	25798626	25798775	+	25798691	25798707	-10	5	+	0.678137	AGCAAACAGGTGTCCTC
chr11	120044426	120044575	+	120044512	120044528	11	26	+	0.678075	GGCTACACAGCTAACTG
chr7	95154926	95155075	+	95154932	95154948	-69	-54	-	0.678028	AATTTCATCCTGACCCT
chr4	8158176	8158325	+	8158217	8158233	-34	-19	-	0.677961	GGCAACTGCATGGCCTC
chr1	172291626	172291775	+	172291667	172291683	-34	-19	-	0.677959	AGGAGAGCCATTTGCCC
chr1	172291576	172291725	+	172291667	172291683	16	31	-	0.677959	AGGAGAGCCATTTGCCC
chr14	70037526	70037675	+	70037545	70037561	-56	-41	-	0.677541	TTGAACATTATCTGCAG
chr5	140710376	140710525	+	140710438	140710454	-13	2	-	0.677265	GAGTCCGCATCGTCTCC
chr2	27938026	27938175	+	27938093	27938109	-8	7	+	0.67726	GGGGACAGGATGCAAGG
chr9	34588426	34588575	+	34588504	34588520	3	18	-	0.677253	CAGAACAACTAGTGTTA
chr14	101123376	101123525	+	101123382	101123398	-69	-54	+	0.67721	ATGCACCCGGTTTCCTG
chr8	143535526	143535675	+	143535527	143535543	-74	-59	+	0.67715	GTGCACAGCCTGTTGAG
chr8	143535426	143535575	+	143535527	143535543	26	41	+	0.67715	GTGCACAGCCTGTTGAG
chr3	139258176	139258325	+	139258312	139258328	61	76	-	0.676829	AGGTACTCCTCGAAATT
chr16	90115026	90115175	+	90115118	90115134	17	32	+	0.676344	GGCAACGTATTTTCCTA
chr16	876176	876325	+	876245	876261	-6	9	-	0.676249	TGGTCGCCACTGTGCCT
chr16	876126	876275	+	876245	876261	44	59	-	0.676249	TGGTCGCCACTGTGCCT
chr17	78981976	78982125	+	78982021	78982037	-30	-15	+	0.675992	AGGAACAGGACCCTTCC
chr17	78981926	78982075	+	78982021	78982037	20	35	+	0.675992	AGGAACAGGACCCTTCC
chr4	3288326	3288475	+	3288455	3288471	54	69	+	0.675962	AGCGGGAACGTGTTCCC
chr1	22889176	22889325	+	22889262	22889278	11	26	-	0.675927	GAGTTCCACCTGGGCCC
chr1	1920726	1920875	+	1920767	1920783	-34	-19	+	0.675885	CAGGACAGGGCGGACGG
chr1	1920676	1920825	+	1920767	1920783	16	31	+	0.675885	CAGGACAGGGCGGACGG
chr1	1920626	1920775	+	1920767	1920783	66	81	+	0.675885	CAGGACAGGGCGGACGG
chr10	130959526	130959675	+	130959550	130959566	-51	-36	-	0.675819	TGGAATGTCCCTTCCCC
chr6	46455876	46456025	+	46455959	46455975	8	23	+	0.675775	AGGTGCACGTCTTACAA
chr6	46455826	46455975	+	46455959	46455975	58	73	+	0.675775	AGGTGCACGTCTTACAA
chr5	111089976	111090125	+	111089992	111090008	-59	-44	+	0.675766	AGAAACATGGTATCTTA
chr19	44146826	44146975	+	44146860	44146876	-41	-26	-	0.675658	AATAACACCGTTTTTGC
chr7	1686676	1686825	+	1686730	1686746	-21	-6	-	0.675447	CTGCACGGGCTGACCCC
chr7	1686626	1686775	+	1686730	1686746	29	44	-	0.675447	CTGCACGGGCTGACCCC
chr1	61407976	61408125	+	61408020	61408036	-31	-16	-	0.675395	TGGTGCAATTGTTTCCC
chr2	159705326	159705475	+	159705392	159705408	-9	6	+	0.675326	AAGGTCCAGCCGTTCCA
chr2	159705276	159705425	+	159705392	159705408	41	56	+	0.675326	AAGGTCCAGCCGTTCCA
chr5	141993126	141993275	+	141993133	141993149	-68	-53	-	0.675216	CTGTGCAGGATTTACCA
chr10	5406926	5407075	+	5406970	5406986	-31	-16	-	0.67497	CGATCCACCCTGTGATC
chr2	75136476	75136625	+	75136495	75136511	-56	-41	+	0.674853	GCGACCGGCGGGTTCTG
chr15	78114776	78114925	+	78114837	78114853	-14	1	-	0.674674	GGGGTCCCCTTGTGTCC
chr2	189191626	189191775	+	189191632	189191648	-69	-54	+	0.674577	GCAGACAGTCTGTACAA
chr2	189191576	189191725	+	189191632	189191648	-19	-4	+	0.674577	GCAGACAGTCTGTACAA
chr2	189191526	189191675	+	189191632	189191648	31	46	+	0.674577	GCAGACAGTCTGTACAA
chr15	62543076	62543225	+	62543089	62543105	-62	-47	-	0.674494	GAGTGAGCCTTTTTCCC
chr15	62543026	62543175	+	62543089	62543105	-12	3	-	0.674494	GAGTGAGCCTTTTTCCC
chr17	41739176	41739325	+	41739249	41739265	-2	13	-	0.674353	GAGTTCGTCCTGGAGCC
chr17	41739126	41739275	+	41739249	41739265	48	63	-	0.674353	GAGTTCGTCCTGGAGCC
chr5	497426	497575	+	497565	497581	64	79	+	0.674148	GGGCACCGGGACTGCTG
chr12	52238926	52239075	+	52239009	52239025	8	23	-	0.674123	AAGTACACTCCCCATCC
chr12	52238876	52239025	+	52239009	52239025	58	73	-	0.674123	AAGTACACTCCCCATCC
chr12	1675826	1675975	+	1675924	1675940	23	38	-	0.674009	AGCTTCCCCGAGTTCTC
chr11	60482376	60482525	+	60482401	60482417	-50	-35	+	0.673979	AGCCACAAAGAGTCCAT
chr7	24328476	24328625	+	24328571	24328587	20	35	+	0.673955	AAGCAGGGGTTGTTTTA
chr14	104627776	104627925	+	104627840	104627856	-11	4	-	0.673915	AAGGAAAATATTTCCTT
chr14	103691426	103691575	+	103691529	103691545	28	43	+	0.673854	AGTTACAGTCAGAGCCC
chr5	2206976	2207125	+	2207006	2207022	-45	-30	+	0.673832	AAGAACACATTTCAACA
chr5	2206926	2207075	+	2207006	2207022	5	20	+	0.673832	AAGAACACATTTCAACA

chr10	134610376	134610525	+	134610435	134610451	-16	-1	-	0.673802	TAAACCTTTCTGTTCTT
chr10	134610326	134610475	+	134610435	134610451	34	49	-	0.673802	TAAACCTTTCTGTTCTT
chr20	21483826	21483975	+	21483902	21483918	1	16	+	0.673781	TGGGAAATGGCGAACAT
chr16	29267726	29267875	+	29267866	29267882	65	80	+	0.673646	AGGAACCTGCCACTCCT
chr21	46799826	46799975	+	46799835	46799851	-66	-51	+	0.673612	GGCCTCACCGTGACCCA
chr1	154843126	154843275	+	154843214	154843230	13	28	+	0.673493	GTGGGCAGTATGGTCTC
chr6	37014426	37014575	+	37014460	37014476	-41	-26	+	0.673435	AAAGACAGGATGTTTAA
chr14	94463676	94463825	+	94463810	94463826	59	74	+	0.673321	AGGAACAGTTTGCAAGA
chr1	226791376	226791525	+	226791503	226791519	52	67	-	0.673257	TGTCGCGTCCTGTCCTC
chr19	47735926	47736075	+	47735928	47735944	-73	-58	-	0.672475	TGGTGAGTTTTGTCCTT
chr19	47735876	47736025	+	47735928	47735944	-23	-8	-	0.672475	TGGTGAGTTTTGTCCTT
chr5	2207076	2207225	+	2207204	2207220	53	68	+	0.672187	TGGTGCCTGAAGTCCTT
chr1	38513526	38513675	+	38513565	38513581	-36	-21	+	0.672174	AGGAGAAGGATGGGCTC
chr1	38513476	38513625	+	38513565	38513581	14	29	+	0.672174	AGGAGAAGGATGGGCTC
chr10	130959626	130959775	+	130959725	130959741	24	39	-	0.671597	CACCAGGCACTGTGCCT
chr10	3250626	3250775	+	3250719	3250735	18	33	-	0.671355	CAGGTCTTCCTTTTCCC
chr14	101927926	101928075	+	101928046	101928062	45	60	-	0.671275	TAGAATTTCCTGGCCCC
chr11	2293226	2293375	+	2293254	2293270	-47	-32	+	0.671055	GGGAACGGAGATTTTTG
chr1	121260626	121260775	+	121260694	121260710	-7	8	+	0.670996	AGGAGGAAAGTGTCTCT
chr15	67841326	67841475	+	67841368	67841384	-33	-18	-	0.670847	GGAAAGGCTGTGGGCTC
chr15	67841276	67841425	+	67841368	67841384	17	32	-	0.670847	GGAAAGGCTGTGGGCTC
chr17	27396876	27397025	+	27396986	27397002	35	50	+	0.670678	AGGACCTCGAGGTTCAA
chr20	4705176	4705325	+	4705299	4705315	48	63	+	0.670295	TGGAACCGGAAGGCCCT
chr8	54164676	54164825	+	54164685	54164701	-66	-51	-	0.670201	TATTTCCTTGCGTTCTC
chr8	54164626	54164775	+	54164685	54164701	-16	-1	-	0.670201	TATTTCCTTGCGTTCTC
chr10	3526676	3526825	+	3526786	3526802	35	50	+	0.670174	GAGCACACATTTATGCT
chr8	144303126	144303275	+	144303158	144303174	-43	-28	+	0.670135	AGGGGCATCCAGCACTC
chr17	27396826	27396975	+	27396909	27396925	8	23	-	0.670076	GAGCTCTCGGTTTCCCA
chr1	2527426	2527575	+	2527559	2527575	58	73	+	0.66983	ATGGCCCTCGTGTCCCA
chr10	4482576	4482725	+	4482694	4482710	43	58	+	0.669602	AGGTATCCCGTGATGCT
chr16	88963626	88963775	+	88963731	88963747	30	45	+	0.669477	TAGAGCCGGGAGTGCCA
chr10	134610276	134610425	+	134610276	134610292	-75	-60	+	0.669223	AGGTAACTTCTGAGCCA
chr10	134610226	134610375	+	134610276	134610292	-25	-10	+	0.669223	AGGTAACTTCTGAGCCA
chr16	1316326	1316475	+	1316454	1316470	53	68	-	0.669176	TTGAACTGCATTTGCCG
chr9	132315726	132315875	+	132315858	132315874	57	72	-	0.668873	AAGCATATCCTTATCCT
chr8	143546726	143546875	+	143546772	143546788	-29	-14	-	0.668859	GGGGACTGCAGGAGCCT
chr7	1407426	1407575	+	1407532	1407548	31	46	-	0.668768	ATAAACACTGACTGCCC
chr12	322176	322325	+	322181	322197	-70	-55	-	0.668582	AAGTTAAGTCTGTTTTC
chr12	322126	322275	+	322181	322197	-20	-5	-	0.668582	AAGTTAAGTCTGTTTTC
chr1	7408726	7408875	+	7408759	7408775	-42	-27	+	0.668545	AGCCACAGGGGGTGTTC
chr1	65362376	65362525	+	65362408	65362424	-43	-28	-	0.668476	CAGGACACACCCGACCA
chr1	4794826	4794975	+	4794886	4794902	-15	0	-	0.668445	GGAAGCATCTTCTGCTT
chr20	61371426	61371575	+	61371505	61371521	4	19	-	0.66813	CAAAGCGCTCTGTGCTA
chr14	37075376	37075525	+	37075475	37075491	24	39	+	0.668023	AGCCAAGCTGTGATCCA
chr7	3018376	3018525	+	3018397	3018413	-54	-39	-	0.667967	CGGCTCACCTGTTTCCA
chr7	3018326	3018475	+	3018397	3018413	-4	11	-	0.667967	CGGCTCACCTGTTTCCA
chr7	3018276	3018425	+	3018397	3018413	46	61	-	0.667967	CGGCTCACCTGTTTCCA
chr8	143868076	143868225	+	143868179	143868195	28	43	-	0.667854	CTGGCCCTGCTGTTCCT
chr5	1217676	1217825	+	1217784	1217800	33	48	+	0.667783	AAGTGCAGGATGCGCAG
chr14	65289626	65289775	+	65289649	65289665	-52	-37	+	0.667444	GGCCCCAAACAGTACCT
chr16	14380676	14380825	+	14380738	14380754	-13	2	-	0.667419	GGATTCTGGGCGTCCCT
chr16	14380626	14380775	+	14380738	14380754	37	52	-	0.667419	GGATTCTGGGCGTCCCT
chr16	1295476	1295625	+	1295574	1295590	23	38	-	0.667336	TGTCTGACGTTGTTCTC
chr1	15685176	15685325	+	15685253	15685269	2	17	-	0.667207	TAGGGCTCGATGTGCAT
chr4	184244676	184244825	+	184244743	184244759	-8	7	-	0.666973	AAGTAGAACCACTGCCC
chr7	3018526	3018675	+	3018635	3018651	34	49	-	0.6669	TTGCACTTTATCTACTA
chr1	63798276	63798425	+	63798366	63798382	15	30	+	0.66658	TGGGAACTGGTATTCTA
chr1	63798226	63798375	+	63798366	63798382	65	80	+	0.66658	TGGGAACTGGTATTCTA
chr7	4870126	4870275	+	4870214	4870230	13	28	+	0.666302	AAGCACGACATCAGCCA
chr10	26502026	26502175	+	26502119	26502135	18	33	+	0.666046	GGGCTCAGAGCGTTTGT
chr3	14595676	14595825	+	14595796	14595812	45	60	+	0.665825	ATGCAGAGCCTGATCCA
chr10	3313076	3313225	+	3313207	3313223	56	71	+	0.66569	AGCAAGCGAGTGTTCAC
chr10	131650476	131650625	+	131650498	131650514	-53	-38	-	0.665596	GAGTTAATCTCGTTACT
chr10	131650426	131650575	+	131650498	131650514	-3	12	-	0.665596	GAGTTAATCTCGTTACT
chr10	131650376	131650525	+	131650498	131650514	47	62	-	0.665596	GAGTTAATCTCGTTACT
chr7	1388126	1388275	+	1388128	1388144	-73	-58	-	0.664969	CGACACACTTCCTTCTG
chr7	1388076	1388225	+	1388128	1388144	-23	-8	-	0.664969	CGACACACTTCCTTCTG
chr4	187729076	187729225	+	187729116	187729132	-35	-20	-	0.664943	AGTGTCAACAAGTTCTT
chr4	187729026	187729175	+	187729116	187729132	15	30	-	0.664943	AGTGTCAACAAGTTCTT
chr10	4296276	4296425	+	4296300	4296316	-51	-36	-	0.664936	TGGAAGGGCAGGTCCTG
chr8	54164726	54164875	+	54164834	54164850	33	48	-	0.664909	GAGGTGACAGTGTCTCT
chr20	48124076	48124225	+	48124190	48124206	39	54	+	0.664884	AGTAACGAGGTGAATTG
chr1	1936726	1936875	+	1936796	1936812	-5	10	-	0.664287	CAGAATGCCCAGCACCC
chr1	1936676	1936825	+	1936796	1936812	45	60	-	0.664287	CAGAATGCCCAGCACCC
chr16	3142576	3142725	+	3142650	3142666	-1	14	-	0.663963	CAGCACACTCAGGAACT
chr19	40032626	40032775	+	40032711	40032727	10	25	+	0.663763	AATTTCCCTTTGTTCTG
chr11	57364876	57365025	+	57365007	57365023	56	71	-	0.663715	AGTCCCATTCCGTCCCA
chr6	41435576	41435725	+	41435693	41435709	42	57	-	0.663694	GGGTTTTCCGAGTCCCT
chr4	1504476	1504625	+	1504589	1504605	38	53	+	0.663658	GGTGAAAGAGTCTGCCC
chr3	185420276	185420425	+	185420343	185420359	-8	7	+	0.663538	AGGTAAAAGGCATTTTT
chr3	185420226	185420375	+	185420343	185420359	42	57	+	0.663538	AGGTAAAAGGCATTTTT
chr17	63134076	63134225	+	63134125	63134141	-26	-11	+	0.663338	GAAAACGGGGTGATTTG
chr21	47398576	47398725	+	47398579	47398595	-72	-57	+	0.663334	AGGAACACAAAGCAGGC
chr8	103572776	103572925	+	103572908	103572924	57	72	+	0.663052	AAGGATGTAACATACTC
chr4	1537526	1537675	+	1537565	1537581	-36	-21	+	0.662842	TAGCATTAGGTGTATCT
chr1	210501276	210501425	+	210501288	210501304	-63	-48	+	0.662798	AAGAAAAGATAGCTCCA
chr14	76877876	76878025	+	76877972	76877988	21	36	+	0.662566	GGTGGCAGCACGTGCTG
chr9	132482376	132482525	+	132482515	132482531	64	79	-	0.66252	AGCAAGAGCCTCTCCCC
chr9	137660326	137660475	+	137660434	137660450	33	48	+	0.662418	AAGACCCGTTTGGTCTC
chr9	137859526	137859675	+	137859605	137859621	4	19	-	0.662395	GAGGAGGCCAGGCTCCT
chr9	137859476	137859625	+	137859605	137859621	54	69	-	0.662395	GAGGAGGCCAGGCTCCT
chr20	23969976	23970125	+	23970002	23970018	-49	-34	+	0.662332	GGATGCACGGTCTGCAT
chr8	81963276	81963425	+	81963413	81963429	62	77	-	0.662142	CTGAAGGCTCTGCTCTT
chr5	2205726	2205875	+	2205729	2205745	-72	-57	+	0.662078	AGGAGCCCCTGGTGCTG
chr5	2205676	2205825	+	2205729	2205745	-22	-7	+	0.662078	AGGAGCCCCTGGTGCTG
chr5	2205626	2205775	+	2205729	2205745	28	43	+	0.662078	AGGAGCCCCTGGTGCTG
chr10	131034726	131034875	+	131034857	131034873	56	71	-	0.662019	TTTAACGCCATCTCCTT
chr22	43621676	43621825	+	43621722	43621738	-29	-14	+	0.661947	AGAAACTAAATGTGTCA
chr7	158828176	158828325	+	158828311	158828327	60	75	+	0.661891	GGGTAAACAACATGCAG
chr10	3591176	3591325	+	3591228	3591244	-23	-8	+	0.661706	GTGTACTGGAAGTTGCT
chr10	3591126	3591275	+	3591228	3591244	27	42	+	0.661706	GTGTACTGGAAGTTGCT

Supplementary Table S7 (con'd)

chr10	131650276	131650425	+	131650298	131650314	-53	-38	-	0.661638	AATCACATTCCATGCCA
chr10	131650226	131650375	+	131650298	131650314	-3	12	-	0.661638	AATCACATTCCATGCCA
chr2	209271126	209271275	+	209271160	209271176	-41	-26	+	0.661556	AGACGCAGGGCCTACCT
chr2	209271076	209271225	+	209271160	209271176	9	24	+	0.661556	AGACGCAGGGCCTACCT
chr2	209271026	209271175	+	209271160	209271176	59	74	+	0.661556	AGACGCAGGGCCTACCT
chr5	2206676	2206825	+	2206702	2206718	-49	-34	+	0.661241	GAGCACACAGACTTTAC
chr15	27210226	27210375	+	27210351	27210367	50	65	+	0.661142	AGAGCCATTGGGTGCTT
chr10	3479876	3480025	+	3480002	3480018	51	66	-	0.661093	CTGTAGACCAAGTGCAC
chr7	127881476	127881625	+	127881490	127881506	-61	-46	+	0.66102	ATGATAGCTTTGTACCG
chr7	1137276	1137425	+	1137289	1137305	-62	-47	+	0.660871	GACTGCAGAGCCTACCT
chr7	1137226	1137375	+	1137289	1137305	-12	3	+	0.660871	GACTGCAGAGCCTACCT
chr11	63996726	63996875	+	63996730	63996746	-71	-56	-	0.660515	CTGCTCACCCTGAGCCA
chr11	63996676	63996825	+	63996730	63996746	-21	-6	-	0.660515	CTGCTCACCCTGAGCCA
chr7	95154976	95155125	+	95155009	95155025	-42	-27	+	0.66049	GGGAAGACATTCTCATT
chr17	79459326	79459475	+	79459447	79459463	46	61	-	0.660466	CGCTTCCCTGGGTTCCT
chr17	1202176	1202325	+	1202303	1202319	52	67	+	0.660395	GGTCCCAGGATGTCTCC
chr4	6575726	6575875	+	6575788	6575804	-13	2	+	0.660313	GTGGACAGGGCTGACTC
chr3	23782776	23782925	+	23782915	23782931	64	79	-	0.660204	TGGTGCCCTTCTTACCT
chr1	33391376	33391525	+	33391514	33391530	63	78	-	0.659986	CAGGACTCAGTCCTCCA
chr15	27210176	27210325	+	27210314	27210330	63	78	+	0.65991	GGGTCTACGGTGTTGGA
chr10	94448426	94448575	+	94448461	94448477	-40	-25	-	0.65987	AGGGACTACATCTGTCC
chr17	1811276	1811425	+	1811333	1811349	-18	-3	+	0.659467	AACAGCAGAGTGAGCCG
chr1	87994626	87994775	+	87994641	87994657	-60	-45	+	0.659457	GAGAGCTGATTGGTCCA
chr1	87994576	87994725	+	87994641	87994657	-10	5	+	0.659457	GAGAGCTGATTGGTCCA
chr19	45720026	45720175	+	45720034	45720050	-67	-52	-	0.659282	AAGCTCCCTGCATTCTT
chr14	103691376	103691525	+	103691405	103691421	-46	-31	+	0.659086	AGGGTCCTTGCGTATTT
chr12	107297226	107297375	+	107297320	107297336	19	34	+	0.659008	TCGTCCGGTGTGTTTTC
chr9	138109176	138109325	+	138109287	138109303	36	51	-	0.658965	GAAAATACTTTCTGCTT
chr19	18902626	18902775	+	18902726	18902742	25	40	-	0.658838	CGGGAAACAGAGTCACC
chr9	136500076	136500225	+	136500145	136500161	-6	9	-	0.658712	GAGAGCACTGCCTGCGA
chr10	135054876	135055025	+	135054908	135054924	-43	-28	+	0.658474	AAGGTCTGGAAGTGCCC
chr10	135054826	135054975	+	135054908	135054924	7	22	+	0.658474	AAGGTCTGGAAGTGCCC
chr7	1251076	1251225	+	1251155	1251171	4	19	-	0.658473	CAGCAGATCCTGGTGCC
chr1	14220226	14220375	+	14220263	14220279	-38	-23	-	0.658206	TCGTGTGTCATGTGCCC
chr1	14220176	14220325	+	14220263	14220279	12	27	-	0.658206	TCGTGTGTCATGTGCCC
chr1	14220126	14220275	+	14220263	14220279	62	77	-	0.658206	TCGTGTGTCATGTGCCC
chr22	43805226	43805375	+	43805315	43805331	14	29	-	0.658016	TGGAAGGTGAGTTTCTT
chr22	43805176	43805325	+	43805315	43805331	64	79	-	0.658016	TGGAAGGTGAGTTTCTT
chr6	45500826	45500975	+	45500927	45500943	26	41	+	0.657533	AGTGAAAGGAGGTTCTA
chr1	38605976	38606125	+	38606104	38606120	53	68	-	0.657449	AACATCTCCATGTGCAC
chr3	72704526	72704675	+	72704568	72704584	-33	-18	+	0.657378	TAGCACGTGGGCTCCTG
chr16	88880726	88880875	+	88880791	88880807	-10	5	+	0.657309	AGGCACTTGCAGGGCCA
chr1	156831076	156831225	+	156831189	156831205	38	53	-	0.657292	GAAATCTCCGTGGACCA
chr10	4331726	4331875	+	4331734	4331750	-67	-52	-	0.656636	AGGATCATTGGATAACT
chr1	7569226	7569375	+	7569258	7569274	-43	-28	+	0.656617	AAGGAGAGTGTGTTGGA
chr19	46651126	46651275	+	46651132	46651148	-69	-54	+	0.656194	ATGAATATGAAGTTTCA
chr11	71010376	71010525	+	71010418	71010434	-33	-18	+	0.656168	AAGCGTGGCTTGTTCTG
chr17	79109626	79109775	+	79109683	79109699	-18	-3	+	0.65596	GGAAACCAGCTCTGCCG
chr15	99088026	99088175	+	99088149	99088165	48	63	-	0.655604	CAGACCCCCGTCTACAC
chr3	169540026	169540175	+	169540072	169540088	-29	-14	+	0.65559	GAGCTCCGGCTCTACCA
chr11	64780626	64780775	+	64780730	64780746	29	44	-	0.655533	AGTTAGCTATTGTCCTC
chr1	25298626	25298775	+	25298733	25298749	32	47	-	0.655431	CAGAGCCCTCTGAGCTC
chr12	322526	322675	+	322559	322575	-42	-27	+	0.655293	GGGTACAAGCACACCCA
chr11	69706676	69706825	+	69706708	69706724	-43	-28	+	0.655282	GGGTCACCAATTTTCCT
chr17	66288676	66288825	+	66288803	66288819	52	67	+	0.65489	GAGGAGTCGGCGATCTG
chr15	41219326	41219475	+	41219380	41219396	-21	-6	-	0.654447	TAGATCTCTGGGATCTC
chr15	29825276	29825425	+	29825332	29825348	-19	-4	+	0.654356	GGGGAAACTGAGGCCTG
chr1	1084476	1084625	+	1084499	1084515	-52	-37	-	0.654182	CAGAAGAAGCAGCACCT
chr1	1084426	1084575	+	1084499	1084515	-2	13	-	0.654182	CAGAAGAAGCAGCACCT
chr9	122800826	122800975	+	122800932	122800948	31	46	-	0.653593	GCTTCCACCATGTCCAC
chr4	3677526	3677675	+	3677665	3677681	64	79	+	0.653577	AGGGACAGGCCCAGCTG
chr10	3457276	3457425	+	3457362	3457378	11	26	+	0.653441	CGGGGCGGAGTGGGCCA
chr16	57317576	57317725	+	57317602	57317618	-49	-34	-	0.653423	GAGTAGGAGTCTTGCCC
chr5	1923426	1923575	+	1923459	1923475	-42	-27	-	0.653314	GGCTACATTTTGGCATC
chr16	1316276	1316425	+	1316346	1316362	-5	10	-	0.653224	AGGTTCTACAAGTTGCC
chr15	23894676	23894825	+	23894704	23894720	-47	-32	-	0.653142	ACTTTCACCTTTTTCCT
chr20	36037626	36037775	+	36037674	36037690	-27	-12	-	0.652789	TGGTCCAGCCTCAGCCT
chr16	88963576	88963725	+	88963677	88963693	26	41	+	0.65246	AGGACAGCCCTGTGCAA
chr10	3480026	3480175	+	3480050	3480066	-51	-36	+	0.652443	CGGGACAGAAAGATTTC
chr8	42009076	42009225	+	42009212	42009228	61	76	+	0.652225	CAGCACAGGTCGATACG
chr4	3865026	3865175	+	3865132	3865148	31	46	+	0.652217	GGGCCAAGCTTTTTCTC
chr14	104768276	104768425	+	104768318	104768334	-33	-18	-	0.651851	GGACCGGCCCTGTTCTC
chr10	4414876	4415025	+	4414945	4414961	-6	9	+	0.651814	AGGACTGTTTTGTTGCT
chr17	25583176	25583325	+	25583210	25583226	-41	-26	-	0.651814	ATAATCATGCTTTTCCT
chr2	71099176	71099325	+	71099221	71099237	-30	-15	-	0.651202	AAGTGCTTAATATTCAC
chr7	36013226	36013375	+	36013253	36013269	-48	-33	+	0.651122	AAGAAAGGGCAGATCTT
chr16	2863776	2863925	+	2863795	2863811	-56	-41	-	0.650998	AAGTACTTAGAGCAGCC
chr1	6531226	6531375	+	6531277	6531293	-24	-9	+	0.650711	AGCAAGCTTATGATCCC
chr10	79270626	79270775	+	79270641	79270657	-60	-45	+	0.650254	ATGGAGATGACTTTCCA
chr11	120590026	120590175	+	120590053	120590069	-48	-33	-	0.650245	GGGTTAACCAGGTGGCT
chr11	120589976	120590125	+	120590053	120590069	2	17	-	0.650245	GGGTTAACCAGGTGGCT
chr2	42077526	42077675	+	42077658	42077674	57	72	-	0.650145	TGGATGAGGGCGGTCTT
chr16	85198476	85198625	+	85198582	85198598	31	46	+	0.650099	AGGGAAGTCGGGTTGCA
chr10	3276976	3277125	+	3277052	3277068	1	16	+	0.649895	AGGACCGGGATCTTTTG
chr8	1707176	1707325	+	1707253	1707269	2	17	-	0.649888	TGCTACAGAGTCTGTTT
chr7	1407226	1407375	+	1407249	1407265	-52	-37	+	0.649818	GTTTAAATGATGTTTTT
chr7	1407176	1407325	+	1407249	1407265	-2	13	+	0.649818	GTTTAAATGATGTTTTT
chr6	134350726	134350875	+	134350788	134350804	-13	2	-	0.649767	AAGTTCATAACCCACCA
chr1	9341876	9342025	+	9341894	9341910	-57	-42	-	0.649675	AGGATCTCCTGCTACTT
chr6	25726926	25727075	+	25726945	25726961	-56	-41	+	0.649643	CCGAACGCGGCGTTTGA
chr7	3019026	3019175	+	3019111	3019127	10	25	-	0.6496	GGGGTCACCCCGAAATC
chr20	61162126	61162275	+	61162150	61162166	-51	-36	+	0.649528	TGGAACCAAATCGACTG
chr10	4378526	4378675	+	4378635	4378651	34	49	+	0.649358	TGAAACTTGATGTTAAT
chr12	58736226	58736375	+	58736313	58736329	12	27	-	0.649354	AGCGAGGTCATTTTCCC
chr7	1388176	1388325	+	1388189	1388205	-62	-47	-	0.648723	AATTTTACCGTTTTCTT
chr1	88108726	88108875	+	88108864	88108880	63	78	+	0.648385	TAGCCCAGAAAATTCCT
chr6	25727276	25727425	+	25727385	25727401	34	49	-	0.648054	CACTACAGCAAGCGCTC
chr20	23970026	23970175	+	23970040	23970056	-61	-46	-	0.647554	GAGGCCTCTCTCTCCCT
chr11	14994276	14994425	+	14994315	14994331	-36	-21	-	0.647446	GGGTGGGTTCTGGGCCC
chr11	14994226	14994375	+	14994315	14994331	14	29	-	0.647446	GGGTGGGTTCTGGGCCC
chr21	46816576	46816725	+	46816579	46816595	-72	-57	+	0.647184	ATGGGCGCATTGCACCT

Supplementary Table S7 (con’d)

chr21	46816526	46816675	+	46816579	46816595	-22	-7	+	0.647184	ATGGGCGCATTGCACCT
chr6	25727226	25727375	+	25727316	25727332	15	30	+	0.64671	ATGAGCATTATGAATTC
chr6	25727176	25727325	+	25727316	25727332	65	80	+	0.64671	ATGAGCATTATGAATTC
chr14	106437976	106438125	+	106438014	106438030	-37	-22	-	0.646573	GTGAATGACGTGTCTTT
chr3	64224976	64225125	+	64225046	64225062	-5	10	+	0.645945	GGAGACATATTCTGCAA
chr22	32750776	32750925	+	32750875	32750891	24	39	+	0.645432	AGCAGCGAGGTGAGCCA
chr15	23894726	23894875	+	23894803	23894819	2	17	-	0.645372	TAAAACAAGGGTTCCTT
chr6	18990476	18990625	+	18990539	18990555	-12	3	+	0.645367	AAGGAGAAAGTGATATA
chr6	18990426	18990575	+	18990539	18990555	38	53	+	0.645367	AAGGAGAAAGTGATATA
chr11	102216826	102216975	+	102216909	102216925	8	23	+	0.645244	GGGCAAAGAGCGTCAAC
chr11	102216776	102216925	+	102216909	102216925	58	73	+	0.645244	GGGCAAAGAGCGTCAAC
chr1	7408776	7408925	+	7408871	7408887	20	35	+	0.645074	ACGTTCACTGTATTACG
chr12	107297176	107297325	+	107297290	107297306	39	54	-	0.645013	AATTCCGCCCCGGACCC
chr5	149683226	149683375	+	149683333	149683349	32	47	+	0.644832	CGGACCAGTGTTTCCAG
chr14	104688476	104688625	+	104688529	104688545	-22	-7	-	0.644726	GGGTTGTCTCTGTGCGT
chr14	104688426	104688575	+	104688529	104688545	28	43	-	0.644726	GGGTTGTCTCTGTGCGT
chr16	84336176	84336325	+	84336219	84336235	-32	-17	-	0.644474	GAGCCCAGCTCTTCCTT
chr9	129387126	129387275	+	129387244	129387260	43	58	+	0.644376	AATAAGATTATGTCACG
chr16	84336226	84336375	+	84336313	84336329	12	27	-	0.644103	AAAAGCAATTCGTTCGC
chr7	3018226	3018375	+	3018251	3018267	-50	-35	+	0.64396	GGGCACCGGGTGCAGGC
chr21	34350976	34351125	+	34351092	34351108	41	56	-	0.643876	GAGAAAGGCCTGGAGCC
chr11	62100726	62100875	+	62100854	62100870	53	68	-	0.643717	CAGCCGCTTGTGTGCCT
chr10	26501976	26502125	+	26502035	26502051	-16	-1	-	0.643475	GGGTGCCCTTGGCTCCT
chr16	49530526	49530675	+	49530618	49530634	17	32	+	0.642854	GGCTACGGCAAATGCTC
chr10	134662176	134662325	+	134662230	134662246	-21	-6	-	0.642764	CAGCCCACTCGGGCCCC
chr19	18902576	18902725	+	18902648	18902664	-3	12	-	0.64255	GAGTGCTTGCTGTATGC
chr3	55931376	55931525	+	55931438	55931454	-13	2	+	0.642216	ACAGACAGGCTCTTCTG
chr3	55931326	55931475	+	55931438	55931454	37	52	+	0.642216	ACAGACAGGCTCTTCTG
chr17	7082926	7083075	+	7082967	7082983	-34	-19	-	0.642192	CAGAACTATTGGTGTCT
chr17	7082876	7083025	+	7082967	7082983	16	31	-	0.642192	CAGAACTATTGGTGTCT
chr19	554776	554925	+	554842	554858	-9	6	+	0.642164	GTGAACTTATTTTACAA
chr5	1010826	1010975	+	1010870	1010886	-31	-16	+	0.642158	AGCAGCCAGGTGCTCCT
chr5	1010776	1010925	+	1010870	1010886	19	34	+	0.642158	AGCAGCCAGGTGCTCCT
chr5	532876	533025	+	532883	532899	-68	-53	-	0.642064	GGGAGCCCCGCCTTGCC
chr5	532826	532975	+	532883	532899	-18	-3	-	0.642064	GGGAGCCCCGCCTTGCC
chr11	68781826	68781975	+	68781929	68781945	28	43	-	0.642048	TGGAACGCTTGTTTACA
chr5	2207026	2207175	+	2207153	2207169	52	67	+	0.641807	AAGAACCCTGTTTATGG
chr10	34496226	34496375	+	34496243	34496259	-58	-43	+	0.64133	CAGCAGCAAGTGTTTTC
chr11	69706726	69706875	+	69706755	69706771	-46	-31	-	0.641327	TGGACAGTAAGGTGCCT
chr16	876026	876175	+	876060	876076	-41	-26	+	0.641287	GGTAAGATATTTTGCTG
chr7	45188076	45188225	+	45188209	45188225	58	73	-	0.641082	GACTTCGGCATGGGCCT
chr3	72704626	72704775	+	72704664	72704680	-37	-22	+	0.640834	CGGACAATTTTGAACTG
chr3	72704576	72704725	+	72704664	72704680	13	28	+	0.640834	CGGACAATTTTGAACTG
chr11	1102426	1102575	+	1102502	1102518	1	16	+	0.640689	ACAGACGCACTGTATCA
chr10	3500076	3500225	+	3500085	3500101	-66	-51	-	0.640501	TGTCACACACTGAGATC
chr8	700076	700225	+	700117	700133	-34	-19	+	0.640471	GTGGAAGGGCTTTTCCT
chr8	700026	700175	+	700117	700133	16	31	+	0.640471	GTGGAAGGGCTTTTCCT
chr1	57718926	57719075	+	57718936	57718952	-65	-50	+	0.640099	AGTGACTGTTAGTTCTA
chr10	3378976	3379125	+	3379045	3379061	-6	9	+	0.640078	GGATAAAAGCTGACCCG
chr5	493226	493375	+	493331	493347	30	45	+	0.640041	GGGATAATGAGCTTCTT
chr12	52238576	52238725	+	52238616	52238632	-35	-20	-	0.639402	AGGCTGGCCTAGTGCCT
chr12	52238526	52238675	+	52238616	52238632	15	30	-	0.639402	AGGCTGGCCTAGTGCCT
chr20	4705126	4705275	+	4705200	4705216	-1	14	+	0.639337	AGGAAGCACCTGAGCTG
chr21	43547726	43547875	+	43547815	43547831	14	29	+	0.639049	GAGAACGGCAACTCCAA
chr21	43547676	43547825	+	43547815	43547831	64	79	+	0.639049	GAGAACGGCAACTCCAA
chr10	131650676	131650825	+	131650748	131650764	-3	12	+	0.639019	AGGCACTGGGATTTTCT
chr10	131650626	131650775	+	131650748	131650764	47	62	+	0.639019	AGGCACTGGGATTTTCT
chr15	29037776	29037925	+	29037825	29037841	-26	-11	+	0.638952	TGAAAAATATTTTTCTG
chr16	22775926	22776075	+	22775935	22775951	-66	-51	-	0.638545	AAGAAAGCTACCTTGCT
chr13	28563576	28563725	+	28563659	28563675	8	23	+	0.638529	TGGACTCCAGTGATCCA
chr8	144854576	144854725	+	144854619	144854635	-32	-17	+	0.638481	GTGTACGCAGAGGACGG
chr16	29267676	29267825	+	29267809	29267825	58	73	-	0.638456	CAAGACATGGCGTTTGT
chr16	281276	281425	+	281395	281411	44	59	-	0.638354	AGAAAAGGCACGTTTCC
chr9	6716226	6716375	+	6716229	6716245	-72	-57	-	0.637996	CGTTCCTCCGTTTCCCC
chr11	120764426	120764575	+	120764566	120764582	65	80	-	0.637708	TGTTAATCTCTGCACCC
chr17	75848676	75848825	+	75848780	75848796	29	44	+	0.637621	TGGAGCACGCAGCCCTG
chr6	37014476	37014625	+	37014488	37014504	-63	-48	+	0.637451	AGGTAAAATAAGGTCAT
chr12	107297076	107297225	+	107297205	107297221	54	69	+	0.637419	GACCACCCAGCCTGCTC
chr19	47735626	47735775	+	47735663	47735679	-38	-23	-	0.637263	TAGAAGAAACTGACCAC
chr21	42219676	42219825	+	42219774	42219790	23	38	+	0.637235	AGGGCTGGAATGCACCC
chr9	132383226	132383375	+	132383319	132383335	18	33	-	0.636975	AATAGCAGCACCTGCCT
chr5	1207376	1207525	+	1207505	1207521	54	69	+	0.636696	GGGCGAGTGGAGTTCAT
chr1	210612176	210612325	+	210612291	210612307	40	55	+	0.636369	ATGGACCTCATGATCAG
chr5	141993176	141993325	+	141993205	141993221	-46	-31	+	0.635971	GGGGCCAGATCTTTCAC
chr20	1164876	1165025	+	1164996	1165012	45	60	-	0.635741	AGTTACACAAAGGCCGT
chr16	1198576	1198725	+	1198634	1198650	-17	-2	-	0.635465	TAGACGGCGCTGTCCAC
chr16	1198526	1198675	+	1198634	1198650	33	48	-	0.635465	TAGACGGCGCTGTCCAC
chr4	3690826	3690975	+	3690909	3690925	8	23	+	0.635187	GAAGAGGCGGTGTGGTC
chr5	162997826	162997975	+	162997853	162997869	-48	-33	-	0.635128	AACTATAAAATTTGCTT
chr7	104897076	104897225	+	104897083	104897099	-68	-53	-	0.63478	AATGAGCCACTGTGCTC
chr17	74581226	74581375	+	74581260	74581276	-41	-26	+	0.634143	GGGCGCGGGGCGGACAG
chr3	97542226	97542375	+	97542278	97542294	-23	-8	-	0.633344	TGTTCCTCGGTTTCCCC
chr15	101807276	101807425	+	101807394	101807410	43	58	-	0.633264	GGGTAACTCTCTTGCTT
chr11	128796326	128796475	+	128796399	128796415	-2	13	-	0.633221	GGCAAAACCTTCATCCT
chr3	185866476	185866625	+	185866549	185866565	-2	13	-	0.632817	GAGTTCTCGGTCTCTTC
chr19	47735676	47735825	+	47735704	47735720	-47	-32	-	0.632121	TGTGACAGCTTCTTGCT
chr5	173737976	173738125	+	173738067	173738083	16	31	+	0.631909	GGGGTGGGCAGGTTCCA
chr11	2210126	2210275	+	2210148	2210164	-53	-38	-	0.631714	GAGAAATCAGAGCCCCT
chr14	101128326	101128475	+	101128332	101128348	-69	-54	-	0.631607	AAAAGCACCCTAGACCT
chr14	101128276	101128425	+	101128332	101128348	-19	-4	-	0.631607	AAAAGCACCCTAGACCT
chr10	3572226	3572375	+	3572334	3572350	33	48	+	0.631352	TGGGAAAGTCAGCACTC
chr15	99087976	99088125	+	99088072	99088088	21	36	+	0.631206	TTGACCATGAAATACCA

Supplementary Table S7 (con'd)

chr2	202753026	202753175	+	202753118	202753134	17	32	-	0.630757	GATGACTGAATTTCCCT
chr2	11294426	11294575	+	11294465	11294481	-36	-21	+	0.630625	CAGCACCTGTTTTTTTT
chr16	29241926	29242075	+	29241974	29241990	-27	-12	-	0.630598	TGGCACAGCCAAACCCC
chr17	65527576	65527725	+	65527714	65527730	63	78	+	0.630526	TGAAACTAACTGCACTG
chr12	52240276	52240425	+	52240277	52240293	-74	-59	-	0.630461	CATAAAAAGCTGTGGCC
chr12	52240226	52240375	+	52240277	52240293	-24	-9	-	0.630461	CATAAAAAGCTGTGGCC
chr16	57317626	57317775	+	57317645	57317661	-56	-41	-	0.630274	AGCGATATCTCGGTCCC
chr10	118084226	118084375	+	118084254	118084270	-47	-32	+	0.630166	AAGCCCGGTTTGTAAAC
chr11	67462676	67462825	+	67462772	67462788	21	36	-	0.630134	CAGAATATCCCGACCAC
chr16	1111076	1111225	+	1111129	1111145	-22	-7	-	0.63008	CAGCACCCACTCTCACT
chr4	3677476	3677625	+	3677601	3677617	50	65	-	0.630008	GCGGACACCCGGGAACC
chr17	25798326	25798475	+	25798327	25798343	-74	-59	-	0.629929	CAGACAGACGTGCTCTC
chr4	1160676	1160825	+	1160702	1160718	-49	-34	+	0.629818	GGGGACAGCCAGGGACA
chr6	25727076	25727225	+	25727098	25727114	-53	-38	+	0.62958	AAAAGAACCGTGTAACG
chr13	50703376	50703525	+	50703464	50703480	13	28	-	0.629133	TAAAACGTTATGCGTCT
chr2	11294476	11294625	+	11294491	11294507	-60	-45	+	0.628552	GCGGAGGTGATGGGCCG
chr22	37215876	37216025	+	37215949	37215965	-2	13	+	0.628528	AGGTAGACATAGCTGCT
chr14	23290276	23290425	+	23290300	23290316	-51	-36	-	0.628377	CAGAAATCATAGTACAC
chr14	23290226	23290375	+	23290300	23290316	-1	14	-	0.628377	CAGAAATCATAGTACAC
chr3	126080226	126080375	+	126080311	126080327	10	25	+	0.628183	AGGAGCAGGAAGAGACC
chr3	169540276	169540425	+	169540277	169540293	-74	-59	+	0.62697	TGGAAATCATTGACCTG
chr7	29186226	29186375	+	29186238	29186254	-63	-48	-	0.626717	CCCAACCTCCTGGTCCC
chr10	130959576	130959725	+	130959675	130959691	24	39	+	0.626176	AGCACCCACGTGTAACA
chr2	202753176	202753325	+	202753243	202753259	-8	7	+	0.625963	TGGAAGGTTTTGCATTT
chr14	106095376	106095525	+	106095480	106095496	29	44	-	0.624966	GAGCTCAGGTCGGTCGT
chr7	2728726	2728875	+	2728847	2728863	46	61	+	0.624634	CAGAGCATGGTTTCTTG
chr22	19743976	19744125	+	19744079	19744095	28	43	+	0.624388	GAGGAGCAGATGTCTCA
chr13	28562426	28562575	+	28562456	28562472	-45	-30	-	0.624313	GGGCTCAAGTGATCCTC
chr16	876076	876225	+	876175	876191	24	39	-	0.624069	TACAACTCAGCGTTTGC
chr19	35818776	35818925	+	35818842	35818858	-9	6	-	0.623746	CAATGCTGTCTGTTCTG
chr19	35818726	35818875	+	35818842	35818858	41	56	-	0.623746	CAATGCTGTCTGTTCTG
chr7	1747926	1748075	+	1747942	1747958	-59	-44	+	0.623692	GAGAATCCTCAGCACCA
chr7	1747876	1748025	+	1747942	1747958	-9	6	+	0.623692	GAGAATCCTCAGCACCA
chr7	1329226	1329375	+	1329271	1329287	-30	-15	+	0.623404	GGGCCCATTTCTTTTTT
chr10	13039576	13039725	+	13039636	13039652	-15	0	-	0.622994	CAGCACTTTTCCTTTCC
chr12	107297026	107297175	+	107297028	107297044	-73	-58	+	0.622992	AGATCAAAAAAGTTCCT
chr12	107296976	107297125	+	107297028	107297044	-23	-8	+	0.622992	AGATCAAAAAAGTTCCT
chr19	51538076	51538225	+	51538184	51538200	33	48	-	0.622895	TCTACCACTCTGCACCT
chr17	81036076	81036225	+	81036193	81036209	42	57	+	0.622654	AGACTCACAATTTTCAA
chr10	73324326	73324475	+	73324349	73324365	-52	-37	+	0.622338	AGGACCATGCGGTATGG
chr3	129326576	129326725	+	129326702	129326718	51	66	-	0.622272	GTGAGGACCATGCGCCG
chr17	7082976	7083125	+	7083005	7083021	-46	-31	-	0.622233	CAGAGGGACCGGTTCTC
chr10	131650576	131650725	+	131650625	131650641	-26	-11	-	0.622231	CAGTTCAACTTGGGATT
chr10	131650526	131650675	+	131650625	131650641	24	39	-	0.622231	CAGTTCAACTTGGGATT
chr12	118312276	118312425	+	118312340	118312356	-11	4	-	0.62212	TTGTAAATATCATTCTA
chr4	1535626	1535775	+	1535695	1535711	-6	9	+	0.62185	AAGGTCACCGTCAACAG
chr4	1535576	1535725	+	1535695	1535711	44	59	+	0.62185	AAGGTCACCGTCAACAG
chr20	36037676	36037825	+	36037686	36037702	-65	-50	-	0.621737	AGCCTCAAGGCTTGCCT
chr1	3507026	3507175	+	3507140	3507156	39	54	-	0.621135	GCCTACTCCTCGCTCCT
chr4	3690676	3690825	+	3690707	3690723	-44	-29	-	0.620909	TTGTGAAAAATGATCCC
chr10	112834776	112834925	+	112834890	112834906	39	54	+	0.620836	GAGCAGAGACTTTAGTA
chr1	226791276	226791425	+	226791316	226791332	-35	-20	+	0.62068	TGGGAAGTAGTGGCCAC
chr14	104768426	104768575	+	104768433	104768449	-68	-53	-	0.620472	AAACACACCACGACTTT
chr14	104768376	104768525	+	104768433	104768449	-18	-3	-	0.620472	AAACACACCACGACTTT
chr22	19743926	19744075	+	19744040	19744056	39	54	+	0.620272	GAGAAATGGGCGTCTTG
chr19	49528526	49528675	+	49528537	49528553	-64	-49	+	0.619949	AACCACATGTGGTGGCA
chr19	49528476	49528625	+	49528537	49528553	-14	1	+	0.619949	AACCACATGTGGTGGCA
chr15	68699576	68699725	+	68699654	68699670	3	18	-	0.619431	AATTACAAAGTGCTGGT
chr15	68699526	68699675	+	68699654	68699670	53	68	-	0.619431	AATTACAAAGTGCTGGT
chr6	130992626	130992775	+	130992704	130992720	3	18	+	0.619084	GAGCAGACGAGTAACTT
chr1	9341926	9342075	+	9342044	9342060	43	58	+	0.618991	ATGTCCAGGTCCTGCCA
chr10	118084276	118084425	+	118084361	118084377	10	25	-	0.618018	GGCTCCATCTTCTTGCT
chr1	226791326	226791475	+	226791436	226791452	35	50	-	0.6179	AGCTACTGTCCGTCTTC
chr17	81036026	81036175	+	81036053	81036069	-48	-33	-	0.617564	CTGAACAGATCCTTACA
chr21	46420426	46420575	+	46420429	46420445	-72	-57	-	0.617402	GTGACCACCCCTTTGTC
chr10	131752776	131752925	+	131752899	131752915	48	63	+	0.617126	GTGGGCCCATTGATCTC
chr1	55504626	55504775	+	55504737	55504753	36	51	-	0.616934	AGGAGCAGCTAGTTGGT
chr10	4386676	4386825	+	4386729	4386745	-22	-7	-	0.616684	TTCAACAGATCATTCCA
chr8	81963226	81963375	+	81963262	81963278	-39	-24	+	0.615925	AGATAAAGCTTATACAT
chr14	101123426	101123575	+	101123558	101123574	57	72	-	0.615676	AGGCAGGCTCTGGGTTC
chr22	37499376	37499525	+	37499427	37499443	-24	-9	+	0.614353	AGCCACCTGGGGGGCCT
chr10	131650326	131650475	+	131650461	131650477	60	75	+	0.614225	GGGAAGGATACATATTT
chr8	144367176	144367325	+	144367218	144367234	-33	-18	-	0.613893	AGGTGCACTCGCACCCT
chr6	37527226	37527375	+	37527232	37527248	-69	-54	+	0.612857	AGGCAGAGCTGGAGCTG
chr12	6933126	6933275	+	6933160	6933176	-41	-26	-	0.611805	TGGATCATCCTCAGCAT
chr7	1328976	1329125	+	1329065	1329081	14	29	-	0.611154	AGGGACACCAGCTCTAT
chr17	25798876	25799025	+	25798981	25798997	30	45	-	0.610396	TGAAACTCAGTTCTCTG
chr5	137224876	137225025	+	137224895	137224911	-56	-41	+	0.610097	GAGAAGCCCGTGATAAT
chr11	45392426	45392575	+	45392551	45392567	50	65	-	0.608851	CAGTACAGTTATTGCGG
chr21	42219626	42219775	+	42219670	42219686	-31	-16	+	0.607964	ATTTCCTGAATGTTCTG
chr1	234039826	234039975	+	234039923	234039939	22	37	+	0.607799	AGGAAAAAGATGAGAAG
chr4	1160726	1160875	+	1160855	1160871	54	69	-	0.607587	CAGCACAGCCTAGAGCA
chr2	66743676	66743825	+	66743807	66743823	56	71	+	0.606606	GGTTGTAGAATCTTCCA
chr9	129387076	129387225	+	129387214	129387230	63	78	-	0.604888	ATGAAAAGGATTTCGCC
chr5	137225126	137225275	+	137225236	137225252	35	50	+	0.604621	AGGGACAGGGGCGATTT
chr22	43829676	43829825	+	43829736	43829752	-15	0	-	0.604372	TGGCACCGCTTTTGGTT
chr5	137225076	137225225	+	137225188	137225204	37	52	-	0.602888	GGCGTCACGGTGGCACC
chr5	137224926	137225075	+	137224999	137225015	-2	13	+	0.602796	GGGCGAAAGGCCTCCCG
chr10	4378476	4378625	+	4378596	4378612	45	60	+	0.602635	AAGTAAGATGCCTAACA
chr7	99067126	99067275	+	99067144	99067160	-57	-42	+	0.600841	AGGCAGGTGAATTTTCC
chr4	1537176	1537325	+	1537305	1537321	54	69	+	0.599988	GGAGCCGAGGAGATCCC
chr5	493176	493325	+	493182	493198	-69	-54	+	0.598359	AGGCACCAACCTATCTG
chr14	104623476	104623625	+	104623611	104623627	60	75	+	0.594962	CCGTACGTGGGGCAATT
chr12	52240326	52240475	+	52240392	52240408	-9	6	+	0.593491	AGTTACGGGATCCTCAG
chr1	218537326	218537475	+	218537398	218537414	-3	12	-	0.593012	AGACAATTCTTGTTTTT
chr5	162997876	162998025	+	162997958	162997974	7	22	-	0.591916	AAATGCAAACCGTCCAA
chr19	1505826	1505975	+	1505941	1505957	40	55	+	0.591863	GGAAATACGTTGACGTT
chr8	143261876	143262025	+	143261923	143261939	-28	-13	+	0.587376	GAGCAATATTTGACCTA
chr5	137225026	137225175	+	137225069	137225085	-32	-17	-	0.584956	AGGAGCTGGGGGCTCGC
chr13	28562476	28562625	+	28562480	28562496	-71	-56	+	0.583806	AGCCTCCCAAAGTGCTG
chr6	25761526	25761675	+	25761627	25761643	26	41	-	0.56754	CTGAAATTGATGCTTCT

Supplementary Table S8

	copy number aberration (CNA) regions	non-CNA regions
ct-MethSig	35	965
All segments	1031	236479
ar-MethSig	0	1000
All segments	1031	236479
